# A general method for the development of multicolor biosensors with large dynamic ranges

**DOI:** 10.1038/s41589-023-01350-1

**Published:** 2023-06-08

**Authors:** Lars Hellweg, Anna Edenhofer, Lucas Barck, Magnus-Carsten Huppertz, Michelle. S. Frei, Miroslaw Tarnawski, Andrea Bergner, Birgit Koch, Kai Johnsson, Julien Hiblot

**Affiliations:** 1 Department of Chemical Biology, Max Planck Institute for Medical Research, Heidelberg, Germany; 2 Protein Expression and Characterization Facility, Max Planck Institute for Medical Research, Heidelberg, Germany; 3 Institute of Chemical Sciences and Engineering (ISIC), École Polytechnique Fédérale de Lausanne (EPFL), Lausanne, Switzerland

**Keywords:** Chemical modification, Protein design, Chemical tools

## Abstract

Fluorescent biosensors enable the study of cell physiology with spatiotemporal resolution; yet, most biosensors suffer from relatively low dynamic ranges. Here, we introduce a family of designed Förster resonance energy transfer (FRET) pairs with near-quantitative FRET efficiencies based on the reversible interaction of fluorescent proteins with a fluorescently labeled HaloTag. These FRET pairs enabled the straightforward design of biosensors for calcium, ATP and NAD^+^ with unprecedented dynamic ranges. The color of each of these biosensors can be readily tuned by changing either the fluorescent protein or the synthetic fluorophore, which enables simultaneous monitoring of free NAD^+^ in different subcellular compartments following genotoxic stress. Minimal modifications of these biosensors furthermore allow their readout to be switched to fluorescence intensity, fluorescence lifetime or bioluminescence. These FRET pairs thus establish a new concept for the development of highly sensitive and tunable biosensors.

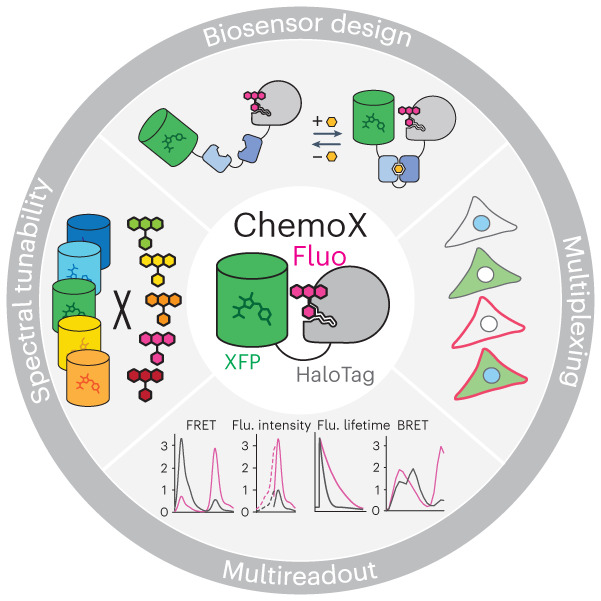

## Abstract (web summary)

Fluorescent proteins and HaloTag allow the flexible design of FRET-based biosensors with adjustable color using different fluorescent proteins or fluorophores and readout can be modified to fluorescence intensity, lifetime or bioluminescence.

## Main

Fluorescent biosensors are powerful tools for the investigation of cellular processes and allow for the real-time monitoring of biological activities, such as changes in metabolite concentration, with subcellular resolution^1^. Biosensors generally consist of two domains: one capable of sensing a biological activity or analyte and one translating it into a measurable readout. The readout of current fluorescent biosensors is mostly based on fluorescent proteins (FPs). Biosensors are engineered either by rendering the fluorescence intensity of the FP dependent on the presence of a biological activity^2^ or by exploiting the Förster resonance energy transfer (FRET) between two spectrally compatible FPs^1^. In both cases, engineering sensors with large changes in their spectral properties (that is, dynamic range) in response to changes in the biological activity of interest often requires laborious optimization and screening a large number of variants^2–5^. Intensiometric sensors often exhibit large dynamic ranges and require only a single emission channel. However, such sensors tend to be environmentally sensitive because their intensity change is often based on altering the protonation state of their chromophore^2^.

One popular method to develop FRET biosensors is to sandwich a sensing domain between a cyan FP (CFP) and yellow FP (YFP). These FPs exhibit a large spectral overlap that favor efficient FRET but result in spectral cross-talk, which limits their dynamic range while occupying a large part of the visible spectrum. To increase their dynamic range and spectral compatibility with other fluorescent tools, sensors based on green FP/red FP (GFP/RFP) or orange FP/RFP (OFP/RFP) FRET pairs have been developed, but their low FRET efficiencies also result in relatively small dynamic ranges^6–9^. In comparison to FPs, synthetic fluorophores exhibit overall superior photophysical properties, in particular for red-shifted wavelengths^10^. Rhodamines represent the largest class of synthetic fluorophores whose properties were highly optimized for molecular brightness, photostability and fluorogenicity, covering the visible and near-infrared spectrum^10^. On the other hand, self-labeling proteins (SLPs) enable specific labeling in live cells using cell-permeable bioorthogonal fluorophore substrates in an analogous manner to FPs^11^. SLPs in combination with rhodamines therefore represent appealing candidates as FRET pairs in biosensor design^12^. However, the sole implementation of synthetic fluorophores into the design of biosensors does not give access to large dynamic ranges. In a recent example, the CFP/YFP FRET pair of multiple biosensors was replaced by two SLPs labeled with different rhodamine fluorophores but reached relatively low dynamic ranges^13^.

We hypothesized that engineering a reversible interaction between a FP and fluorescently labeled SLP would enable the straightforward development of FRET biosensors with large dynamic ranges. We thus engineered an interface between a FP FRET donor and a rhodamine-labeled HaloTag^14,15^ FRET acceptor to reach near-quantitative FRET efficiency. By implementing our chemogenetic FRET pairs into the design of biosensors and fine-tuning the FP–HaloTag interface, we developed FRET biosensors for calcium, ATP and NAD^+^ with unprecedented dynamic ranges in a straightforward manner. The spectral tunability provided by the HaloTag labeling and by the common β-barrel architecture of FPs further enabled to readily choose the spectral properties of the sensors such that they could be multiplexed in fluorescence microscopy. Finally, we completed this chemogenetic toolbox by providing simple means to convert FRET biosensors into intensiometric and fluorescence lifetime-based sensors in the far-red range as well as bioluminescent sensors.

## Results

### Chemogenetic Förster resonance energy transfer pair engineering

**Fig. 1:**
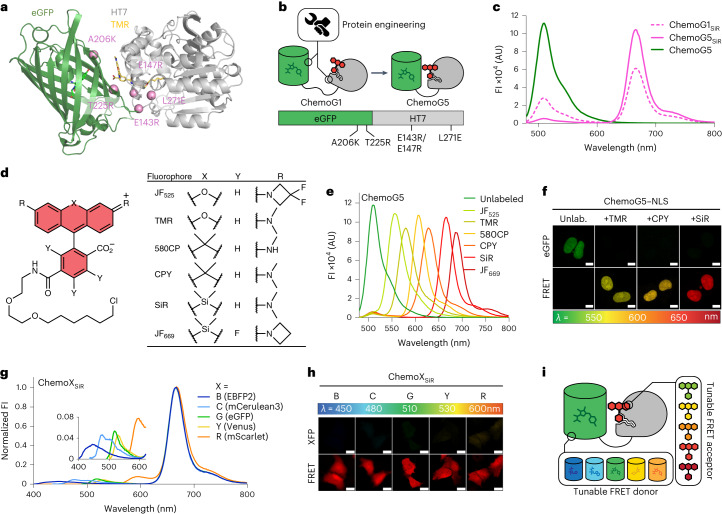
Development of chemogenetic FRET pairs with tunable wavelengths (ChemoX). **a**, Crystal structure of TMR-labeled ChemoG1; ChemoG1, eGFP–HT7 fusion construct; PDB ID: 8B6S, resolution of 1.8 Å. Proteins are represented as cartoons, and the eGFP chromophore and TMR are shown as sticks. Pink spheres represent the engineered positions at the eGFP and HT7 interface. **b**, Schematic representation of ChemoG1 interface engineering. **c**, Fluorescence intensity (FI) emission spectra of SiR-labeled ChemoG1 (ChemoG1_SiR_) and ChemoG5 (ChemoG5_SiR_) with unlabeled ChemoG5. Means of three technical replicates are shown; AU, arbitrary units. **d**, General chemical structure of rhodamine fluorophores. **e**, Fluorescence intensity emission spectra of ChemoG5 labeled with spectrally distinct rhodamine fluorophores listed in **d**. Means of three technical replicates are shown. **f**, Confocal images of U-2 OS cells expressing ChemoG5 in the nucleus (ChemoG5–NLS) labeled with TMR, CPY or SiR or unlabeled (Unlab.). Shown are the eGFP and FRET channels corresponding to the maximal emission of the respective fluorophores. Look up tables (LUT) of eGFP and FRET channels are adjusted to the same values for each condition; scale bars, 10 µm. **g**, Fluorescence intensity emission spectra of ChemoX constructs labeled with SiR. Spectra were normalized to the maximum FRET emission. The inset shows a zoom-in of the FRET donor fluorescence emission. Means of three technical replicates are shown. **h**, Confocal images of ChemoX constructs expressed in U-2 OS cells and labeled with SiR. Shown are the corresponding FP and FRET emission channels. LUT of XFP and FRET channels are adjusted to the same values for each construct; scale bars, 25 µm. **i**, Schematic representation of the spectral tunability of the ChemoX approach. Source data

**Extended Data Fig. 1:**
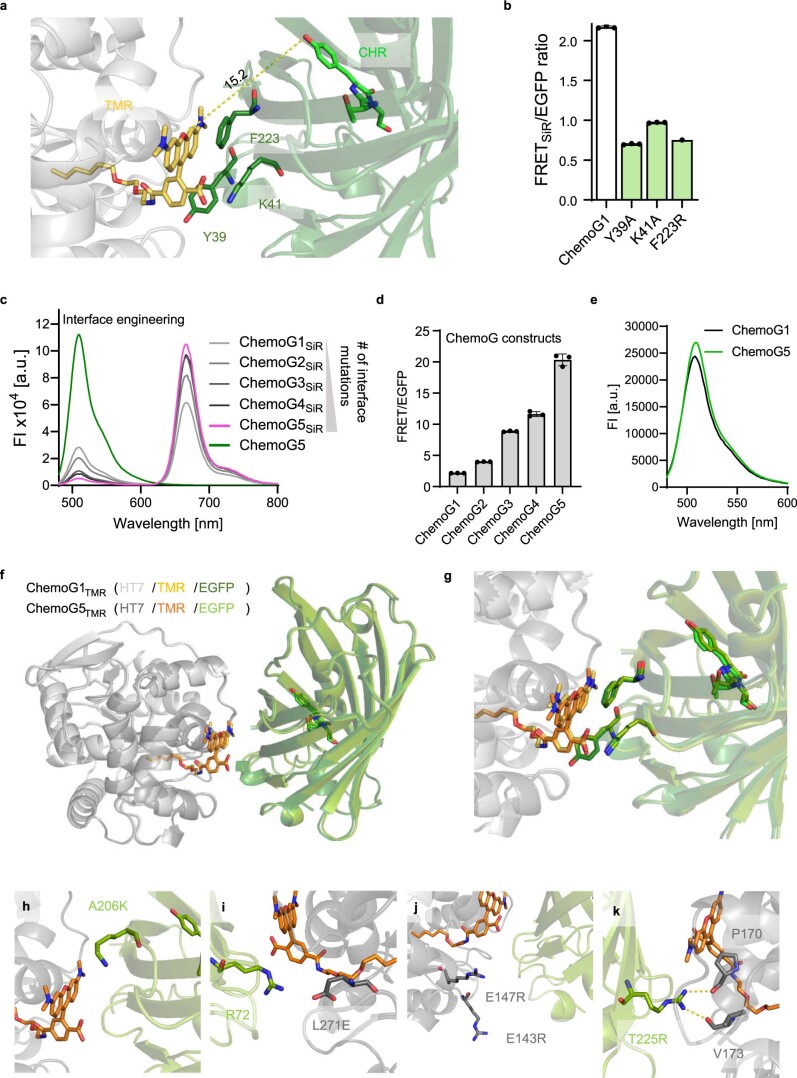
ChemoG-FRET surface engineering. **a**. X-ray structure of ChemoG1 labeled with TMR (PDB ID: 8B6S). Shown is the interface between eGFP and TMR labeled to HT. The eGFP chromophore and TMR are shown as sticks and their distance is marked with a dotted line. Residues Y39, K41 and F223 of eGFP involved in the direct interaction with TMR are annotated and shown as sticks. **b**. FRET ratios of ChemoG1 and variants carrying mutations of residues involved in the eGFP/HT7_TMR_ interface (n = 3 technical replicates, shown is the mean ±s.d.). **c**. Fluorescence intensity (FI) emission spectra of SiR-labeled ChemoG1-ChemoG5 and unlabeled ChemoG5. Represented are the means of 3 technical replicates. **d**. FRET ratios of SiR-labeled ChemoG1-ChemoG5 (n = 3 technical replicates, shown is the mean ±s.d.). **e**. Fluorescence intensity (FI) emission spectra of 100 nM unlabeled ChemoG1 and ChemoG5. Represented are the means of 3 technical replicates. **f**, **g**. Structural comparison between ChemoG1_TMR_ (PDB ID: 8B6S) and ChemoG5_TMR_ (PDB ID: 8B6T). Structures represented as in **a**. Overview (**f**) and zoom-in (**g**) of the interface between eGFP and TMR are shown. **h**-**k**. Zoom-ins of the ChemoG5_TMR_ X-ray structure showing the interface between eGFP and HT7 for each interface mutation (eGFP^A206K^, HT7^L271E^, HT7^E143R-E147R^ and eGFP^T225R^). Source data

To test the FRET between enhanced GFP (eGFP) and fluorescently labeled HaloTag7 (HT7), eGFP was fused directly to the N or C terminus of HT7 and labeled with the far-red fluorophore silicon rhodamine (SiR), and the fluorescence emission profile was measured to evaluate the intramolecular FRET efficiency of the chemogenetic design (Supplementary Fig. 1a). Fusing eGFP to the N terminus of HT7 (eGFP–HT7) revealed the highest FRET ratio (2.2 ± 0.1 (mean ± s.d.); Supplementary Fig. 1b). Because of its chemogenetic nature and involvement of eGFP, this design was named ChemoG1. As eGFP and SiR show only limited spectral overlap (Supplementary Fig. 1c), this high FRET ratio suggested that the two chromophores are in very close proximity. The X-ray structure of ChemoG1 labeled with the fluorophore tetramethylrhodamine (TMR; structurally similar to SiR; Protein Data Bank (PDB) ID: 8B6S, resolution of 1.8 Å) confirmed the fluorophore location at the interface between HT7 and eGFP, in close proximity to the eGFP chromophore (distance of 15.2 Å; Fig. 1a and Extended Data Fig. 1a). The eGFP surface residues Y39, K41 and F223 form a salt bridge with the carboxylate of the fluorophore (K41) and π-stacking interactions with the fluorophore benzyl (Y39) and xanthene (F223) moieties (Extended Data Fig. 1a). Their modification led to drastic loss in FRET (Extended Data Fig. 1b).

We hypothesized that the ChemoG1 conformation observed in the crystal structure exists only transiently in solution and identified interface mutations to stabilize it (that is, eGFP: A206K and T225R; HT7: E143R, E147R and L271E; Fig. 1b). These mutations improved the FRET efficiency compared to ChemoG1 in a stepwise manner, generating ChemoG2 to ChemoG5 (Extended Data Fig. 1c,d and Supplementary Table 1). SiR-labeled ChemoG5 (that is, ChemoG5_SiR_) carries all interface mutations and exhibits a near-quantitative FRET efficiency (95.8 ± 0.1%; Fig. 1c). The fluorescence intensity of eGFP was not affected by the surface mutations (Extended Data Fig. 1e). The X-ray structure of ChemoG5_TMR_ (PDB ID: 8B6T, resolution of 2.0 Å) revealed additional hydrogen bonds (T225R^eGFP^–P174/V177^HT7^) and electrostatic surface modifications (A206K^eGFP^, E143R–E147R^HT7^, L271E^HT7^–R72^eGFP^), likely responsible for the near-quantitative FRET (Extended Data Fig. 1f–k). Despite the ionic nature of some of the interactions, the FRET was minimally affected by changes in pH or salt concentration (Supplementary Fig. 2). In U-2 OS cells, the stepwise improvement in FRET of SiR-labeled ChemoG1 to ChemoG5 was confirmed by fluorescence microscopy with a maximum FRET/eGFP ratio of 16.4 ± 2.7 for ChemoG5 that, in turn, was not noticeably affected by changes in the local environment after expression in different subcellular locations (Supplementary Fig. 3 and Supplementary Table 2).

HT7 enables tuning of the spectral properties of the FRET acceptor on demand using different fluorophore substrates. Labeling ChemoG5 with different rhodamine fluorophores (Fig. 1d) yielded efficient FRET pairs with acceptor maximum fluorescence emission wavelengths ranging from 556 nm (Janelia Fluor_525_ (JF_525_)) to 686 nm (JF_669_; FRET efficiencies of ≥94%; Fig. 1e and Supplementary Table 3). By expressing ChemoG5 in the nucleus of U-2 OS cells (ChemoG5–nuclear localization signal (ChemoG5–NLS)), it was possible to confirm the spectral tunability of the FRET acceptor by fluorescence microscopy after labeling with different rhodamines (Fig. 1f). Labeling ChemoG5 with the structurally distinct cyanine fluorophores Cy3 or Cy5, however, resulted in lower FRET than the spectrally similar rhodamine fluorophores TMR and SiR, respectively (Supplementary Fig. 4a,b). The X-ray structure of Cy3-labeled HT7 (PDB ID: 8B6R, resolution of 1.5 Å; Supplementary Fig. 4c,d) revealed a Cy3 conformation at the HT7 surface incompatible with the interactions observed between TMR and eGFP in ChemoG5_TMR_, potentially explaining the weaker FRET.

**Extended Data Fig. 2:**
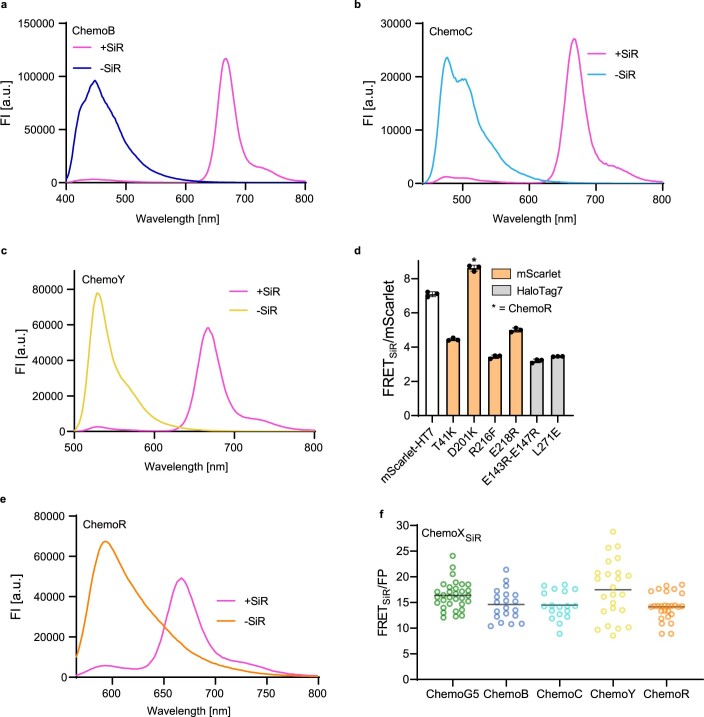
Expansion of ChemoG to other donor FPs. **a**-**c**, **e**. Fluorescence intensity (FI) emission spectra of optimized ChemoB (**a**), ChemoC (**b**), ChemoY (**c**) or ChemoR (**e**) labeled with SiR (+SiR) or unlabeled (-SiR). Interface mutations, FRET ratios and FRET efficiencies are listed in Supplementary Table S4. Shown are the means of 3 technical replicates. **d**. FRET/mScarlet ratios of SiR-labeled mScarlet-HT7 and mScarlet-HT7 variants with different mutations on mScarlet (orange) or HT7 (grey). The mutation D201K used in the optimized ChemoR construct is marked with an asterisk. Shown are the means ±s.d. (n = 3 technical replicates) **f**. FRET ratios of ChemoX constructs expressed in U-2 OS cells and labeled with SiR (*n* > 17 cells). Plotted for each construct are the FRET/FP ratios of individual cells (circles) and the mean (black line). The values are derived from 2 independent experiments. Source data

To further expand the spectral tunability of the ChemoG design, eGFP was exchanged with a blue (eBFP2), cyan (mCerulean3), yellow (Venus) or red (mScarlet) FP, creating the chemogenetic FRET constructs ChemoB, ChemoC, ChemoY and ChemoR, respectively. The design is thus named ChemoX, where ‘X’ refers to the color of the respective FP. Transposing the structural features of ChemoG5 to the ChemoX constructs led to optimized ChemoB, ChemoC and ChemoY variants all exhibiting near-quantitative FRET efficiencies (≥94%) after SiR labeling (Extended Data Fig. 2a–c and Supplementary Table 4). Attempts to transpose the structural features of ChemoG5 to ChemoR proved challenging (Extended Data Fig. 2d), probably because of the different phylogenetic origin of mScarlet^16^. Favored by the large spectral overlap between mScarlet and SiR, the initial ChemoR construct nevertheless showed a high FRET efficiency that was further increased by the mutation D201K^mSca^ (91.3 ± 0.3%; Extended Data Fig. 2d,e and Supplementary Table 4). The ChemoX palette offers multiple options throughout the visible spectrum (Fig. 1g) and displays efficient FRET in cells (FRET ratio of >14) such that the fluorescence emission was almost exclusively observed in the FRET channel (Fig. 1h and Extended Data Fig. 2f). ChemoX thus constitutes a platform of FRET pairs whose colors can be readily chosen by exchanging the FP or the synthetic fluorophore (Fig. 1i).

### ChemoX-based calcium Förster resonance energy transfer sensors

**Fig. 2:**
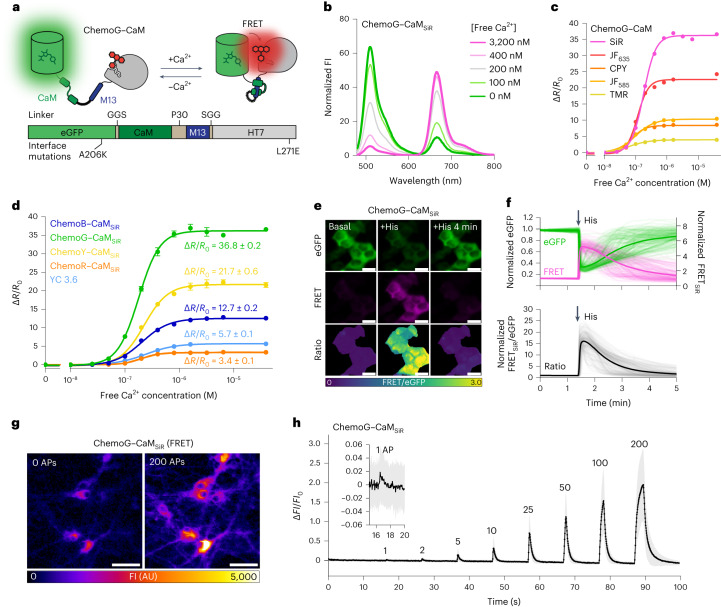
Development of ratiometric calcium sensors based on ChemoX. **a**, Schematic representation of ChemoG–CaM. **b**, Normalized fluorescence intensity emission spectra of SiR-labeled ChemoG–CaM at different concentrations of free calcium. Means of three technical replicates are shown; [Free Ca^2+^], concentration of free calcium. **c**, Calcium titration curves of ChemoG–CaM labeled with different fluorophores. Data are shown as the mean ± s.d. of the FRET/eGFP ratio changes (Δ*R*/*R*_0_; *n* = 3 technical replicates). Δ*R*/*R*_0_ and C_50_ data are summarized in Supplementary Table 6. **d**, Calcium titration curves of ChemoX–CaM_SiR_ and YC 3.6. Data are shown as in **c** (*n* = 3 technical replicates). Δ*R*/*R* values are indicated and summarized together with the C_50_ data in Supplementary Table 5. **e**, Widefield images of HeLa Kyoto cells transiently expressing ChemoG–CaM labeled with SiR. Shown are the eGFP channel, the FRET channel and the ratio image of both channels (FRET/eGFP) in pseudocolor (LUT = mpl-viridis). The images represent cells under basal conditions before the addition of histamine (basal), 15 s after the addition of histamine (+His) and 4 min after the addition of histamine (+His 4 min); scale bars, 25 µm. **f**, Time course measurement of ChemoG–CaM_SiR_ fluorescence intensity in HeLa Kyoto cells. Represented are the eGFP and FRET channel (top) and FRET/eGFP ratio normalized to 1 at *t* = 0 min (bottom). Cells were treated with histamine at the time point indicated with an arrow; *n* = 161 cells from three biological replicates. Represented are the means (solid line) plus traces of individual cells (dim lines). **g**, Widefield images of rat hippocampal neurons expressing cytosolic ChemoG–CaM labeled with SiR at different stimulation intensities. Neurons were stimulated with an electric field corresponding to 0 or 200 APs. The fluorescence intensity of the SiR FRET channel is represented in pseudocolor (LUT = Fire); scale bars, 50 µm. **h**, Time course measurement of ChemoG–CaM_SiR_ fluorescence intensity in rat hippocampal neurons. Represented is the FRET fluorescence intensity change (Δ*FI*/*FI*_0_) after electric field stimulation; *n* = 61 cells from three biological replicates. The numbers of APs are indicated. Data are shown as mean (line) ± s.d. (shaded area). Source data

**Extended Data Fig. 3:**
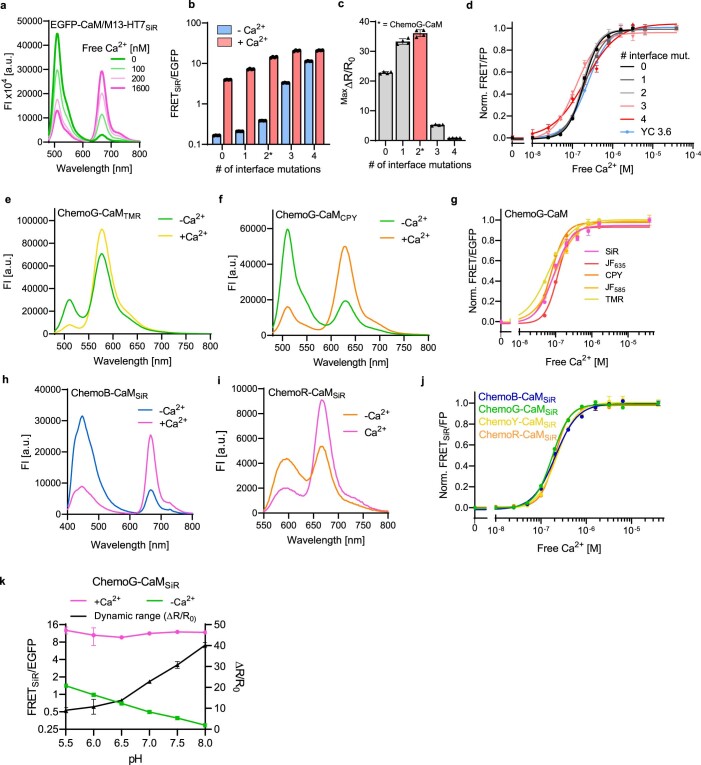
Engineering and characterization of ChemoX-CaM calcium sensors. **a**. Fluorescence intensity (FI) emission spectra of eGFP-CaM/M13-HT7 labeled with SiR in presence of different concentrations of free Ca^2+^. Shown are the means of 3 technical replicates. **b**. FRET/eGFP ratios of calcium sensors differing in the number of interface mutations (Supplementary Table S5) in presence (39 μM) or absence (0 μM) of free Ca^2+^. Sensors were labeled with SiR. The variant corresponding to the final calcium sensor ChemoG-CaM is marked with an asterisk. Shown are the means ±s.d. *n* = 4 technical replicates. **c**. Maximal FRET/eGFP ratio changes (^Max^ΔR/R_0_) of calcium sensors differing in the number of interface mutations (Supplementary Table S5). Sensors were labeled with SiR. The variant corresponding to the final calcium sensor ChemoG-CaM is marked in red and with an asterisk. Shown are the means ±s.d. *n* = 4 technical replicates. **d**, **g**, **j**. Ca^2+^ titrations of SiR-labeled calcium sensors differing in the number of interface mutations (**d**, Supplementary Table S5), ChemoG-CaM labeled with different fluorophores (**g**, Supplementary Table S6) or ChemoX-CaM calcium sensors labeled with SiR (**j**, Supplementary Table S5). Shown are the means ±s.d. *n* = 3 technical replicates. **e**, **f**. Fluorescence intensity (FI) emission spectra of ChemoG-CaM labeled with TMR (**e**) or CPY (**f**) in presence (39 μM) or absence (0 μM) of free Ca^2+^. Shown are the means of 3 technical replicates. **h**, **i**. Fluorescence intensity (FI) emission spectra of ChemoB-CaM (**h**) or ChemoR-CaM (**i**) labeled with SiR in presence (39 μM) or absence (0 μM) of free Ca^2+^. Shown are the means of 3 technical replicates. **k**. pH sensitivity of the FRET/eGFP ratio of ChemoG-CaM_SiR_ in presence (2 mM CaCl_2_, +Ca^2+^) or absence (2 mM EGTA, -Ca^2+^) of free Ca^2+^. Shown are the means ±s.d of three technical replicates. Source data

We hypothesized that a reversible interaction of FPs with the fluorescently labeled HT7 in ChemoX could enable the development of a new family of fluorescent biosensors. As initial proof of principle and analogously to the calcium sensor yellow cameleon 3.6 (YC 3.6)^17^, we designed a calcium sensor in which eGFP and HT7 sandwich the calcium-binding protein calmodulin (CaM) and its cognate binding peptide M13 (eGFP–CaM/M13–HT7; Fig. 2a). CaM and M13 were connected via a polyproline linker (P30), while short GGS linkers connected the FP and HT7 with the sensing domains. The SiR-labeled construct eGFP–CaM/M13–HT7 displayed a large change of fluorescence emission spectrum with increasing concentrations of free calcium (Extended Data Fig. 3a), displaying a maximal FRET/eGFP ratio (*R*) change (^max^Δ*R*/*R*_0_) of 22.8 ± 0.3, also referred to as dynamic range. The introduction of the previously identified eGFP–HT7 interface mutations (Supplementary Table 1) into the sensor led to a stepwise FRET increase both in the absence and presence of free calcium (Extended Data Fig. 3b). This approach revealed key in tuning the dynamic range of the sensor (Extended Data Fig. 3c), allowing us to identify ChemoG–CaM, a calcium sensor with the interface mutations A206K^eGFP^ and L271E^HT7^. ChemoG–CaM_SiR_ showed a large dynamic range (^max^Δ*R*/*R*_0_ = 36.1 ± 1.0-fold; Fig. 2b and Supplementary Table 5) with a half-maximal response concentration (C_50_) of 179 nM free calcium (95% confidence interval (95% CI): 173–185 nM), similar to YC 3.6 (243 nM; 95% CI: 233–251 nM). Furthermore, the C_50_ of the sensors was independent of the number of interface mutations (Extended Data Fig. 3d and Supplementary Table 5). The ChemoX–CaM sensor color can be readily tuned by either changing the synthetic fluorophore or the FP. Using mRuby2 instead of mScarlet led to a sensor with increased dynamic range (^max^Δ*R*/*R*_0_ = 3.4 ± 0.1-fold) and was named ChemoR–CaM_SiR_ (Supplementary Fig. 5 and Supplementary Table 5). All generated sensors exhibited large dynamic ranges, outperforming YC 3.6 with the exception of the red version of the sensor (Fig. 2c,d, Extended Data Fig. 3e–j and Supplementary Tables 5 and 6). From here, ChemoR biosensors will always be based on mRuby2, as it proved more potent in yielding biosensors with larger dynamic ranges. The SiR-labeled mRuby2–HT7 fusion (ChemoRuby2) showed a FRET efficiency (91.7 ± 0.2%; Supplementary Fig. 5g) comparable to the mScarlet–HT7 fusion (Supplementary Table 4). We also explored the possibility of using rhodamine-labeled HT7 as the FRET donor and FP as the FRET acceptor by labeling ChemoR–CaM with JF_525_. However, this strategy resulted in a sensor with limited performance (^max^Δ*R*/*R*_0_ = 55.5 ± 2.7%; Supplementary Fig. 5h). All sensors present similar C_50_ values, illustrating the feasibility of exchanging FRET donor and acceptor with minimal impact (Extended Data Fig. 3g,j). Subtle differences in the behavior of the sensors could nevertheless be observed, such as the sensors’ Hill slopes (Supplementary Tables 5 and 6). As previously described for YC 3.6 (ref. ^17^), ChemoG–CaM_SiR_ was sensitive to pH changes (Extended Data Fig. 3k). This sensitivity might come from the sensing domain because ChemoG1–ChemoG5 were found to be mostly pH insensitive over the same range (Supplementary Fig. 2).

**Extended Data Fig. 4:**
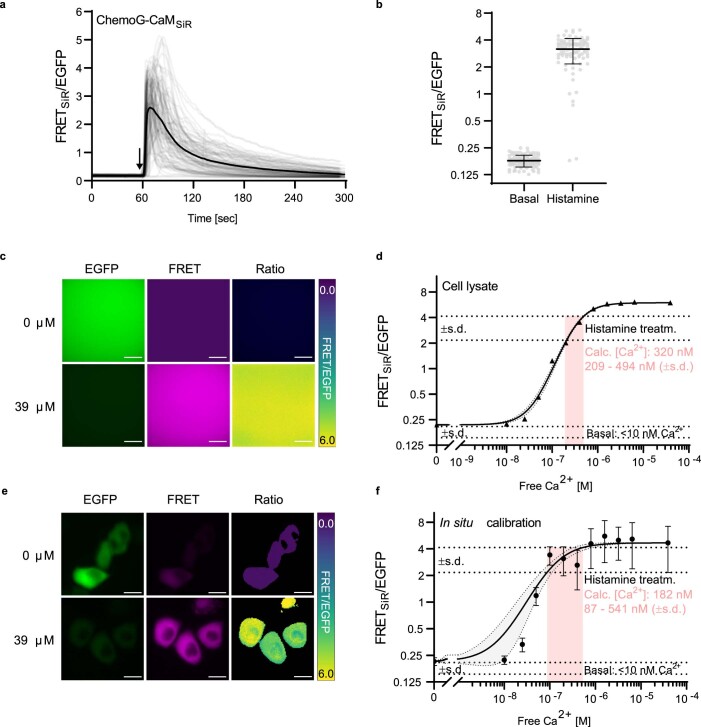
Absolute quantification of cytosolic calcium concentrations using ChemoG-CaM_SiR_. **a**. Time course measurement of free intracellular calcium fluctuations using ChemoG-CaM_SiR_ in HeLa Kyoto cells. Represented are the FRET_SiR_/eGFP ratios. Cells were imaged in HBSS and treated with histamine (indicated with an arrow; *n* = 96 cells from 3 biological replicates). **b**. Comparison of the FRET_SiR_/eGFP ratios of ChemoG-CaM_SiR_ measured in HeLa Kyoto cells in resting conditions (basal) and upon treatment with histamine. Data extracted from experiment described in panel **a**. Shown are the means ±s.d. **c**. Widefield microscopy images of lysate from HeLa Kyoto cells expressing SiR-labeled ChemoG-CaM. Lysate was equilibrated in calcium calibration buffers containing 0 μM or 39 μM free calcium. Shown are the eGFP channel, the FRET_SiR_ channel and the ratio image of both channels (FRET_SiR_/eGFP) in pseudocolor (LUT = mpl-viridis). **d**. ChemoG-CaM_SiR_ calcium titration curve obtained from HeLa Kyoto cell lysate. Shown are the means ±s.d. of n = 3 technical replicates. The mean calcium concentrations (±s.d.) in histamine stimulated states were calculated from the FRET_SiR_/eGFP ratios measured in HeLa Kyoto cells (from panels **a**-**b**). The 95% confidence interval of the non-linear regression fit is shown as grey shade. **e**. Widefield images of HeLa Kyoto cells transiently expressing ChemoG-CaM labeled with SiR. Cells were permeabilized and equilibrated in calcium calibration buffers containing 0 μM or 39 μM free calcium. Shown are the eGFP channel, the FRET_SiR_ channel and the ratio image of both channels (FRET_SiR_/eGFP) in pseudocolor (LUT = mpl-viridis). **f**. ChemoG-CaM_SiR_ calcium titration curve obtained from permeabilized HeLa Kyoto cells. Shown are the means ±s.d. of *n* = 2 biological replicates. The mean calcium concentrations (±s.d.) in histamine stimulated states were calculated from the FRET_SiR_/eGFP ratios measured in HeLa Kyoto cells (from panels **a**-**b**). The 95% confidence interval of the non-linear regression fit is shown as grey shade. Calcium resting concentrations in cells (basal) could not be determined since the FRET_SiR_/eGFP ratios were below the lower measured ratio in sensor calibration and was hence interpreted as below the lowest calcium concentration used in calibration *that is* < 10 nM. Source data

Next, we confirmed the performance of ChemoG–CaM_SiR_ in HeLa cells, where the sensor showed a large FRET increase (∆*R*/*R*_0_ = 16.8 ± 3.9-fold) after histamine-induced calcium influx into the cytosol (Fig. 2e,f). We noticed that overexpression of CaM-based calcium sensors like ChemoX–CaM or YC 3.6 resulted in reduced calcium oscillations after treatment with histamine (Supplementary Fig. 6), an artifact commonly described in the literature and attributed to the calcium buffering effect^18,19^. The ratiometric readout of ChemoG–CaM_SiR_ enabled the observation that the cytosolic calcium concentration in HeLa cells reaches an average of 320 nM (±s.d. of 209–494 nM) after treatment with histamine (10 μM; Extended Data Fig. 4), which is in line with values described in the literature^20^. Accurately determining absolute concentrations in live cells using biosensors remains a challenge due to the difficulty of establishing a reliable calibration curve in a cell-like environment. As a result, interpreting values obtained through this approach requires caution. Finally, considering the importance of calcium transients in neurobiology, adeno-associated virus (AAV)-delivered ChemoG–CaM_SiR_ was characterized in rat primary hippocampal neurons. The sensor displayed a maximum FRET_SiR_ increase (fluorescence intensity change (∆*FI*/*FI*_0_)) of 206 ± 90% after electric field stimulation (200 action potentials (APs)) and was able to detect as low as 1 AP (Fig. 2g,h). While ChemoG–CaM_SiR_ is inferior to the GCamP8 series^21^, it’s maximum ∆*FI*/*FI*_0_ is comparable to the recently developed HaloCaMP1a and HaloCaMP1b in combination with JF_635_ (ref. ^22^).

### ChemoX-based ATP FRET sensors

**Fig. 3:**
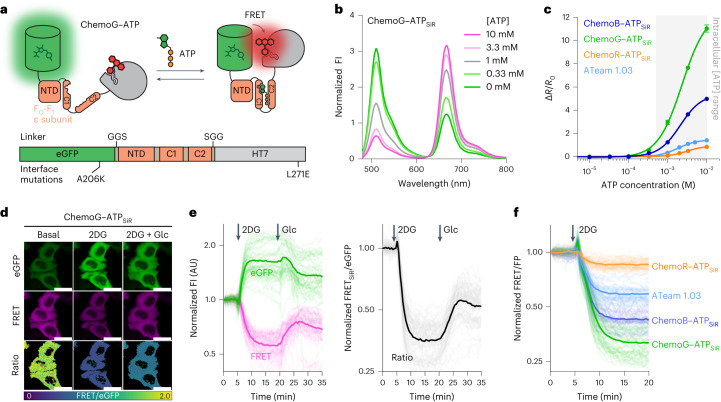
Development of ratiometric ATP sensors based on ChemoX. **a**, Schematic representation of ChemoG–ATP; NTD, N-terminal domain. **b**, Fluorescence intensity emission spectra of SiR-labeled ChemoG–ATP at different ATP concentrations. Means of three technical replicates are shown; [ATP], ATP concentration. **c**, ATP titration curves of ChemoX–ATP_SiR_ sensors. Data are shown as the means ± s.d. of the FRET/eGFP ratio changes (Δ*R*/*R*_0_; *n* = 3 technical replicates). The intracellular ATP concentration range is indicated with a gray box. Δ*R*/*R*_0_ and C_50_ values are summarized in Supplementary Table 7. **d**, Confocal images of HeLa Kyoto cells expressing ChemoG–ATP labeled with SiR. Shown are the eGFP channel, the FRET channel and the ratio image of both channels (FRET/eGFP) in pseudocolor (LUT = mpl-viridis). Cells were treated at *t* = 5 min with 10 mM 2DG. At *t* = 20 min, 20 mM glucose (Glc) was added to the cells until the end of the experiment (*t* = 35 min, 2DG + Glc); scale bars, 25 µm. **e**, Time course measurement of ChemoG–ATP_SiR_ fluorescence intensity in HeLa Kyoto cells. Shown are the eGFP and FRET channels (left) and FRET/eGFP ratio (right) normalized to 1 at *t* = 0 min. Cells were treated with 10 mM 2DG and subsequently with 20 mM glucose at time points indicated with arrows. Experiments are as explained in **d**; *n* = 59 cells from three biological replicates. Represented are the means (solid lines) and traces of the individual cells (dim lines). **f**, Time course measurement of ChemoB–ATP_SiR_ (*n* = 58 cells), ChemoG–ATP_SiR_ (*n* = 63 cells), ChemoR–ATP_SiR_ (*n* = 52 cells) and ATeam 1.03 (*n* = 59 cells) fluorescence intensity in HeLa Kyoto cells. The FRET/FP ratio after treatment with 10 mM 2DG is shown. Ratios are normalized to 1 at *t* = 0 min. Addition of 2DG is indicated with an arrow. Represented are the means (line) and single-cell traces (dim lines) from three biological replicates. Source data

**Extended Data Fig. 5:**
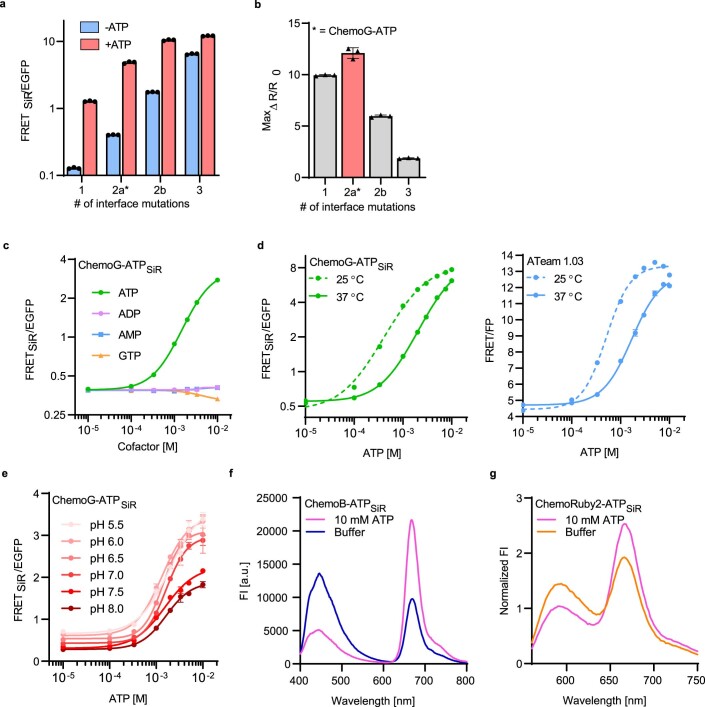
Engineering and characterization of ChemoX-ATP sensors. **a**. FRET/eGFP ratios of ATP sensors differing in the number of interface mutations (Supplementary Table S7) in presence (+ATP) or absence (+ATP) of 10 mM ATP. Asterisk indicates construct corresponding to the final sensor ChemoG-ATP. Shown are the means ±s.d. *n* = 3 technical replicates. **b**. Maximal FRET/eGFP ratio changes (^Max^ΔR/R_0_) of ATP sensors differing in the number of interface mutations (Supplementary Table S7). Asterisk indicates construct corresponding to the final sensor ChemoG-ATP. Shown are the means ±s.d. *n* = 3 technical replicates. **c**. Titrations of ChemoG-ATP_SiR_ with ATP and structurally related molecules. **d**. Titrations of ChemoG-ATP_SiR_ and ATeam 1.03 with ATP at different temperatures. **e**. Titrations of ChemoG-ATP_SiR_ and ATeam 1.03 with ATP at different pH. Shown are the means ±s.d. of 3 technical replicates. **f, g**. Fluorescence intensity (FI) emission spectra of ChemoB-ATP (**f**) and ChemoR-ATP (**g**) labeled with SiR (Supplementary Table S7) in presence or absence of 10 mM ATP. Shown are the means of 3 technical replicates. Source data

ATP is essential for cellular energy homeostasis^23^ and plays important roles in signaling processes^24^. However, biosensors currently available to investigate intracellular changes in free ATP show limited dynamic ranges and are spectrally restricted^25–27^. We therefore developed a ChemoG-based ATP biosensor by exchanging the FRET pair mseCFP and Venus of the ATP biosensor ATeam 1.03 (ref. ^25^) with eGFP and HT7, respectively (Fig. 3a). The dynamic range of the construct eGFP–F_O_-F_1_–HT7_SiR_ was optimized by the introduction of eGFP–HT7 interface mutations A206K^eGFP^ and L271E^HT7^, obtaining ChemoG–ATP_SiR_ with a ^max^Δ*R*/*R*_0_ of 12.1 ± 0.4-fold (Fig. 3b, Extended Data Fig. 5a,b and Supplementary Table 7). The sensor responds to millimolar concentrations of ATP (C_50_ = 2.33 mM; 95%CI: 2.18–2.53 mM) with high selectivity over other nucleotides (Extended Data Fig. 5c). Consistent with previous observations for ATeam 1.03 (ref. ^25^), the sensor is sensitive to changes in temperature and pH (Extended Data Fig. 5d,e). Based on the ChemoX design, a color palette of ATP sensors was developed (Extended Data Fig. 5f,g), which exhibits dynamic ranges larger or similar than those of ATeam 1.03 (Fig. 3c and Supplementary Table 7).

ChemoG–ATP_SiR_ was expressed in the cytosol of HeLa Kyoto cells where treatment with the glycolysis inhibitor 2-deoxy-d-glucose (2DG) led to a strong FRET/eGFP decrease (Δ*R*/*R*_0_ = −67.9 ± 6.3%) that could be partially compensated by perfusing high concentrations of glucose (Fig. 3d,e). The FRET/eGFP ratio briefly increased immediately after addition of 2DG, which we attribute to the transient change in temperature. Similarly, ChemoB–ATP_SiR_ and ChemoR–ATP_SiR_ reported a 2DG-induced decrease in cytosolic ATP (ChemoB–ATP_SiR_ Δ*R*/*R*_0_ = −57.5 ± 4.7%; ChemoR–ATP_SiR_ Δ*R*/*R*_0_ = −14.5 ± 4.9%), which we compared to ATeam 1.03 (Δ*R*/*R*_0_ = −41.1 ± 5.1%; Fig. 3f). While ChemoB–ATP_SiR_ was found to be more effective than ATeam 1.03 in translating intracellular ATP concentration changes into FRET changes, this was not the case for ChemoR–ATP_SiR_, consistent with in vitro titrations (Fig. 3c).

### ChemoX-based NAD^+^ FRET sensors

**Fig. 4:**
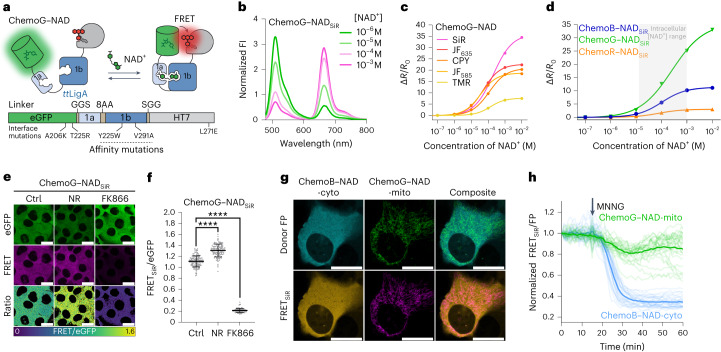
Multiplexing subcellular NAD^+^ pools using ChemoX–NAD biosensors. **a**, Schematic representation of ChemoG–NAD. **b**, Normalized fluorescence intensity emission spectra of SiR-labeled ChemoG–NAD at different NAD^+^ concentrations. Means of three technical replicates are shown; [NAD^+^], NAD^+^ concentration. **c**, NAD^+^ titration curves of ChemoG–NAD labeled with different fluorophores. Data are shown as the means ± s.d. of the FRET/eGFP ratio changes (Δ*R*/*R*_0_; *n* = 3 technical replicates). Δ*R*/*R*_0_ and C_50_ values are summarized in Supplementary Table 8. **d**, NAD^+^ titration curves of ChemoX–NAD_SiR_ biosensors. Data are shown as the means ± s.d. of the FRET/eGFP ratio changes (Δ*R*/*R*_0_; *n* = 3 technical replicates). The intracellular free NAD^+^ concentration range is indicated with a gray box. Δ*R*/*R*_0_ and C_50_ values are summarized in Supplementary Table 8. **e**, Confocal images of U-2 OS cells expressing ChemoG–NAD labeled with SiR. Shown are the eGFP channel, the FRET channel and the ratio image of both channels (FRET/eGFP) in pseudocolor (LUT = mpl-viridis). Cells were treated for 24 h with DMSO (Ctrl), 100 nM FK866 or 1 mM NR; scale bars, 25 µm. **f**, Dot plots representing the FRET/eGFP ratios of ChemoG–NAD_SiR_ expressed in U-2 OS cells treated as described in **d**; *n* = 133 (Ctrl), 117 (NR) and 132 (FK866) cells from three independent experiments. *P* values are given based on unpaired two-tailed *t*-test with Welch’s correction; ****P < 0.0001. Data are shown as the means ± s.d. **g**, Confocal image of U-2 OS cell coexpressing ChemoB–NAD-cyto and ChemoG–NAD-mito labeled with SiR. Shown are the FRET donor FP channels, the FRET channels and the composites of the FP or FRET channel of both sensors pseudocolored (eBFP2 (cyan), eGFP (green), eBFP2-FRET (orange) and eGFP-FRET (magenta)). The brightness of the donor and FRET channels was adjusted to show potential cross-talk between the channels; scale bars, 25 µm. **h**, Time course measurement of ChemoB–NAD-cyto (cytosol) and ChemoG–NAD-mito (mitochondria) fluorescence intensity coexpressed in U-2 OS cells and labeled with SiR. Represented are the means of the FRET/FP ratios (line) and single-cell traces (dim lines) normalized to 1 at *t* = 0 min. The addition of MNNG is indicated with an arrow (*n* = 28 cells from four biological replicates). Source data

**Extended Data Fig. 6:**
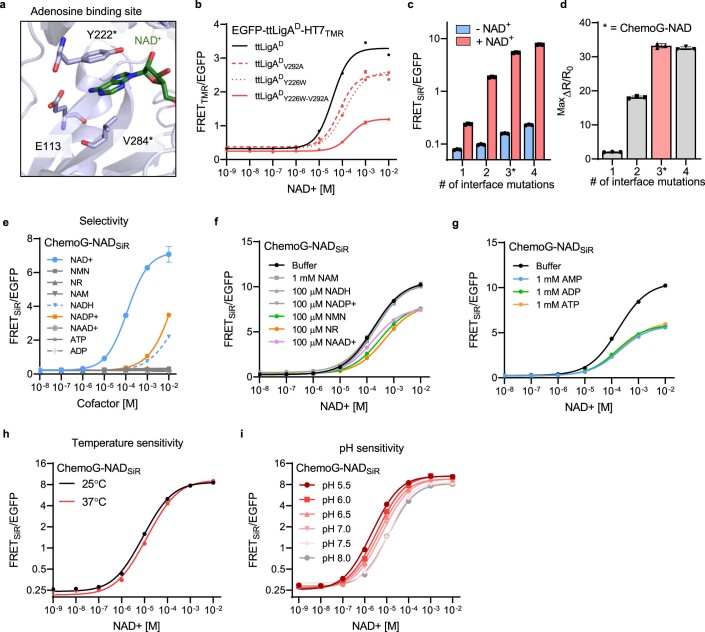
Engineering and characterization of ChemoX-NAD sensors. **a**. Zoom-in on the NAD^+^ binding site of the X-ray structure of LigA from *Enterococcus faecalis (ef*LigA*)* bound to NAD^+^ (PDB ID: 1TAE). The structure is represented as cartoon (light blue) and NAD^+^ (green) and residues involved in the binding of NAD^+^ (Y222 and V284, light blue) are represented as sticks. *Y222 and V284 of *ef*LigA correspond to Y226 and V292 of LigA from *Thermus thermophilus* (*tt*LigA). **b**. NAD^+^ titrations of sensor variants labeled with TMR. *tt*LigA^D^ carries the extra mutations K117L and D289N rendering it catalytically inactive. Mutations Y226W and Y292A shift the sensor response towards the range of free intracellular NAD^+^. **c**. FRET ratios of NAD^+^ sensors differing in the number of interface mutations (Supplementary Table S5) in presence (+NAD^+^) or absence (-NAD^+^) of 1 mM NAD^+^. **d**. Maximal FRET/eGFP ratio change (^Max^ΔR/R_0_) of NAD+ sensors differing in the number of interface mutations. Asterisk indicates construct corresponding to the final sensor ChemoG-NAD. **e**. Titration of ChemoG-NAD with NAD^+^ or structurally related molecules. **f**. Titration of ChemoG-NAD_SiR_ with NAD+ in presence of different structurally related molecules. **g**. Titration of ChemoG-NAD_SiR_ with NAD^+^ in presence of 1 mM AMP, ADP or ATP. **h**, **i**. NAD^+^ titrations of ChemoG-NAD_SiR_ at different temperatures (**h**) or at different pH (**i**). For all graphs, the means of 3 technical replicates ±s.d. are shown. Source data

NAD^+^ is highly compartmentalized within cells^28^, and the regulation of its subcellular pools plays an important role in many biological processes^29,30^. However, monitoring changes in intracellular NAD^+^ at multiple subcellular locations is limited by the spectral incompatibility and selectivity of current biosensors^31–33^. Inspired by the recently developed NAD^+^ sensor based on DNA ligase A (LigA)^32,34^, we developed a sensor based on the catalytically inactive LigA from *Thermus thermophilus* (*tt*LigA^K118L–D289N^ (*tt*LigA^D^)), sandwiching the sensing domain between eGFP and HT7 (Fig. 4a). The sensor’s C_50_ was optimized by implementing the mutations Y226W and V292A in *tt*LigA^D^ (Extended Data Fig. 6a,b), and the dynamic range was optimized by implementing selected eGFP–HT7 interface mutations (that is, A206K–T225R^eGFP^ and L271E^HT7;^ Extended Data Fig. 6c,d). The resulting ChemoG–NAD_SiR_ sensor presents a high dynamic range (^max^Δ*R*/*R*_0_ = 34.7 ± 0.4-fold) and a C_50_ (200 µM; 95% CI: 182–220 µM) in the range of expected intracellular NAD^+^ concentrations (50–400 µM depending on the compartment^31,32^; Fig. 4b and Supplementary Table 8). Furthermore, the sensor did not respond to increasing concentrations of NAD^+^ precursors or structurally related molecules alone (Extended Data Fig. 6e). However, NAD^+^ titrations in the presence of certain NAD^+^ precursors or adenine nucleotides did affect the FRET ratio of the sensor (Extended Data Fig. 6f,g). Because AMP, ADP and ATP affect the sensor response for NAD^+^ to the same extent, a relative change in the ATP:ADP:AMP ratio should not affect the response of the sensor in cells but only a change in the total concentration of the adenosine nucleotide pool. Finally, temperature and pH moderately influenced the sensor’s response (Extended Data Fig. 6h,i). The spectral properties of the sensor can be readily tuned based on the ChemoX design, yielding a color palette of NAD^+^ sensors including ChemoB–NAD_SiR_ (^max^Δ*R*/*R*_0_ = 11.2 ± 0.1-fold) and ChemoR–NAD_SiR_ (^max^Δ*R*/*R*_0_ = 3.0 ± 0.1-fold; Fig. 4c,d, Supplementary Fig. 7 and Supplementary Table 8).

**Extended Data Fig. 7:**
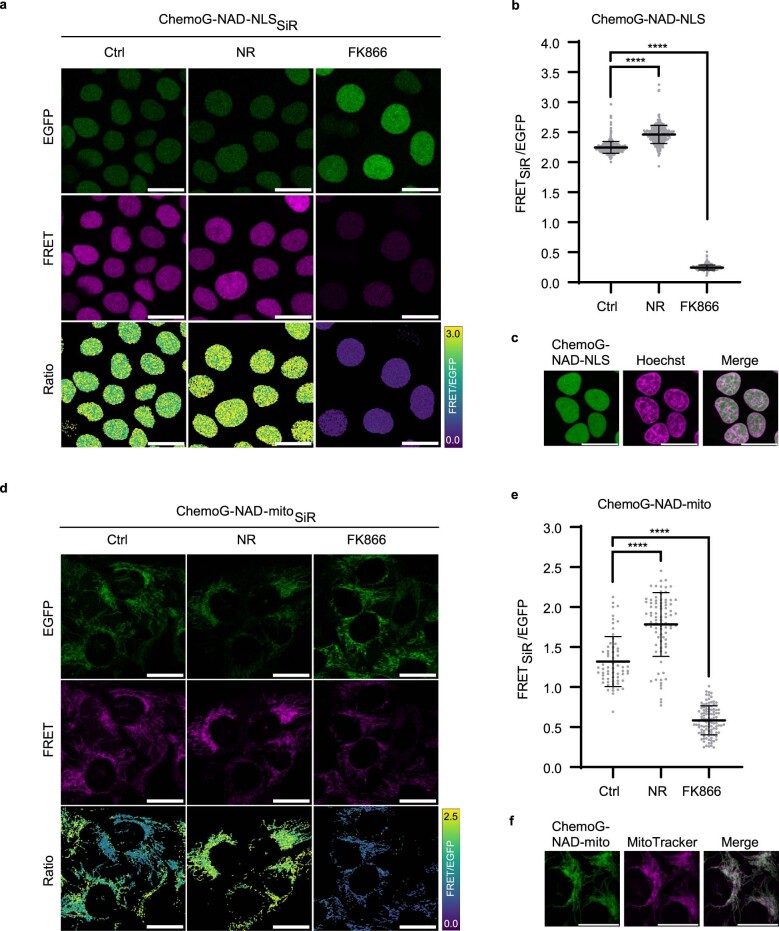
Performance of ChemoG-NAD in different subcellular compartments. **a**, **d**. Confocal images of U-2 OS cells expressing ChemoG-NAD in the nucleus (ChemoG-NAD-NLS, **a**) or mitochondria (ChemoG-NAD-mito, **d**) labeled with SiR. Shown are the eGFP channel, FRET channel and ratio image (FRET/eGFP) in pseudocolor (LUT = mpl-viridis). Cells were treated for 24 h either with DMSO (Ctrl), 100 nM FK866 or 1 mM NR. All scale bars = 25 μm. **b**, **e**. FRET/eGFP ratios of U-2 OS cells corresponding to panels **a** and **d**, respectively. Shown are the FRET/eGFP ratios of single cells (circles) and the mean ±s.d. (black line) (nucleus: n = 353 (ctrl), 311 (NR), 300 (FK866) cells; mitochondria: *n* = 63 (ctrl), 85 (NR), 109 (FK866) cells; from 3 independent experiments). p-values are given based on unpaired two-tailed t-test with Welch’s correction (**** p < 0.0001). **c**. Confocal images of U-2 OS cells expressing ChemoG-NAD in the nucleus (ChemoG-NAD-NLS) and stained with Hoechst. **f**. Confocal images of U-2 OS cells expressing ChemoG-NAD in the mitochondria (ChemoG-NAD-mito) and stained with MitoTracker RedFM. All scale bars = 25 μm. Source data

We then studied the impacts of the nicotinamide phosphoribosyltransferase inhibitor FK866 and the NAD^+^ precursor nicotinamide riboside (NR) on free NAD^+^ concentrations in U-2 OS cells using cytosolically expressed ChemoG–NAD_SiR_. In fluorescence microscopy experiments, the FK866-induced NAD^+^ depletion led to a strong FRET ratio decrease (Δ*R*/*R*_0_ = −78.9 ± 3.6%), whereas treatment with NR increased intracellular NAD^+^ as indicated by a substantial FRET ratio increase (Δ*R*/*R*_0_ = 28.4 ± 11.4%; Fig. 4e,f). The sensor was able to translate NAD^+^ changes with similar trends in the nuclei and mitochondria of U-2 OS cells (Extended Data Fig. 7). While ChemoB–NAD_SiR_ showed similar performances to ChemoG-NAD_SiR_ in cells (FK866: Δ*R*/*R*_0_ = −66.2 ± 8.5%; NR: Δ*R*/*R*_0_ = 17.3 ± 21.1%), ChemoR–NAD_SiR_ presented a reduced sensitivity (FK866: Δ*R*/*R*_0_ = −21.3 ± 3.2%; NR: Δ*R*/*R*_0_ = 2.5 ± 4.5%; Supplementary Fig. 8), which is nevertheless comparable to previously published NAD^+^ biosensors^31,32^ and of interest for multiplexing purposes.

**Extended Data Fig. 8:**
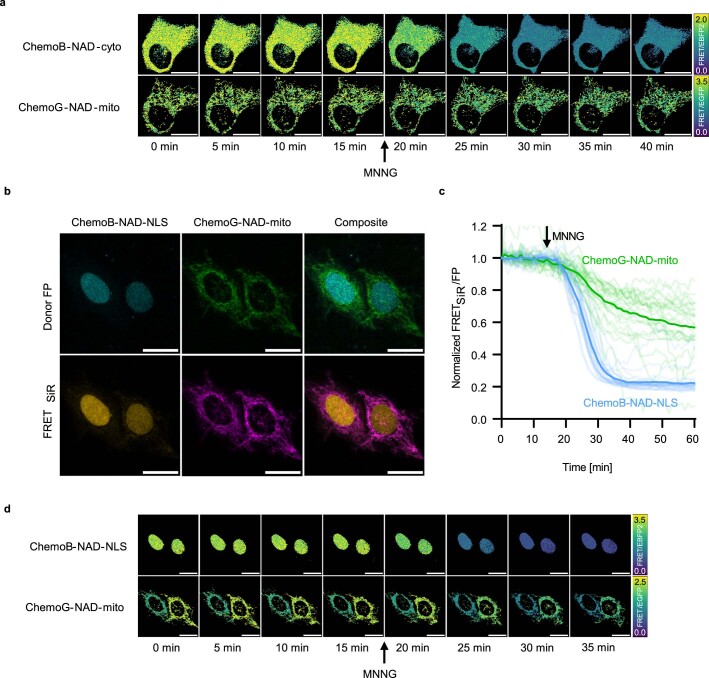
Multiplexed imaging of ChemoX-NAD sensors in U-2 OS cells. **a**, Confocal images of U-2 OS cells co-expressing ChemoB-NAD and ChemoG-NAD in the cytosol and mitochondria, respectively, labeled with SiR. Shown are the FRET/FP ratio images for each sensor in pseudocolor (LUT = mpl-viridis). Addition of 100 μM MNNG to the cells is indicated with an arrow (after t = 15 min). **b**. Confocal images of U-2 OS cells co-expressing ChemoB-NAD and ChemoG-NAD in the nucleus (NLS) and mitochondria, respectively. Shown are the FRET donor FP and FRET channels as well as the composite of FP or FRET channels of both sensors. **c**. Time course measurement of U-2 OS cells co-expressing ChemoB-NAD and ChemoG-NAD in the nucleus (NLS) and mitochondria, respectively, upon treatment with 100 μM MNNG. Shown are the means (line) and single cell traces (transparent lines) of the FRET/FP ratios of ChemoB-NAD and ChemoG-NAD normalized to 1 at t = 0 min (*n* = 25 cells from 2 biological replicates). **d**. Confocal images of U-2 OS cells co-expressing ChemoB-NAD and ChemoG-NAD in the nucleus (NLS) and mitochondria, respectively. Shown are the FRET/FP ratio images for each sensor in pseudocolor (LUT = mpl-viridis). Addition of 100 μM MNNG to the cells is indicated with an arrow (after t = 15 min). Scale bars = 25 μm. Source data

By using two spectrally compatible biosensors of the ChemoX–NAD palette, we were able to monitor the fluctuation of free NAD^+^ in real time in two subcellular compartments of U-2 OS cells, that is, ChemoB–NAD_SiR_ and ChemoG–NAD_SiR_ located in the cytosol and mitochondria, respectively. The sensors showed negligible cross-talk between the different emission channels (Fig. 4g). After treatment of the cells with *N*-methyl-*N*′-nitro-*N*-nitrosoguanidine (MNNG), an alkylating agent that is known to lead to hyperactivation of the NAD^+^-consuming enzyme PARP1 (ref. ^35^), we observed a rapid depletion of cytosolic NAD^+^, while mitochondrial NAD^+^ showed a slower, less pronounced decrease (Fig. 4h and Extended Data Fig. 8a). A similar trend was observed using ChemoB–NAD_SiR_ and ChemoG–NAD_SiR_ coexpressed in the nucleus and mitochondria, respectively (Extended Data Fig. 8b–d). In the cytosol and nucleus, NAD^+^ depletion was observed only 5 min after addition of MNNG and was complete in less than 30 min. Previous observations showed that during genotoxic stress induced by alkylating agents, mitochondrial NAD^+^ is maintained longer than cytosolic NAD^+^^36^. However, the multiplexing of biosensors here revealed that some cells experience a decrease in mitochondrial NAD^+^ comparable to the decrease observed in the cytosol and nucleus, indicating a cell-to-cell heterogeneity in response to genotoxic stress.

### ChemoX-based intensiometric and lifetime sensors for NAD^+^

**Fig. 5:**
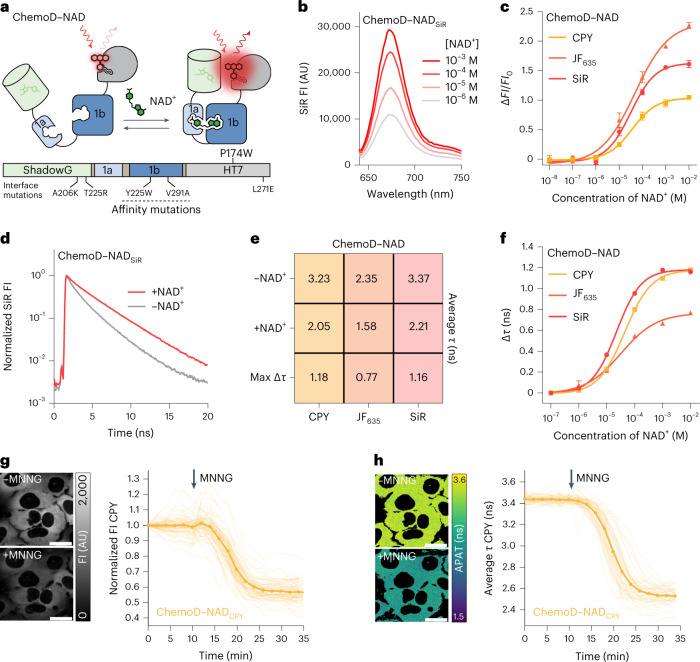
Development of far-red NAD^+^ biosensors based on fluorescence intensity and fluorescence lifetime. **a**, Schematic representation of ChemoD–NAD. **b**, Fluorescence intensity emission spectra of SiR-labeled ChemoD–NAD at different NAD^+^ concentrations. Means of three technical replicates are shown. **c**, NAD^+^ titration curves of ChemoD–NAD labeled with CPY, JF_635_ or SiR. Data are shown as the means ± s.d. of the fluorescence intensity changes (Δ*FI*/*FI*_0_; *n* = 3 technical replicates). Δ*FI*/*FI*_0_ and C_50_ values are summarized in Supplementary Table 9. **d**, Fluorescence lifetime decay curves of ChemoD–NAD_SiR_ in the presence of 1 mM NAD^+^ (+NAD^+^) or absence of NAD^+^ (–NAD^+^). **e**, Intensity-weighted average fluorescence lifetimes (*τ*) of ChemoD–NAD labeled with different fluorophores. Shown are the mean intensity-weighted average fluorescence lifetimes in the presence of 1 mM NAD^+^ (+NAD^+^) or absence of NAD^+^ (–NAD^+^) and the change in lifetime (Δ*τ*; *n* = 3 technical replicates). **f**, NAD^+^ titration curves of ChemoD–NAD labeled with CPY, JF_635_ or SiR. Shown are the means ± s.d. of the intensity-weighted average fluorescence lifetime changes (Δ*τ*; *n* = 3 technical replicates). Δ*τ* and C_50_ values are summarized in Supplementary Table 10. **g**,**h**, Confocal images of U-2 OS cells expressing ChemoD–NAD labeled with CPY. Images are representative snapshots of the CPY fluorescence intensity channel (**g**) or average photon arrival time (APAT; **h**) before (–MNNG) and after (+MNNG) 100 µM MNNG treatment. Time course measurements of ChemoD–NAD_CPY_ fluorescence intensity normalized to 1 at *t* = 0 min (**g**; *n* = 86 cells from three biological replicates) and intensity-weighted average fluorescence lifetime (**h**; *n* = 55 cells from three biological replicates) in U-2 OS cells are shown next to the confocal images corresponding to the same treatments. Represented are the means (lines) and traces of single cells (dim lines). Addition of 100 µM MNNG is indicated with arrows; scale bars, 25 µm. Source data

**Extended Data Fig. 9:**
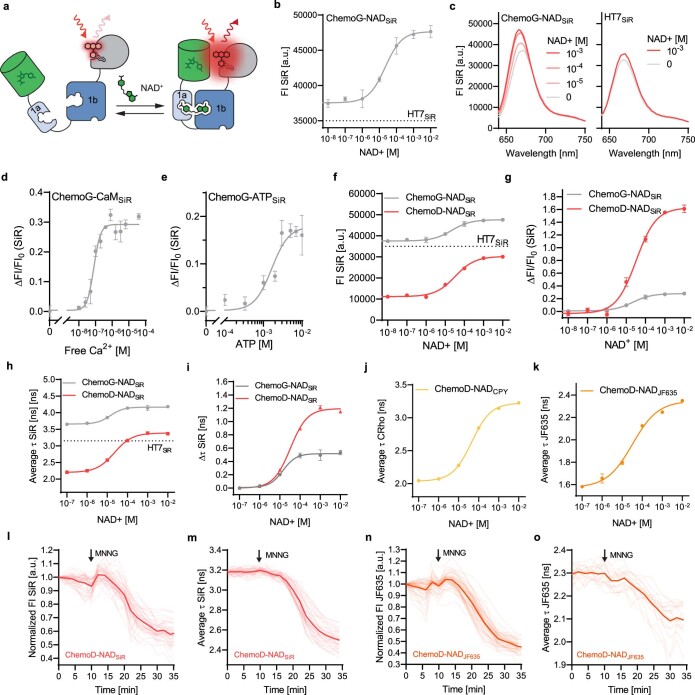
Engineering and characterization of intensiometric and fluorescence lifetime-based sensors. **a**. Schematic representation of intensiometric readout of ChemoG-NAD based on the labeled fluorophore. Closing of the sensor changes the environment of the rhodamine fluorophore and might thereby affect its photophysical properties. **b**. NAD^+^ titration of intensiometric ChemoG-NAD_SiR_ biosensor. Plotted are the fluorescence intensities (FI) of directly excited SiR labeled to ChemoG-NAD. The fluorescence intensity of HT7 labeled with SiR in presence of 1 mM NAD^+^ is indicated with a dotted line. **c**. Fluorescence intensity (FI) emission spectra of SiR-labeled ChemoG-NAD (*left*) or HT7 (*right*) at different NAD+ concentrations. Shown are the means of 3 technical replicates. **d, e**. Titrations of ChemoG-CaM (d) and ChemoG-ATP (e) biosensors labeled with SiR. Plotted are the fluorescence intensity changes (ΔFI/FI_0_) of directly excited SiR. **f**, **g**. NAD^+^ titrations of intensiometric NAD^+^ biosensors labeled with SiR. Plotted are the fluorescence intensities (FI) of directly excited SiR (f) or the change in fluorescence intensity of SiR (ΔFI/FI_0_, g). The fluorescence intensity of HT7 labeled with SiR in presence of 1 mM NAD^+^ is indicated with a dotted line. **h**-**k**. NAD^+^ titrations of fluorescence lifetime-based NAD^+^ biosensors labeled with SiR (**h**, **i**), CPY (**j**) or JF_635_ (**k**).Plotted are the intensity-weighted average fluorescence lifetimes (τ, **h**, **j**, **k**) or the change in τ (Δτ, **i**) of the directly excited fluorophores. The fluorescence lifetime of HT7 labeled with SiR in presence of 1 mM NAD^+^ is indicated with a dotted line in **h**. **l-o**. Time course measurements of ChemoD-NAD fluorescence intensity (FI) (**l, n**) or intensity-weighted average fluorescence lifetime (**m, o**) in U-2 OS cells labeled either with SiR (**l, m**) or JF_635_ (**n, o**). FI was normalized to 1 at t = 0 min. Represented are the means (solid line) plus traces of single cells (dim lines). Addition of 100 μM MNNG is indicated with an arrow. *n* = 44 cells from two independent experiments (**l**), *n* = 37 cells from two independent experiments (**m**), *n* = 53 cells from 2 independent experiments (**n**), *n* = 19 cells from one experiment (**o**). For all titrations, the mean ±s.d. from 3 technical replicates are represented. Source data

ChemoX FRET sensors involve rhodamine fluorophores whose photophysical properties can be influenced by the environment^37^. We therefore hypothesized that ChemoX sensors could be converted into intensiometric and fluorescence lifetime-based sensors as the sensor’s conformational change would likely affect the environment of the rhodamine and thereby its photophysical properties (Extended Data Fig. 9a). The fluorescence intensity of SiR in the context of ChemoG–NAD_SiR_ already showed a 28.0 ± 1.9% increase after NAD^+^ addition (Extended Data Fig. 9b), while the fluorescence intensity of isolated HT7_SiR_ remained mostly unaffected by NAD^+^ (Extended Data Fig. 9c). Similar SiR fluorescence intensity increases were also observed for ChemoG–CaM_SiR_ and ChemoG–ATP_SiR_ (Extended Data Fig. 9d,e). The dynamic range of ChemoG–NAD_SiR_ (^max^Δ*FI*/*FI*_0_) was further improved by introducing the mutation P174W^HT7^, which is known to reduce the fluorescence intensity of SiR on HT7 (ref. ^37^). eGFP was additionally replaced by the non-fluorescent ShadowG^38^ because it serves only as a scaffolding element, creating the sensor ChemoD–NAD (D standing for dark; Fig. 5a and Extended Data Fig. 9f,g). ChemoD–NAD_SiR_ exhibits a ^max^Δ*FI*/*FI*_0_ of 161 ± 5.0%, reaching a maximum fluorescence intensity comparable to isolated HT7_SiR_ with a C_50_ of 32.7 µM (95% CI: 24.3–42.1 µM; Fig. 5b and Extended Data Fig. 9f). The spectral properties of ChemoD–NAD can be adapted using different fluorophore substrates, among which JF_635_ yielded the highest ^max^Δ*FI*/*FI*_0_ with 226.6 ± 4.3% (Fig. 5c and Supplementary Table 9). The sensor fluorescence intensity change probably occurs through dequenching of the fluorophore induced by conformational change. Indeed, the mutation P174W^HT7^ was shown to quench rhodamine fluorescence at the HaloTag surface^37^, which would correspond to the open state of the ChemoD sensor. Through structural analysis (Supplementary Fig. 9), we further hypothesized that the interaction of ShadowG with HT7 (sensor closed state) might induce a reorientation of P174W^HT7^ and thus lead to the dequenching of the fluorophore. However, we cannot exclude that other factors might contribute to the fluorescence increase, such as a reorientation of the fluorophore or an impact on its open–closed equilibrium in the sensor context. The mutation P174W^HT7^ was previously reported to mostly affect the quantum yield and therefore the fluorescence lifetime of rhodamines at the HT7 surface^37^. We hypothesized that the sensor might also present changes in fluorescence lifetime and could be used for fluorescence lifetime imaging microscopy (FLIM). Indeed, in vitro, the fluorescence lifetime (*τ*) of ChemoD–NAD_SiR_ increased in the presence of NAD^+^ from 2.21 ± 0.01 ns to 3.37 ± 0.01 ns (Fig. 5d and Extended Data Fig. 9h,i), offering a dynamic range (^max^Δ*τ*) of 1.16 ± 0.01 ns and a C_50_ of 22.4 µM (95% CI: 20.6–24.4 µM). ChemoD–NAD can also be combined with other fluorophores, notably carbopyronine (CPY), which showed the largest ^max^Δ*τ* with 1.18 ± 0.01 ns (Fig. 5e,f, Extended Data Fig. 9j,k and Supplementary Table 10).

After treatment of U-2 OS cells with MNNG, ChemoD–NAD_CPY_ was able to monitor in real time the depletion of intracellular NAD^+^ (Fig. 5g,h), resulting in a fluorescence intensity decrease of 43.0 ± 6.1% and a *τ* decrease of 0.91 ± 0.08 ns. Similar trends could be observed with ChemoD–NAD_SiR_ and Chemo–NAD_JF635_ (Extended Data Fig. 9l–o). Consistent with the in vitro titrations (Fig. 5c–f), ChemoD–NAD_JF635_ showed the largest change in fluorescence intensity (Δ*FI*/*FI*_0_ = −54.2 ± 5.1%) but the smallest change in *τ* (Δ*τ* = −0.20 ± 0.08 ns; Extended Data Fig. 9n,o).

### ChemoX-based bioluminescent sensors

**Fig. 6:**
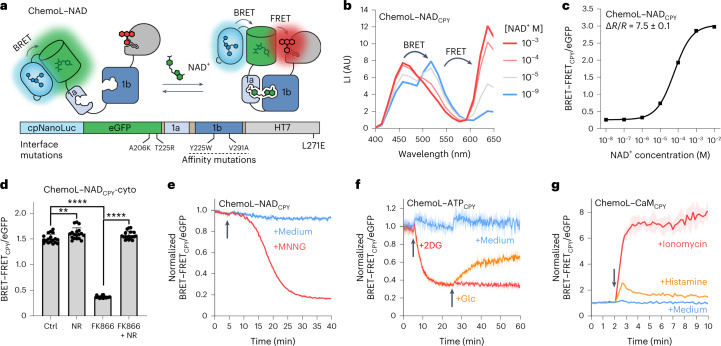
Conversion of ChemoG-based biosensors into luminescent ChemoL biosensors. **a**, Schematic representation of ChemoL–NAD. **b**, Luminescent intensity (LI) emission spectra of CPY-labeled ChemoL–NAD at different NAD^+^ concentrations. Means of three technical replicates are shown. **c**, NAD^+^ titration curve of ChemoL–NAD_CPY_. Shown are the mean BRET–FRET_CPY_/eGFP luminescence ratios ± s.d of three technical replicates. **d**, ChemoL–NAD_CPY_ BRET–FRET/eGFP ratios in U-2 OS cells after treatment for 24 h with DMSO (Ctrl), 1 mM NR, 100 nM FK866 or 100 nM FK866 and 1 mM NR. Represented are the means ± s.d. and single-well ratios (circles; *n* = 18 wells per condition from three biological replicates). *P* values are given based on unpaired two-tailed *t*-tests with Welch’s correction; **P = 0.006; ****P < 0.0001. **e**, Time course measurement of ChemoL–NAD_CPY_ expressed in U-2 OS cells. Represented are the means of the BRET–FRET/eGFP ratios (line) ± s.d. (shaded areas) normalized to 1 at *t* = 0 min. Cells were untreated (+medium) or treated (+MNNG) with MNNG at *t* = 5 min indicated with an arrow (*n* = 3 wells from one representative biological replicate; two additional biological replicates can be found in Supplementary Fig. 10). **f**, Time course measurement of ChemoL–ATP_CPY_ expressed in HeLa Kyoto cells. Represented are the mean BRET–FRET/eGFP ratios (line) ± s.d. (shaded areas) normalized to 1 at *t* = 0 min. Cells were untreated (medium), treated with 2DG at *t* = 5 min (red and orange) and additionally treated with glucose at *t* = 25 min (orange). Addition of medium, 2DG and glucose is indicated with an arrow (*n* = 3 wells from one representative biological replicate; two additional biological replicates can be found in Supplementary Fig. 10). **g**, Time course measurement of ChemoL–CaM_CPY_ expressed in HeLa Kyoto cells. Shown are the mean BRET–FRET/eGFP ratios (line) ± s.d. (shaded areas) normalized to 1 at *t* = 0 min. Cells were untreated or treated with histamine or ionomycin at *t* = 2 min (*n* = 3 wells from one representative biological replicate; two additional biological replicates can be found in Supplementary Fig. 10). Addition of drugs is indicated with an arrow. Source data

**Extended Data Fig. 10:**
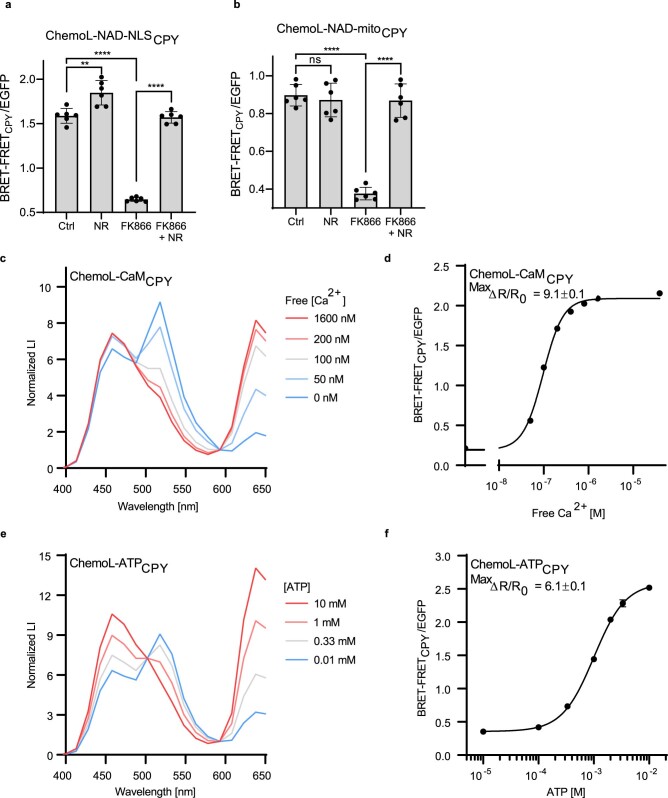
Engineering and characterization of ChemoL sensors. **a, b**. BRET-FRET/eGFP ratios of ChemoL-NAD_CPY_ expressed in the nucleus (**a**) or the mitochondria (**b**) of U-2 OS cells upon treatment for 24 h with DMSO (Ctrl), 1 mM NR, 100 nM FK866 or 100 nM FK866 plus 1 mM NR. Represented are the BRET-FRET/eGFP ratios of single wells (circle) and means ±s.d. (black line) (n = 6 wells from a single experiment). p-values are given based on unpaired two-tailed t-test with Welch’s correction (**** p < 0.0001, ** p = 0.0041, ns p = 0.5718). **c**, **e**. Luminescent intensity (LI) spectra of ChemoL-CaM (**c**) or ChemoL-ATP (**e**) at different concentrations of calcium and ATP, respectively. The spectra were normalized to the isosbestic point at 593 nm. Constructs were labeled with CPY. Shown are the means of 3 technical replicates. **d**, **f**. Analyte titrations of ChemoL-CaM (**d**) and ChemoL-ATP (**f**) labelled with CPY. Shown are the mean ±s.d. of BRET-FRET/eGFP ratios (n = 3 technical replicates). Indicated are also the maximum BRET-FRET/eGFP ratio changes (^Max^ΔR/R_0_). Source data

For high-throughput screening purposes, bioluminescent sensors are particularly interesting, which motivated us to further develop bioluminescent ChemoX-based sensors. Inspired by the Nano-lantern design^39,40^, we fused a circularly permuted NanoLuc to the N terminus of ChemoG–NAD, which gave rise to ChemoL–NAD (L for luminescent), a bioluminescence resonance energy transfer (BRET)–FRET-based sensor for NAD^+^ (Fig. 6a). ChemoL biosensors were combined with CPY because the luminescence reader used could not detect emission wavelengths of >650 nm. ChemoL–NAD_CPY_ exhibits a large dynamic range (^max^Δ*R*/*R*_0_, *R* = BRET–FRET_CPY_/eGFP) of 7.5 ± 0.1-fold and a C_50_ of 60.5 µM (95% CI: 57.3–63.9 µM; Fig. 6b,c). In U-2 OS cells, ChemoL–NAD_CPY_ was able to detect free NAD^+^ concentration changes induced by treatments with FK866 and NR in the cytosol, nucleus and mitochondria (Fig. 6d and Extended Data Fig. 10a,b). Based on the large ratio changes and excellent *Z*′ factors of ChemoL–NAD_CPY_ in different subcellular compartments after treatment with FK866 (cytosol *Z*′ = 0.76, nucleus *Z*′ = 0.79 and mitochondria *Z*′ = 0.52), the sensor represents a powerful tool to be used in high-throughput screenings for compounds influencing free intracellular NAD^+^. Furthermore, the sensor was able to report the compensation of FK866-induced NAD^+^ depletion by simultaneous addition of NR and might therefore be useful for screening of compounds replenishing intracellular NAD^+^.

Similar to ChemoL–NAD, luminescent sensors for calcium and ATP were developed, namely ChemoL–CaM_CPY_ (^max^Δ*R*/*R*_0_ = 9.1 ± 0.1-fold; C_50_ = 96.6 nM; 95% CI: 91.0–102.7 nM; Extended Data Fig. 10c,d) and ChemoL–ATP_CPY_ (^max^Δ*R*/*R*_0_ = 6.1 ± 0.1-fold; C_50_ = 1.00 mM; 95% CI: 0.95–1.05 mM; Extended Data Fig. 10e,f). Using the ChemoL-based biosensors, it was possible to monitor in real time intracellular changes of NAD^+^, ATP and calcium after different drug treatments (Fig. 6e–g and Supplementary Fig. 10a–i). All sensors showed large ratio changes for the respective drug treatments comparable to the responses observed with FRET-based sensors (ChemoL–NAD_CPY_ Δ*R*/*R*_0_ = −74.0 ± 4.4% (MNNG); ChemoL–ATP_CPY_ Δ*R*/*R*_0_ = −70.2 ± 6.0% (2DG); ChemoL–CaM_CPY_ Δ*R*/*R*_0_ = 7.4 ± 0.5-fold (ionomycin); Figs. 2f, 3e and 4h).

## Discussion

FP-based FRET biosensors often suffer from small dynamic ranges. Previous strategies to improve the dynamic range of FRET biosensors involved the use of dimerizing domains^41^, screening for optimized linkers^3,42^ or changing the orientation of the FRET pair^25,43^. Despite some success, these approaches often require a large number of variants to be screened, which hinders the straightforward development of effective sensors. By engineering an interaction interface between a FP and the rhodamine-labeled HaloTag, we developed FRET pairs (ChemoX) with near-quantitative FRET efficiency. Testing no more than five combinations of interface mutations between FP and HaloTag, biosensors with unprecedented dynamic ranges for calcium (36.1-fold), ATP (12.1-fold) and NAD^+^ (34.7-fold) were obtained compared to similar FRET-based biosensors^3,17,25,31,43,44^. Specifically, it was possible to increase the dynamic range of existing biosensors for calcium (YC 3.6) and ATP (ATeam 1.03) by 6.3- and 8.6-fold, respectively, by exchanging the CFP/YFP FRET pair with the ChemoG_SiR_ FRET pair, in addition to shifting the fluorescence excitation and emission to longer wavelengths. To facilitate the development of ChemoX-based biosensors by others, we provide a guideline in the Supplementary Information (Supplementary Figs. 11–13 and Supplementary Notes 1–3). Interface mutations were previously used to improve CFP/YFP-based FRET biosensors but with comparatively moderate success^45^. One of the main limitations for a large dynamic range of CFP/YFP-based FRET biosensors might be the large spectral cross-talk of these FPs. By contrast, the engineered ChemoX interface allows the use of FRET pairs with small spectral overlap, which reduces the FRET efficiency in the open state of the sensor and the spectral cross-talk between the FRET donor and acceptor. The fluorogenic behavior of certain rhodamines in ChemoX biosensors might also enhance the FRET ratio change and thereby contribute to their large dynamic range. Semisynthetic FRET biosensors such as Snifits also reach large dynamic ranges but require engineering of chemicals whose synthesis might not be accessible to non-chemists^12,31,46^. By contrast, ChemoX biosensors only require standard HaloTag fluorophore substrates, broadly used by the microscopy community^22,47–52^. Current ChemoX-based sensors involve sensing domains with large conformational changes, and future efforts will focus on developing sensors able to translate smaller conformational changes into similar dynamic ranges. In light of recent progress in engineering new analyte binding domains^4,5^, we foresee that ChemoX could support their conversion into potent biosensors for new biological activities.

Another key feature of ChemoX resides in its versatility. ChemoX offers an extensive spectral tunability by exchanging the FRET FP donor and using different HaloTag fluorescent substrates as FRET acceptors. The color of the FRET sensors can thus be readily tuned, offering fluorescence excitation and emission maxima ranging from 386 nm to 558 nm and 448 to 668 nm, respectively. For example, the fluorescence maximum emission peaks of ChemoB_SiR_ sensors are separated by more than 200 nm (448 nm for eBFP2 and 668 nm for SiR) and still offer dynamic ranges of up to 12.7-fold. Such sensors can theoretically be spectrally combined with GFP-, YFP- or even RFP-based intensiometric biosensors. We demonstrated this multiplexing ability by monitoring free NAD^+^ in two different cell compartments simultaneously, combining the two sensors ChemoB_SiR_–NAD and ChemoG_SiR_–NAD in fluorescence microscopy. This enabled tracking of the real-time co-regulation of subcellular free NAD^+^ pools in live cells, revealing heterogeneous mitochondrial NAD^+^ regulation after genotoxic stress that could not have been observed in previous studies based on lysates of subcellular fractionation^53,54^. In the future, the multiplexing of biosensors will enable further study of this heterogeneity at the single-cell level. Because the regulation of NAD^+^ inside and between different subcellular compartments plays important roles for key biological processes^28–30,32^, the ChemoX–NAD biosensor palette represents a promising toolbox for the investigation of metabolic and signaling pathways.

Additionally, ChemoX FRET biosensors can be converted into single-channel intensiometric and fluorescence lifetime biosensors, as exemplified for ChemoD–NAD, which showed a ^max^Δ*FI*/*FI*_0_ of 227% (with JF_635_) and ^max^Δ*τ* of 1.18 ns (with CPY). ChemoD–NAD showed better fluorescence intensity changes than some established intensiometric NAD^+^ biosensors^32^. Other intensiometric NAD^+^ biosensors^33^ or biosensors for different biological activities^21,22,55^ show superior dynamic ranges. In the future, it will therefore be interesting to explore whether ChemoD-based biosensors can be improved to reach comparable dynamic ranges. However, ChemoD–NAD showed fluorescence lifetime changes of at least similar magnitudes as current state-of-the-art fluorescence lifetime biosensors^20,56,57^. Based on synthetic far-red fluorophores (maximum emission wavelengths of ≥628 nm), the intensiometric and FLIM-based modalities of ChemoD biosensors bring multiple advantages in terms of brightness, photostability, phototoxicity and autofluorescence compared to FP-based biosensors. Future applications for deep tissue imaging of biological activities can thus be foreseen, as HaloTag can be used in various animal models^52,58^. Furthermore, extending the ChemoD biosensor design to additional orthogonal SLPs should enable multiplexing of such far-red biosensors. Finally, using a BRET–FRET approach, luminescent ChemoL biosensors with dynamic ranges of up to 9.1-fold were developed, offering applications in high-throughput screening. The dynamic ranges of ChemoL biosensors were comparable^59^ or even superior^60,61^ to recently developed BRET sensors.

In conclusion, ChemoX is a chemogenetic platform of FRET pairs that enables the development of biosensors with high dynamic ranges attributed to the reversible interaction of FPs with a fluorescently labeled HaloTag. The color can be readily tuned by either changing the FP or the rhodamine fluorophore substrate, offering multiple options throughout the visible spectrum with the ability for multiplexing. As we demonstrated, the conversion of ChemoX FRET biosensors into intensiometric, fluorescence lifetime-based and bioluminescent biosensors is possible with little effort and through small modifications. ChemoX thus represents an extremely versatile platform, enabling the rapid engineering of potent biosensors that should find broad applications in cell biology.

## Methods

### Reagents, chemicals and fluorophores

Reagents and chemicals were obtained from different manufacturers listed in Supplementary Table 12. Fluorophore chloroalkane (CA) substrates were synthesized according to literature by B. Réssy and D. Schmidt, purchased from commercial vendors (Promega) or provided by L. Lavis or A. N. Butkevich. See Supplementary Table 13 for details. The fluorophore substrates were prepared as 1 mM DMSO stocks, stored at −20 °C and used for all experiments. Milli-Q water was used for all buffers and solutions.

### Plasmids and cloning

Primers for cloning were obtained from Sigma-Aldrich, synthetic genes were purchased from Eurofins, and PCRs were performed using the KOD hot start master mix (Sigma-Aldrich, Merck) according to the manufacturer’s protocol. Sizes of PCR products were verified using standard agarose gel electrophoresis. Gibson assembly^62^ was used as the standard method for cloning. Transformations were performed using standard electroporation. Primers and cloning strategies were designed using Tm Calculator (https://tmcalculator.neb.com) and Geneious software, respectively. Site-directed mutagenesis was performed using the Q5 site-directed mutagenesis kit (New England BioLabs (NEB)) according to the manufacturer’s protocol (transformation via heat shock, *Escherichia coli* strain NEB 5-α) together with the online tool for primer design (NEBaseChanger, https://nebasechanger.neb.com). All plasmids were amplified in the *E. coli* strain *E. cloni* 10G (Novagen), grown at 37 °C and extracted using the QIAprep spin miniprep kit (Qiagen) according to the manufacturer’s protocol, with the exception of the pAAV plasmids (see later). DNA sequences were validated by Sanger sequencing (Eurofins), and DNA was stored at −20 °C until further use. Plasmids are listed in Supplementary Table 14 together with accession codes of plasmids deposited to Addgene and plasmids from Addgene used as template.

The pET-51b(+) vector (Novagen) was used as the backbone for protein expression in *E. coli* (see later). Genes of interest (GOI) were flanked by an N-terminal Strep-tagII and enterokinase cleavage sequence and a C-terminal polyhistidine tag (10× His) for protein purification (for details, see protein sequences in the Supplementary Information). For protein crystallization purposes, genes were flanked only by a N-terminal polyhistidine tag (10× His) followed by a tobacco etch virus (TEV) cleavage sequence.

The pCDNA5/FRT or pCDNA5/FRT/TO vector (Thermo Scientific) was used as the backbone for protein expression in mammalian cells. For targeted expression at subcellular locations, the GOI was flanked by N- and/or C-terminal localization sequences (for details. see protein sequences in the Supplementary Information). Localization sequences were amplified from Addgene plasmids as indicated:

- cytosol, nuclear export signal (NES; Addgene, 101061 (ref. ^63^), a gift from E. Schreiter),
- nucleus, NLS (Addgene, 113931 (ref. ^64^)),
- mitochondria matrix, Cox8 repetitions (Addgene, 113916 (ref. ^31^)),
- outer plasma membrane, IgKchL-PDGFR_tm_ (Addgene, 182009 (ref. ^65^)) and
- inner nuclear membrane, LaminB1 (Addgene, 55069, a gift from M. Davidson).

The NES sequence for cytosolic expression of constructs using pCDNA5/FRT plasmids (LPPLERLTL) was created in the design of the overhangs of PCR primers used for cloning. For coexpression of similar genes (multiplex experiments), codon-optimized sequences for human (*Homo sapiens*, HsOpt) or zebrafish (*Danio rerio*, ZfOtp) were synthesized (Eurofins) to limit the probability of recombination events between the genes. Cotranslational expression was performed using the T2A self-cleaving peptide sequence^66^. The sensor YC 3.6 (ref. ^17^) was reconstructed by molecular cloning from yellow cameleon-Nano140 (Addgene, 51966 (ref. ^44^), a gift from T. Nagai).

The pAAV2-hSyn vector (Addgene, 101061 (ref. ^63^), a gift from E. Schreiter) was used as the backbone for cloning and downstream production of recombinant AAV (rAAV) particles. The GOI was flanked by an N-terminal NES sequence (for details, see the protein sequences in the Supplementary Information). pAAV2-hSyn plasmids were cloned and amplified in *E. coli* strain NEB stable (NEB) and grown at 30 °C in Erlenmeyer flasks.

### Protein expression and purification

*E. coli* strain BL21 (DE3)-pLysS (Sigma-Aldrich) was used for the production of proteins. After electroporation with plasmid DNA, colonies grown overnight on LB agar plates supplemented with 100 µg ml^–1^ ampicillin (Amp) at 37 °C were picked to inoculate 5 ml of liquid LB-Amp and grown overnight at 37 °C and 220 r.p.m. Overnight cultures were diluted 1:200 in 0.1–1 liter of LB-Amp and grown at 37 °C and 220 r.p.m. until reaching an optical density at 600 nm of 0.6–0.8. Protein expression was induced by the addition of 0.5 mM isopropyl-β-d-thiogalactopyranoside, and the cultures were grown at 16 °C for 20–24 h. Cells were collected by centrifugation at 4,500*g* and 4 °C for 10 min. Cell pellets were resuspended in 30 ml of ice-cold lysis buffer (50 mM KH_2_PO_4_, 300 mM NaCl and 5 mM imidazole, pH 8.0) supplemented with 1 mM phenylmethylsulfonyl fluoride and 250 µg ml^–1^ lysozyme. Cells were lysed by sonication (7 min with 50% on/off cycles and 70% amplitude; SONOPULS Bandelin), and cell debris was cleared by centrifugation at 10,000*g* and 4 °C for 15 min. The supernatant was incubated with 0.5–1 ml of Ni-NTA resin (HisPur Ni-NTA Superflow agarose, Thermo Scientific) for 1 h at 4 °C on a roller shaker. The Ni-NTA beads were poured into a 5-ml polypropylene column and washed with 10 column volumes of wash buffer (50 mM KH_2_PO_4_, 300 mM NaCl and 10 mM imidazole, pH 7.5). His-tagged proteins were eluted with 1.5 ml of elution buffer (50 mM KH_2_PO_4_, 300 mM NaCl and 500 mM imidazole, pH 7.5). Subsequently, the buffer was exchanged with activity buffer (50 mM HEPES and 50 mM NaCl, pH 7.3) during protein concentration using Amicon Ultra centrifugal filters (Millipore) with an appropriate molecular weight cutoff.

Protein concentration was determined by absorbance measurements at 280 nm using a Nanodrop 2000c spectrophotometer. The extinction coefficient at 280 nm for the different proteins was extrapolated from the amino acid sequence using Geneious software. Protein purity was confirmed by standard SDS–PAGE using 4–20% Mini-PROTEAN TGX stain-free precast protein gels (Bio-Rad) that were imaged using a GelDoc imager (Bio-Rad). Purified proteins were stored in the presence of 45% (mass/vol) glycerol at −20 °C until further use.

### Protein crystallization

For protein crystallization, HT7 and ChemoG variants were produced and purified as previously reported^15^. Briefly, after Ni-NTA resin purification, the buffer was exchanged to TEV cleavage buffer (25 mM Na_2_HPO_4_ and 200 mM NaCl, pH 8.0). TEV protease (weight ratio of 30:1 protein of interest:TEV) was added to protein samples, and cleavage was performed at 30 °C overnight. After filtering the solution (0.22 μm), the cleaved protein was collected by reverse Ni-NTA resin purification (that is, uncleaved proteins and His-tagged TEV remained bound to the resin, while the cleaved protein of interest was recovered from the flow-through) on a HisTrap FF crude column (Cytiva) using an ÄktaPure FPLC (Cytiva), and the flow-through was collected using the same buffer as for Ni-NTA resin purification (50 mM KH_2_PO_4_, 300 mM NaCl and 10 mM imidazole, pH 7.5). The proteins were further purified by size-exclusion chromatography on a HiLoad 26/600 Superdex 75-pg column (Cytiva), and the buffer was exchanged to activity buffer. The proteins were concentrated to 5 μM using Amicon Ultra 4-ml centrifugal filters (Merck) and fully labeled with 10 µM fluorophore substrate (ChemoG variants with TMR–CA and HT7 with Cy3–CA) for at least 4 h at room temperature (RT). The labeled proteins were then concentrated to ~250 µl, reaching a final concentration between 10 and 16 mg ml^–1^. Proteins were quantified using the absorbance at 280 nm, and the extinction coefficient of the protein was corrected for the fluorophore absorbance at 280 nm using the respective correction factor (CF_280nm_; TMR_CF280nm_ = 0.16, 13,920 M^−1^ cm^−1^; Cy3_CF280nm_ = 0.08, 12,000 M^−1^ cm^−1^).

Crystallization was performed at 20 °C using the vapor-diffusion method. Crystals of HT7–Cy3 were grown by mixing equal volumes of protein solution and a reservoir solution containing 0.2 M magnesium acetate and 19% (mass/vol) PEG 3350. Crystals of ChemoG1–TMR and ChemoG5–TMR were obtained by mixing equal volumes of protein solution and precipitant solution composed of 0.085 M Tris-HCl (pH 8.5), 0.17 M sodium acetate, 15% (vol/vol) glycerol, 27% (mass/vol) PEG 4000 or 0.1 M Tris-HCl (pH 8.5), 0.2 M magnesium chloride and 30% (mass/vol) PEG 4000, respectively. Crystals of HT7–Cy3 and ChemoG5–TMR were briefly washed in cryoprotectant solution consisting of the reservoir solution supplemented with 20% (vol/vol) glycerol before flash-cooling in liquid nitrogen, whereas crystals of ChemoG1–TMR were flash-cooled directly in the mother liquor.

### X-ray diffraction data collection and structure determination

Single crystal X-ray diffraction data were collected at 100 K on the X10SA beamline at the Swiss Light Source (Paul Scherrer Institute). All data were processed with XDS^67^. The structure of HT7–Cy3 was determined by molecular replacement using Phaser^68^ and HT7–TMR coordinates (PDB ID: 6Y7A) as a search model. The structure of ChemoG1–TMR was determined using HT7–TMR (PDB ID: 6Y7A) and GFP (PDB ID: 1GFL) coordinates, and the ChemoG1–TMR model was used to determine the ChemoG5–TMR structure. Geometrical restraints for Cy3 and TMR ligands were generated using the Grade server^69^. The final models were optimized in iterative cycles of manual rebuilding using Coot^70^ and refinement using Refmac5 (ref. ^71^) and phenix.refine^72^. Data collection and refinement statistics are summarized in Supplementary Table 15, and model quality was validated with MolProbity^73^ as implemented in PHENIX.

Atomic coordinates and structure factors have been deposited in the PDB under accession codes 8B6R (HT7–Cy3), 8B6S (ChemoG1–TMR) and 8B6T (ChemoG5–TMR).

### General considerations for fluorescence spectroscopy

Fluorescence measurements were performed in 100 μl of activity buffer supplemented with 0.5 mg ml^–1^ bovine serum albumin (BSA) in black non-binding, flat-bottom 96-well plates (PerkinElmer) unless stated differently. For FRET measurements, proteins were diluted to 200 nM in activity buffer, and HT7-based proteins were additionally labeled with 400 nM fluorophore–CA substrates for 1 h at RT. For fluorescence intensity measurements of intensiometric biosensors, HT7-based proteins were diluted to 1 μM and labeled with 200 nM fluorophore–CA substrates for 1 h at RT. Emission spectra of the proteins were acquired at 37 °C with a Multimode Spark 20M microplate reader (Tecan) using monochromators. Pipetted plates were temperature equilibrated (37 °C) inside the plate reader for 20 min before the measurement. Flash numbers, gain and excitation and emission wavelengths were adjusted depending on the FPs and synthetic fluorophores (for detailed settings, see Supplementary Table 16). Excitation and emission bandwidths were set to 20 nm and 10 nm, respectively, with an emission step size acquisition of 2 nm.

### Analyte titrations of biosensors

Analyte solutions were prepared at 10× final concentration in activity buffer and diluted to 1× final concentration in 100 µl of activity buffer (final volume) in the presence of the labeled proteins in a 96-well plate. For titrations, dilution series of the analyte were prepared in activity buffer. The detailed analyte concentrations are listed in Supplementary Table 17. Activity buffer without analyte was always included as a control.

NAD^+^ titrations in the presence of structurally similar analytes were performed as previously explained but in the presence of the structurally similar analyte (diluted from a 10× solution) and labeled proteins. Activity buffer without NAD^+^ but with a 1× final concentration of the structurally similar analyte was always included as a control.

For calcium titrations, a calcium calibration buffer kit (Life Technologies) was used to precisely control the free calcium concentration. Two buffers containing either 0.1 M CaEGTA or 0.1 M K_2_EGTA were mixed in defined ratios according to the manufacturer’s protocol to generate buffers with free calcium concentrations ranging from 10 nM to 39 µM; 0.1 M K_2_EGTA (0 μM free calcium) was always included as a control. The free calcium concentrations were calculated using equation (1):

1 $$\left[{{\mathrm{Ca}}}^{2+}\right]\text{free}={K}_{{\rm{d}}}^{\text{EGTA}}\times \frac{[{{\mathrm{CaEGTA}}}]}{[{\mathrm{K}}_2{{\mathrm{EGTA}}}]},$$

where ${K}_{{\rm{d}}}^{\text{EGTA}}$ is the dissociation constant of EGTA for calcium in 0.1 M KCl at a given pH and temperature^74^, and [CaEGTA] divided by [K_2_EGTA] is the molar ratio of the two buffers. For the calculation of free calcium, the *K*_d_^EGTA^ at pH 7.2 and 37 °C is 107.9 nM.

For the titration of calcium sensors using the calcium buffers, HT7-based proteins were diluted to 2 μM and labeled with 4 µM fluorophore–CA substrate for 1 h at RT. The calcium sensors were diluted to a final concentration of 200 nM in 20 μl of calcium buffer in a black, low-volume, flat-bottom 384-well plate (Corning).

### Sensitivity assays

The pH sensitivity of the constructs was evaluated using two sodium phosphate-based buffers (SPG pH 4.0 and 10.0, both 1 M; Jena Bioscience) mixed in defined ratios according to the manufacturer’s protocol to yield buffers with different pH values ranging from 5.5 to 8.0. The buffers were diluted tenfold in water (0.1 M final concentration) and supplemented with 0.5 mg ml^–1^ BSA and 50 mM NaCl. The salt concentration sensitivity of the constructs was evaluated using 50 mM HEPES (pH 7.3) buffer supplemented with 0.5 mg ml^–1^ BSA and various NaCl concentrations ranging from 50 to 500 mM NaCl. A condition without salt (0 mM NaCl) was also included.

For the sensitivity assays, the proteins were diluted to 2 μM and labeled with 4 µM fluorophore–CA substrate for 1 h at RT. The labeled proteins were then diluted to 200 nM in the different buffers. For the biosensors, 10× analyte solutions were added and diluted to 1× final concentration. Measurements were conducted as previously explained.

### Calculation of the Förster resonance energy transfer efficiency

The FRET efficiency (*E*) was determined by the fraction of quenched donor fluorescence intensity (FI) using equation (2):

2 $$E=1-\frac{{\mathrm{FI}}_{\mathrm{DA}}}{{\mathrm{FI}}_{\mathrm{D}}},$$

where FI_DA_ and FI_D_ are the maximum FI values of the FRET donor with or without FRET acceptor, respectively.

The Förster radius *R*0 was calculated using equation (3):

3 $$R0=0.211\sqrt[6]{\kappa^ 2\times n-4\times Q_\mathrm{{D}}\times J(\lambda )},$$

where *κ*^2^ is the orientation factor (set to 0.667), *n* is the refractive index (set to 1.33), *Q*_D_ is the quantum yield of the donor (set to the value according to https://www.fpbase.org/ ref. ^75^), and *J*(*λ*) is the spectral overlap of the donor emission and acceptor excitation spectra, which was calculated using equation (4):

4 $$J(\lambda )=\frac{{\int }_{0}^{\infty }\mathrm{FI}_{{\rm{D}}}(\lambda )\times {\varepsilon }_{{\rm{A}}}(\lambda )\times {\lambda }^{4}d\lambda }{{\int }_{0}^{\infty }{F}_{{\rm{D}}}(\lambda )d\lambda },$$

where FI_D_ is the donor fluorescence at the wavelength *λ*, and *ε*_A_(*λ*) is the extinction coefficient of the acceptor at the wavelength *λ*. Spectral overlaps were determined using the software a|e Fluortools (http://www.fluortools.com/software/ae).

### Fluorescent biosensor characterization

From acquired fluorescence emission spectra, the maximum fluorescence intensity values of the FP and/or fluorophore were extracted at their maximum emission wavelengths, defined in Supplementary Table 18. For FRET measurements, the FRET/FP ratios (*R*) were calculated by dividing the maximum fluorescence intensity of the FRET acceptor by the maximum fluorescence intensity of the FRET donor. The dose-dependent response of the FRET biosensors was determined by plotting the ratio change (∆*R*/*R*_0_) over the concentration of the cognate analyte using equation (5):

5 $$\triangle R/{R}_{0}=\frac{{R}_{i}}{{R}_{0}}-1,$$

where *R*_*i*_ is the FRET/FP ratio at a given analyte concentration *i*, and *R*_0_ is the FRET/FP ratio in the absence of analyte. The maximum ratio change (^max^∆*R*/*R*_0_) is calculated with *R*_*i*_ at saturating concentration of analyte.

For fluorescence intensity measurements, the dose-dependent response of the intensiometric sensors was determined by plotting the fluorescence intensity change (∆FI/FI_0_) over the concentration of the cognate analyte using equation (6):

6 $${\triangle F/F}_{0}=\frac{{\mathrm{FI}}_i}{{{\mathrm{FI}}}_{0}}-1,$$

where FI_*i*_ is the fluorescence intensity at a given analyte concentration *i*, and FI_0_ is the fluorescence intensity in absence of the analyte. The maximum fluorescence intensity change (^max^∆FI/FI_0_) is calculated with FI_*i*_ at saturating concentration of analyte.

For fluorescence lifetime (*τ*) measurements, experiments were conducted on a confocal microscope (Leica SP8 Falcon, Leica Microsystems). For details about the determination of *τ* values, see Fluorescence lifetime imaging microscopy. The dose-dependent response of the *τ* sensors was determined by plotting the *τ* change (∆*τ*) over the concentration of the cognate analyte using equation (7):

7 $$\triangle {{\uptau }}={{{\uptau }}}_i-{{{\uptau }}}_{0},$$

where *τ*_*i*_ is the *τ* at a given analyte concentration *i*, and *τ*_0_ is the *τ* in the absence of analyte. The maximum *τ* change (^max^∆*τ*) is calculated with *τ*_*i*_ at the saturating concentration of analyte.

### Data processing and fitting

Data were mathematically processed as explained using Excel (Microsoft). GraphPad Prism (version 8.1.0) was used to fit a sigmoidal dose–response to the titration data (FRET, intensiometric, fluorescence lifetime and BRET–FRET) using equation (8):

8 $$Y={\rm{Bottom}}+\frac{{x}^{H}* ({{\mathrm{Top}}}-{{\mathrm{Bottom}}})}{{x}^{H}+{\mathrm{C}}_{50}^{H}},$$

where *Y* is the response (∆*R*/*R*_0_, ∆FI/FI_0_ or ∆*τ*), Bottom and Top are the lower and upper plateaus of the response, respectively, *x* is the analyte concentration, *H* is the Hill coefficient (that is, slope factor), and C_50_ is the analyte concentration at which the response is half-maximal.

For some data representation, the ratio *R* was chosen instead of ∆*R*/*R*_0_, the FI was chosen instead of ∆FI/FI_0_, and *τ* was chosen instead of ∆*τ*. Data and fit were analyzed using GraphPad Prism software (version 8.1.0).

### Bioluminescence spectroscopy

Bioluminescence measurements were performed in 100 μl of activity buffer supplemented with 0.5 mg ml^–1^ BSA in white, non-binding, flat-bottom 96-well plates (PerkinElmer), except for measurements of calcium sensors, which were performed in 20 µl of calcium buffers (prepared as described earlier in Analyte titrations of biosensors) in white, low-volume, non-binding, flat-bottom 384-well plates (Corning). Proteins were diluted to 200 nM and labeled with 400 nM fluorophore–CA substrate for 1 h at RT. Labeled proteins were further diluted to a final concentration of 0.5 nM together with the 1× final concentration of analyte (as described earlier in Analyte titrations of biosensors) and 1:1,000 diluted Nano-Glo luciferase assay substrate (Promega). Pipetted plates were incubated for 20 min at 37 °C before the measurement to equilibrate the temperature. Bioluminescence emission spectra were acquired with a Multimode Spark 20M microplate reader (Tecan) using the integrated luminescence module at 37 °C. Emission spectra were acquired from 398 to 653 nm with a step size of 15 nm and an integration time of 200 ms.

Luminescent sensors were constructed such that a BRET phenomenon occurred between NanoLuc (maximum emission wavelength observed (${{\mathrm{em}}}\max {\rm{\lambda }}$) = 460 nm) and eGFP (${{\mathrm{em}}}\max {\rm{\lambda }}$ = 518 nm) and a BRET–FRET phenomenon between eGFP and the fluorophore CPY (${{\mathrm{em}}}\max {\rm{\lambda }}$ = 638 nm). The BRET–FRET/eGFP ratios (*R*) were calculated by dividing the maximum luminescence intensity value of the BRET–FRET acceptor CPY by the maximum luminescence intensity value of the BRET–FRET donor eGFP. The dose-dependent response of the luminescent biosensors was determined by plotting the ratio change (∆*R*/*R*_0_) over the concentration of the cognate analyte following equation (5). The maximum ratio change (^max^∆*R*/*R*_0_) was calculated with the ratio at saturating concentration of analyte. A sigmoidal dose–response was fitted to the titration data using equation (8), from which the C_50_ values of the sensors were extrapolated.

### Cell culture

HeLa Kyoto (RRID: CVCL_1922)^76^ and U-2 OS Flp-In T-REx cells^77^ were cultured in high-glucose (4.5 g liter^–1^) DMEM + GlutaMAX medium (Gibco) supplemented with 10% heat-inactivated fetal bovine serum (Gibco). Cells were cultured at 37 °C and 5% CO_2_ in a humidified cell culture incubator. Cells were handled under a sterile laminar flow hood and kept in culture for a maximum of 4 weeks and were passage every 2–4 days or at confluency. Contamination with *Mycoplasma* was regularly checked by PCR. No contamination was detected in the course of this study.

### Generation of stable cell lines

Stable cell lines were generated using the Flp-In T-REx system^77^. U-2 OS Flp-In T-REx cells were grown to 80% confluency in a T-25 cell culture dish and were cotransfected with a pCDNA5/FRT or pCDNA5/FRT/TO plasmid encoding the GOI and the plasmid pOG44 (Invitrogen) in a 1:10 ratio (total of 4 μg of DNA) using Lipofectamine 3000 transfection reagent (Invitrogen) according to the manufacturer’s protocol. Fourteen to 16 h after transfection, the transfection mix was exchanged with fresh cell culture medium supplemented with 100 μg ml^–1^ hygromycin B (Thermo Scientific) to select cells that stably integrated the plasmid into the genome. After 48 h of selection, cells were recovered in fresh cell culture medium until confluency. Cells were sorted in bulk (total of 100,000 cells) for moderate expression levels of the GOI by fluorescence-activated cell sorting using a FACSMelody cell sorter (BD Biosciences). Cells were sorted for eGFP fluorescence (blue laser, 488 nm with 530/30 bandpass filter), or, in case eGFP was not encoded by the GOI, the cells were labeled with SiR-Halo (SiR–CA) and sorted for SiR fluorescence (red laser, 640 nm with 660/10 bandpass filter). For cells that integrated a pCDNA5/FRT/TO plasmid, protein expression was induced by 200 ng ml^–1^ doxycycline for 24 h before sorting or labeling with SiR-Halo. Stable cell lines generated in this study are listed in Supplementary Table 14.

### Transient transfection of mammalian cell lines

For transient transfections, 30,000 or 200,000 cells were seeded into a black, glass-bottom 96- or 24-well imaging plate (Cellvis), respectively, and reverse transfected with pCDNA5/FRT or pCDNA5/FRT/TO plasmid DNA using Lipofectamine 3000 transfection reagent (Invitrogen) according to the manufacturer’s protocol. Cells were incubated with the transfection mix for 8–12 h before the medium was exchanged with fresh cell culture medium.

### Labeling of mammalian cell lines

For transiently transfected cells, cells were incubated for 12 h in cell culture medium supplemented with 500 nM fluorophore–CA substrate 24 h after transfection to achieve labeling of the constructs. For stable cell lines, 24 h before cell labeling, 10,000 or 50,000 cells were seeded into a black, glass-bottom 96- or 24-well imaging plate (Cellvis), respectively. For stable cell lines that integrated a pCDNA5/FRT/TO plasmid, 200 ng ml^–1^ doxycycline was added to the medium for 24 h to induce protein expression. Cells were then labeled as explained earlier for transiently transfected cells. Cells that express GOIs not encoding HT7 were incubated in normal cell culture medium. Excess fluorophore–CA substrate was removed after labeling by washing the cells three times for 5, 15 and then 30 min in phenol red-free cell culture medium (Gibco) before imaging.

Staining of the nucleus and mitochondria with Hoechst 33342 (Invitrogen) and Mitotracker Red FM (Invitrogen), respectively, was performed according to the manufacturer’s protocols. For colocalization experiments, cells expressing biosensors were not labeled with fluorophore–CA substrates to avoid potential spectral cross-talk with MitoTracker Red FM. Images were acquired on a confocal microscope (as described later). Images of Hoechst 33342-stained cells were acquired at an excitation wavelength of 355 nm and emission wavelengths of 400–450 nm. Images of Miotracker Red FM-stained cells were acquired at an excitation wavelength of 600 nm and emission wavelengths of 620–670 nm.

### Preparation of neuron cultures

Before preparation, black, glass-bottom 24-well imaging plates were coated with 100 μg ml^–1^ poly-l-ornithine (diluted in water) for 20 min at RT, washed twice with 1× PBS (pH 7.4) and coated with 1 μg ml^–1^ laminin (dissolved in 1× HBSS) for 1 h at RT. Hippocampi were isolated from newborn rat pups (0–1 days, Wistar rats), as described previously^78^. Procedures were performed in accordance with the Animal Welfare Act of the Federal Republic of Germany (Tierschutzgesetz der Bundesrepublik Deutschland, TierSchG) and the Animal Welfare Laboratory Animal Regulations (Tierschutzversuchsverordnung). According to the TierSchG and the Tierschutzversuchsverordnung, no ethical approval from the ethics committee is required for the procedure of euthanizing rodents for subsequent extraction of tissues. The procedure for euthanizing rats performed in this study was supervised by animal welfare officers of the Max Planck Institute for Medical Research and was conducted and documented according to the guidelines of the TierSchG (permit number assigned by the Max Planck Institute for Medical Research: MPI/T-35/18).

In brief, the brain was extracted by dissecting the skull cap in a posterior–anterior direction. The hippocampi were removed and placed in ice-cold 1× HBSS in the presence of 0.25% trypsin (final concentration) and incubated for 20 min at 37 °C. Tryptic digestion was quenched by the addition of DMEM (Gibco) containing 10% heat-inactivated fetal bovine serum. Neurons were centrifuged at 200*g* for 5 min at RT, washed three times with 1× HBSS and resuspended in 5 ml of phenol red-free Neurobasal medium (NB; Gibco). Neurons were mechanically separated using a pipette until a homogeneous solution was obtained. The solution was filtered through a cell strainer (40-μm pore diameter), and live-cell numbers were determined using the Countess II FL automated cell counter (Thermo Scientific). Fifty-five thousand cells were seeded per well of a precoated 24-well imaging plate in 1 ml of NB medium. Two hours after seeding, medium was exchanged with fresh NB medium. Neurons were kept in a humidified cell culture incubator at 37 °C and 5% CO_2_ and handled under a sterile laminar flow hood. All reagents were sterile filtered with a 0.2-μm filter. The 1× HBSS used during preparation was supplemented with 1× penicillin/streptomycin (Pen/Strep; Gibco) and 200 μM kynurenic acid. NB medium used during neuron preparation and culturing was supplemented with 1× Pen/Strep, GlutaMAX and B27.

### Generation of recombinant adeno-associated viruses

rAAVs were obtained using pAAV2-hSyn plasmids that were extracted from *E. coli* NEB stable bacteria using the GeneJET endo-free plasmid maxiprep kit (Thermo Fisher) according to the manufacturer’s protocol, and the proper open reading frame and inverted terminal repeat sequences were verified by Sanger sequencing (Eurofins). rAAVs were generated as described previously^79^. In brief, plasmids pRV1 (AAV2 Rep and Cap sequences), pH21 (AAV1 Rep and Cap sequences), pFD6 (adenovirus helper plasmid) and the AAV plasmid containing the recombinant expression cassette flanked by AAV2 packaging signals (inverted terminal repeats) were transfected via polyethylenimine 25,000 (Sigma-Aldrich) into HEK293 cells (ACC305, DSMZ^80^). Five days after transfection, the medium and cells were collected by centrifugation at 1,000*g* for 5 min at 4 °C. The cells were lysed using TNT extraction buffer (20 mM Tris (pH 7.5), 150 mM NaCl, 1% Triton X-100 and 10 mM MgCl_2_). Cell debris was removed by centrifuging at 3,000*g* for 5 min at 4 °C. The supernatant was treated with 50 U ml^–1^ benzonase (final concentration; Sigma-Aldrich) for 30–60 min at 37 °C (samples were inverted every 20 min to mix the content). rAAVs were purified from the supernatant via Äkta-Quick FPLC (Cytiva) using AVB Sepharose HiTrap columns (Cytiva). The columns were equilibrated with PBS (pH 7.4), and the virus particles were eluted with 50 mM glycine-HCl (pH 2.7). Purified virus particles were concentrated, and the buffer was exchanged to PBS (pH 7.3) using Amicon Ultra centrifugal filters (Millipore) with a molecular weight cutoff of 100 kDa. rAAVs were aliquoted in 10 µl, flash-frozen and stored at −80 °C until further use. The precise rAAV titer for AAV2/1-hSyn1-NES-ChemoG-CaM was 1 × 10^13^ particles per ml as evaluated by quantitative PCR (genome copies), as described previously^81^.

### Adeno-associated virus transduction and labeling of rat hippocampal neurons

Cultured rat hippocampal neurons were transduced with rAAVs after 8 days in vitro. rAAVs (~5 × 10^9^ particles, 0.5 μl) were diluted in 50 μl of phenol red-free NB medium and added to the medium (1 ml) of the cultured neurons. After 12 days in vitro, neurons were labeled with 200 nM fluorophore–CA substrate (final concentration) by adding 100 µl of phenol red-free NB medium supplemented with 2 μM fluorophore–CA substrate to the ~1 ml of NB medium in which the AAV-transduced neurons were cultured. After 12 h of labeling, neurons were used for live-cell imaging on a widefield microscope (for experimental details, see Widefield microscopy) without washing out the excess fluorophore–CA substrate.

### Confocal microscopy

Confocal microscopy experiments were performed on a laser-scanning confocal microscope (Leica SP8, Leica Microsystems) equipped with a Leica TCS SP8 X scanhead, an HC PL Apo ×40/1.10-NA water motCORR CS2 objective, a SuperK white light laser, a 405-nm diode laser and hybrid photodetectors for single-molecule detection (HyD SMD). The microscope was maintained in an environmental chamber with temperature control set to 37 °C, CO_2_ control set to 5% and humidity control set to 68%. The imaging plate was placed on the confocal microscope stage and temperature equilibrated for 30 min. Confocal images were recorded at a resolution of 512 × 512 pixels (12 or 16 bit), with a scan speed of 400 Hz, pixel dwell time of 3.16 μs, pinhole size of 1 Airy unit and laser pulse rate of 80 MHz, unless stated differently. A sequential scan mode (between frames) was used for imaging with multiple excitation wavelengths. *Z* stacks were acquired where necessary with a step size of 1 μm. For further details, notably excitation and emission settings, see Supplementary Table 18.

### Widefield microscopy

Widefield microscopy experiments were performed on a DMi8 widefield microscope (Leica, Leica Microsystems) equipped with an HC PL Apo ×20/0.80-NA dry objective and an external filter wheel (Leica Microsystems). The microscope was maintained in an environmental chamber with a temperature of 37 °C and a CO_2_ concentration of 5%. The imaging plate was placed on the widefield microscope stage and temperature equilibrated for 30 min. Widefield images of mammalian cells were recorded at a resolution of 512 × 512 pixels (16 bit), with an exposure time of 500 ms, 4 × 4 binning and a cycle time of 766 ms. Widefield images of rat hippocampal neurons were recorded at a resolution of 256 × 256 pixels (16 bit), with an exposure time of 50 ms, 8 × 8 binning and a cycle time of 81 ms. *Z* stacks were acquired where necessary with a step size of 1 μm. eGFP fluorescence was acquired using a 470-nm LED together with a 474/24-nm bandpass filter for excitation and a 525/50-nm bandpass filter for emission detection. FRET fluorescence was acquired using a 470-nm LED together with a 474/24-nm bandpass filter for excitation and a 700/75-nm bandpass filter for emission detection (for further details, see Supplementary Table 18).

### Fluorescence lifetime imaging microscopy

FLIM experiments were performed on a confocal microscope (Leica SP8, Leica Microsystems; as described above) containing the Falcon system (Leica). The microscope was maintained in an environmental chamber with a temperature control set to 37 °C, CO_2_ control set to 5% and humidity control set to 68%.

For in vitro titrations of *τ* biosensors, proteins were diluted to 2 μM in activity buffer supplemented with 0.5 mg ml^–1^ BSA and labeled with 500 nM fluorophore–CA substrate for 1 h at RT. Labeled sensors were mixed with different analyte concentrations (as described in Analyte titrations of biosensors) inside a black, glass-bottom 96-well imaging plate (Cellvis). The pipetted plates were placed on the confocal microscope stage and were temperature equilibrated at 37 °C for 30 min. The confocal volume was focused to the maximum fluorescence intensity of the labeled fluorophore, and images were acquired and processed as for FLIM cell images (explained thereafter). Fluorescence lifetime values were used to characterize the sensor behavior in terms of maximum response (^max^Δ*τ*) and C_50_, as explained earlier.

For live-cell experiments, stable cell lines expressing the *τ* biosensors were seeded and labeled with fluorophore substrates in black, glass-bottom 24-well imaging plates (as described in the Labeling of mammalian cell lines). Imaging plates were placed on the confocal microscope stage and temperature equilibrated for 30 min. The motorized correction ring of the objective was adjusted for each imaging plate (in vitro titrations and live-cell experiments). Excitation and emission detection wavelengths were set up depending on the fluorophore (for details, see Supplementary Table 18). FLIM images were recorded at a resolution of 512 × 512 pixels (8 bit), pinhole size of 1 Airy unit, scan speed of 400 Hz, 12 line repetitions and 1 frame repetition and laser pulse rate of 40 MHz. The laser power was adjusted to obtain less than one photon arrival per laser pulse. The acquired images were intensity thresholded to remove nonspecific background signal. Average fluorescence lifetimes were determined in LAS X Software (Leica Microsystems) using *n*-exponential reconvolution and globally fitting monoexponential (ChemoG–NAD and HT7) or triexponential (ChemoD–NAD) decay models to the decay data. For time course experiments in cells, regions of interest (ROIs) were manually defined for each cell. Cells with saturated fluorescence intensity values or *Χ*^2^ > 1.2 were excluded from the analysis. The calculated average intensity-weighted fluorescence lifetimes are represented. FastFLIM images were used to make figures.

### Live-cell imaging and drug treatment

For time course experiments, transiently or stably expressing cells were seeded in 24-well imaging plates (Cellvis) and labeled, as described above. Media were exchanged 1 h before the start of the time course with the following changes:

- HeLa Kyoto cells, ATP sensors, phenol red-free cell culture medium without glucose (Gibco),
- U-2 OS cells, NAD^+^ sensors, phenol red-free 1× HBSS with calcium and magnesium (Corning) and
- HeLa Kyoto cells, calcium sensors, phenol red-free cell culture medium (Gibco).

Drug treatments were applied directly on the fluorescence microscope during image acquisition (for details about image acquisition, see the corresponding microscope methods described earlier). Stock solutions of drugs were freshly prepared in the same medium/buffer in which the cells were incubated and were prewarmed inside the microscope chamber 30 min before the start of measurement:

- 2× solution of MNNG (200 μM),
- 2× solution of 2DG (20 mM),
- 5× solution of glucose (100 mM) and
- 2× solution of histamine (20 μM).

Using a pipette, the 2× solutions were added in a 1:1 ratio (1 ml:1 ml) and the 5× solution in a 1:4 ratio (0.5 ml:2 ml) to the wells during the time course.

For endpoint measurements of NAD^+^ biosensors, cells were seeded in 96-well imaging plates (Cellvis) and labeled as described earlier. After labeling/washing, cells were incubated in phenol red-free cell culture medium supplemented with 0.01% DMSO (vol/vol; control), 100 nM FK866 (prepared from 1 mM DMSO stock solution) and/or 1 mM NR (prepared from 1 M water stock solution) for 24 h. Cells were maintained in a humidified cell culture incubator at 37 °C and 5% CO_2_ between the preparation steps. After treatment, cells were directly used for imaging by fluorescence confocal microscopy.

### Calcium imaging with Cal520

HeLa Kyoto cells were transiently transfected with a plasmid encoding ChemoR–CaM expression in a black 96-well imaging plate, as described earlier. ChemoR–CaM was chosen because it can be spectrally distinguished from Cal520 in fluorescence microscopy. The transfected cells were loaded with 5 μM Cal520-AM (diluted in imaging medium) for 2 h at 37 °C. Excess extracellular Cal520-AM was removed by washing the cells twice with imaging medium. Cells were incubated for 30 min at 37 °C on the stage of a widefield microscope before the fluorescence of Cal520 (excitation: laser 470 nm (filter 474/24 nm bandpass); emission: 525/50 nm (bandpass)) and ChemoR–CaM (excitation: laser 550 nm (filter 554/23 nm bandpass); emission: 609/54 nm (bandpass)) was recorded. Cells were treated with 10 μM histamine to observe calcium oscillations. For data processing, cells from the same field of view were analyzed and divided into two groups depending on if they expressed ChemoR–CaM or not.

### Absolute quantification of cytosolic calcium concentrations

Calibration curves for the determination of free calcium concentrations were generated by performing calcium titrations in cell lysates or directly in situ. For the titrations in lysates, 8,000,000 HeLa Kyoto cells were transiently transfected with a plasmid encoding ChemoG–CaM in 100-mm dishes and labeled with SiR-Halo, as described earlier. The cells were then washed twice with ice-cold PBS (pH 7.4; without calcium and magnesium) before adding 300 μl of lysis buffer (one cOmplete protease inhibitor cocktail tablet in 10 ml of CellLytic M) to the cells and collecting them with a cell scraper. Cells were vortexed for 10 s, incubated for 10 min on ice and centrifuged at 4,500*g* and 4 °C for 20 min to remove cell debris. The supernatant (cell lysate) was transferred to a fresh tube and directly used for titration experiments. Cell lysate (5 μl) was mixed with 95 μl of calcium calibration buffers (adjusted to different concentrations of free calcium; see previous sections) in a black, 96-well imaging plate and incubated for 30 min at 37 °C on the stage of the same widefield microscope used for live-cell imaging experiments.

For in situ calibration, HeLa Kyoto cells were transiently transfected with a plasmid encoding ChemoG–CaM in a black, 96-well imaging plate and labeled with SiR-Halo, as described earlier. Cells were washed twice with HBSS (without calcium and magnesium) before the addition of calcium calibration buffers (adjusted to different concentrations of free calcium and prewarmed to 37 °C; see previous sections) containing 0.002% digitonin for cell permeabilization. The cells were incubated for 20 min at 37 °C on the stage of the same widefield microscope used for live-cell imaging experiments.

eGFP and FRET fluorescence intensities for both calibration approaches were acquired using the same settings as for live-cell imaging. Image processing and determination of the FRET/eGFP ratios were conducted as described in the previous sections. A non-linear regression model (equation (8)) was fitted to the FRET/eGFP ratios and was used as the calibration curve. The ‘bottom’ and ‘top’ values of the non-linear regression were fixed and set to the FRET/eGFP ratios acquired for 0 μM and 39 μM free calcium, respectively. Absolute calcium concentrations in the cytosol of HeLa Kyoto cells were calculated for treatment with 10 μM histamine (maximum FRET/eGFP ratio) based on the calibration curves (lysate or in situ). The exact calcium concentrations under resting conditions (before treatment, basal) could not be determined because the FRET/eGFP ratios did not match with the non-linear regression model of the calibration measurements.

### Electric field stimulation of neurons

Time course experiments of rat hippocampal neurons expressing calcium sensors were performed in phenol red-free NB medium supplemented with Pen/Strep, GlutaMAX and B27. Rat hippocampal neurons were seeded in black, 24-well imaging plates and imaged on a DMi8 widefield microscope (as described earlier). Thirty minutes before the experiment, 100 µl of phenol red-free NB medium supplemented with a synaptic blocker cocktail (25 μM d,l-2-amino-5-phosphonovaleric acid (SantaCruz) and 10 μM 2,3-dihydroxy-6-nitro-7-sulphamoyl-benzo(F)quinoxaline (Sigma; final concentrations)) was added to the neurons. Neurons were placed on the widefield microscope stage and temperature equilibrated for 30 min. A custom-built 24-well cap stimulator with platinum electrodes inserted into the medium was mounted on top of the imaging plate linked to a stimulation control unit, as previously described^82^. Trains of APs from 1 to 200 APs were evoked by field stimulation at 80 Hz, 100 mA and 1-ms pulse width in 10-s intervals.

### Bioluminescence measurements in mammalian cells

Cells transiently or stably expressing luminescent biosensors were seeded in white cell culture-treated 96-well plates with a transparent bottom (BrandTech) and labeled, as descried earlier. For endpoint measurements of NAD^+^ biosensors, cells were treated as for fluorescence microscopy experiments (as described earlier), washed twice with sterile-filtered 1× PBS (pH 7.4; Gibco) and incubated in 100 μl of phenol red-free cell culture medium supplemented with 1:500 diluted cell-permeable NanoBRET Nano-Glo substrate (Promega) and 1:1,000 diluted cell-impermeable NanoLuc inhibitor (Promega). Cells were then incubated at 37 °C for 30 min before the measurement. Bioluminescence spectra were acquired as described for in vitro characterization.

For time course measurements, cells were washed twice with sterile-filtered 1× PBS (pH 7.4; Gibco) and incubated in 100 μl of the following:

- phenol red-free cell culture medium (calcium sensor),
- phenol red-free cell culture medium without glucose (ATP sensor) or
- sterile-filtered phenol red-free 1× HBSS with calcium and magnesium (NAD^+^ sensor).

All media were supplemented with 1:250 diluted cell-permeable NanoBRET Nano-Glo substrate (Promega) and 1:1,000 diluted cell-impermeable NanoLuc inhibitor (Promega). Cells were then incubated inside a Multimode Spark 20M microplate reader (Tecan) at 37 °C for 30 min before the start of the measurement to equilibrate the temperature. Maximum emission peaks of the BRET–FRET acceptor CPY (625–650 nm) and BRET–FRET donor eGFP (505–530 nm) were recorded during the time course to trace the BRET–FRET/eGFP ratio over time using the same parameter settings as for the acquisition of emission spectra in vitro. Drug treatments were applied directly on the plate reader. Stock solutions were freshly prepared in the same medium/buffer in which the cells were incubated and prewarmed at 37 °C for 30 min before the start of the measurement:

- 2× solution for MNNG (200 μM),
- 2× solution for 2DG (20 mM),
- 5× solution for glucose (100 mM),
- 2× solution for histamine (20 μM) and
- 2× solution for ionomycin (2 μM).

For addition of the reagents, the time course was paused, the 96-well plate was ejected, and solutions were added in a 1:1 ratio (100 μl:100 μl, 2× solutions) or 1:4 ratio (50 μl:200 μl, 5× solution) to the wells using a multichannel pipette. The time course was immediately resumed after addition. Bioluminescence emission spectra were recorded for each well at the end of the time course (as described for in vitro characterization) to verify that the sensor still provided an emission spectra in which each channel strongly provide photons at the end of the treatment.

### Image analysis

All images were analyzed using the software ImageJ^83^. *Z* planes of images (12 or 16 bit) were combined into a composite image by maximum intensity *Z* projection. Background signal was subtracted with a rolling ball radius of 50 pixels. Brightness and contrast were adjusted identically for each channel of a processed image unless stated differently. The processed images were further used for quantitative analysis and figure generation.

For the generation of FRET/FP ratio images, the image of the FRET acceptor channel was duplicated and thresholded (Otsu’s method). A binary mask was created from the thresholded image and applied to the FRET acceptor and FRET donor channels that were subsequently divided (FRET acceptor channel/FRET donor channel) and converted into a 32-bit format, all using the Image calculator function.

For quantitative analysis, ROIs were manually defined for each cell. The fluorescence intensity values of ROIs were measured in the respective channel of background-subtracted images. Cells with saturated fluorescence intensity values were excluded from the analysis. For FRET experiments, the FRET/FP ratios were determined by dividing the measured fluorescence intensity of the FRET acceptor by the measured fluorescence intensity of the FRET donor channel of each ROI. Data were mathematically processed using Excel (Microsoft). Data were visualized by using GraphPad Prism software (version 8.1.0).

For the representation of cells expressing *τ* biosensors, FastFLIM images and their corresponding intensity images acquired during FLIM experiments were exported and further processed. The intensity image was thresholded (Otsu’s method), converted into a binary mask and applied to the FastFLIM image using the Image calculator function. The processed FastFLIM image was used for figure generation, representing the average photon arrival time. FastFLIM images were not used for quantitative analysis of fluorescence lifetimes. Fluorescence lifetimes used for quantitative analysis were determined as described in Fluorescence lifetime imaging microscopy.

### Statistics and reproducibility

All in vitro measurements were performed at least in three technical replicates. All cell experiments were performed at least in three biological replicates (that is, on three different days), unless stated differently. Statistical significance of a sample group over a reference group was determined by performing a two-tailed unpaired *t*-test with Welch’s correction using the Software GraphPad Prism (version 8.1.0). Statistical analyses were performed on sample sizes of >30 cells (for fluorescence microscopy), assuming normal distribution according to the central limit theorem, or on ≥6 wells (for bioluminescence). Sample sizes are indicated in the captions of corresponding figures. The following notations apply for all statistical analyses: NS (not significant) *P* ≥ 0.5, *P < 0.05, **P < 0.01, ***P < 0.001 and ****P < 0.0001. Microscopy images are snapshots representative of three independent experiments.

### Reporting summary

Further information on research design is available in the Nature Portfolio Reporting Summary linked to this article.

## Online content

Any methods, additional references, Nature Portfolio reporting summaries, source data, extended data, supplementary information, acknowledgements, peer review information; details of author contributions and competing interests; and statements of data and code availability are available at 10.1038/s41589-023-01350-1.

## Supplementary information

Supplementary Information Supplementary Figs. 1–13, Tables 1–18, Notes 1–6 and references.

Reporting Summary

Supplementary Data 1 Source data for Supplementary figures.

## Data Availability

The X-ray crystal structures of ChemoG1_TMR_, ChemoG5_TMR_ and HT7_Cy3_ were deposited to the PDB with accession codes 8B6S, 8B6T and 8B6R, respectively. Plasmids of interest from the study will be deposited at Addgene. The data supporting the findings of this study are available within the paper and its Supplementary Information. Source data are provided with this paper. Additional information is available from the corresponding author upon reasonable request.

## Source data

Source Data Fig. 1 Source data.
Source Data Fig. 2 Source data.
Source Data Fig. 3 Source data.
Source Data Fig. 4 Source data.
Source Data Fig. 5 Source data.
Source Data Fig. 6 Source data.
Source Data Extended Data Fig. 1 Source data.
Source Data Extended Data Fig. 2 Source data.
Source Data Extended Data Fig. 3 Source data.
Source Data Extended Data Fig. 4 Source data.
Source Data Extended Data Fig. 5 Source data.
Source Data Extended Data Fig. 6 Source data.
Source Data Extended Data Fig. 7 Source data.
Source Data Extended Data Fig. 8 Source data.
Source Data Extended Data Fig. 9 Source data.
Source Data Extended Data Fig. 10 Source data.
