# Supplementary material for: A general method for the development of multicolor biosensors with large dynamic ranges
Source: Nat Chem Biol. 2023 Jun 8;19(9):1147–57. doi: 10.1038/s41589-023-01350-1 (PMC10449634; doi:10.1038/s41589-023-01350-1)
Supplement: Supplementary file 1 — Supplementary Figs. 1–13, Tables 1–18, Notes 1–6 and references. [file 41589_2023_1350_MOESM1_ESM.pdf]

nature chemical biology

Article https://doi.org/10.1038/s41589-023-01350-1

A general method for the development of multicolor biosensors with large dynamic ranges

In the format provided by the authors and unedited

Table of Contents

Supplementary Figures **3**

Supplementary Figure 1 Initial design of the chemogenetic FRET pair 3

Supplementary Figure 2 Sensitivity of $ChemoG_{SiR}$ to environmental changes. 4

Supplementary Figure 3 ChemoG performance in fluorescence microscopy 5

Supplementary Figure 4 Impact of the fluorophore structure on the FRET efficiency of ChemoG5 6

Supplementary Figure 5 Implementation of different RFPs into the calcium sensor design 7

Supplementary Figure 6 Overexpression of calmodulin-based calcium sensors reduces intracellular calcium oscillations 8

Supplementary Figure 7 Emission spectra of ChemoB-NAD and ChemoR-NAD 9

Supplementary Figure 8 Performance of ChemoB-NAD and ChemoR-NAD in U-2 OS cells 10

Supplementary Figure 9 Structural comparison of ChemoG and $HaloTag7^{P174W}$ 11

Supplementary Figure 10 ChemoL sensor performances in U-2 OS cells 12

Supplementary Figure 11 Development of ChemoG biosensors 13

Supplementary Figure 12 Tuning the spectral properties of the optimized ChemoG sensor .14

Supplementary Figure 13 Tuning the readout mode of the optimized ChemoG sensor 14

Supplementary Tables **15**

Supplementary Table 1 FRET efficiencies of the ChemoG interface variants 15

Supplementary Table 2 FRET ratios of ChemoX constructs expressed in U-2 OS cells 16

Supplementary Table 3 FRET efficiencies of ChemoG5 labeled with different rhodamine fluorophores 17

Supplementary Table 4 FRET efficiencies of ChemoX FRET pairs 18

Supplementary Table 5 Summarizing characteristics of the calcium sensors 19

Supplementary Table 6 Summarizing characteristics of ChemoG-CaM labeled with different FRET acceptors 20

Supplementary Table 7 Summarizing characteristics of ATP sensors 21

Supplementary Table 8 Summarizing characteristics of NAD^+ sensors 22

Supplementary Table 9 Summarizing characteristics of intensiometric NAD^+ sensors 23

Supplementary Table 10 Summarizing characteristics of fluorescence lifetime-based NAD^+ sensors 24

Supplementary Table 11 ChemoG FRET pairs recommended for the development of ChemoG FRET biosensors 25

Supplementary Table 12 Chemicals and reagents used in this study 26

Supplementary Table 13 Fluorophores used in this study 28

Supplementary Table 14 Plasmids and stable cell lines used in this study 30

Supplementary Table 15 Data collection and refinement statistics 32

Supplementary Table 16 Spectral settings for fluorescence spectroscopy measurements 33

Supplementary Table 17 Analyte concentration ranges used for sensor titrations 34

Supplementary Table 18 Settings for confocal and widefield fluorescence microscopy 35
Supplementary Notes 37
Supplementary Note 1 Development of ChemoG biosensors 37
Supplementary Note 2 Tuning the spectral properties of the optimized ChemoG sensor 38
Supplementary Note 3 Tuning the readout mode of the optimized ChemoG sensor. 38
Supplementary Note 4 Protein sequences 39
Supplementary Note 5 Purification sequences 45
Supplementary Note 6 Localization sequences 45
References 46

Supplementary Figures

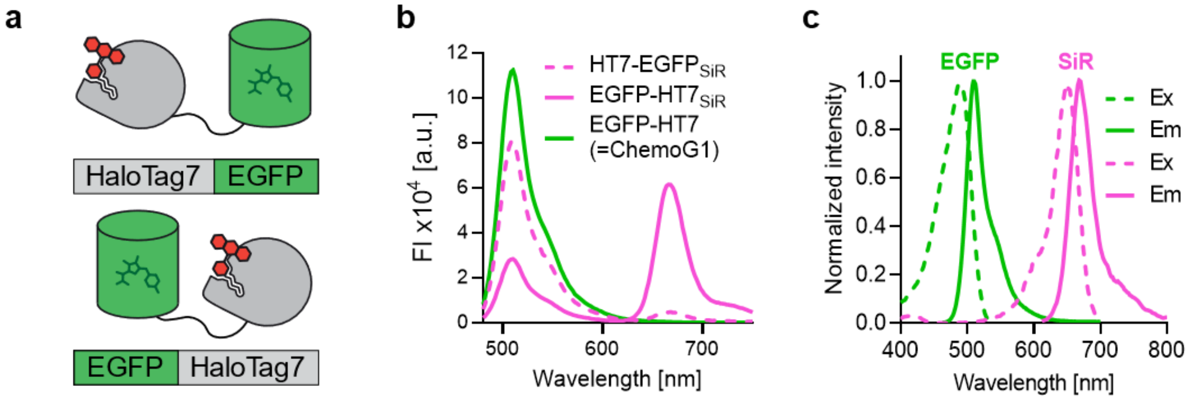

Supplementary Figure 1| Initial design of the chemogenetic FRET pair.

a. Schematic representation of the chemogenetic FRET pair based on EGFP and HaloTag7 (HT7) labeled with a synthetic rhodamine fluorophore. Shown are cartoons of the fusion of HT7 to the N- (HT7-EGFP) or C-terminus of EGFP (EGFP-HT7) **b**. Fluorescence intensity (FI) emission spectra of HT7-EGFP and EGFP-HT7 (= ChemoG1) labeled with SiR or not labeled. Represented are the means of 3 technical replicates. **c**. Normalized excitation (Ex) and emission (Em) spectra of EGFP and SiR. Represented are the means of 3 technical replicates.

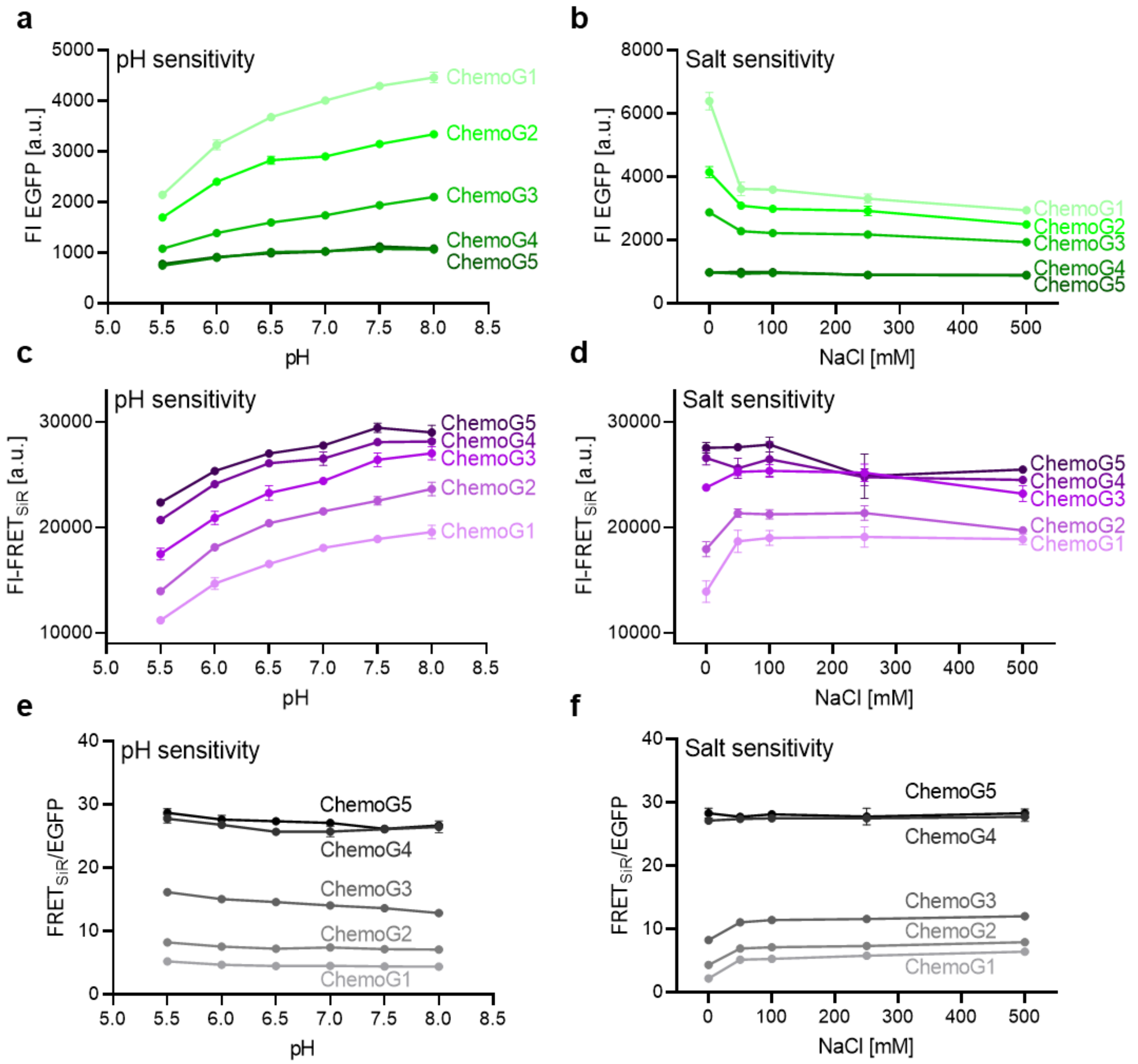

Supplementary Figure 2| Sensitivity of ChemoG$_{SiR}$ to environmental changes.
pH (**a, c, e**) and salt (**b, d, f**) sensitivity of the fluorescence intensity of EGFP (**a**, **b**), FRET (**c**, **d**) and the FRET/EGFP ratio (**e**, **f**) of purified ChemoG constructs labeled with SiR. Shown are the means ±s.d. of 3 technical replicates.

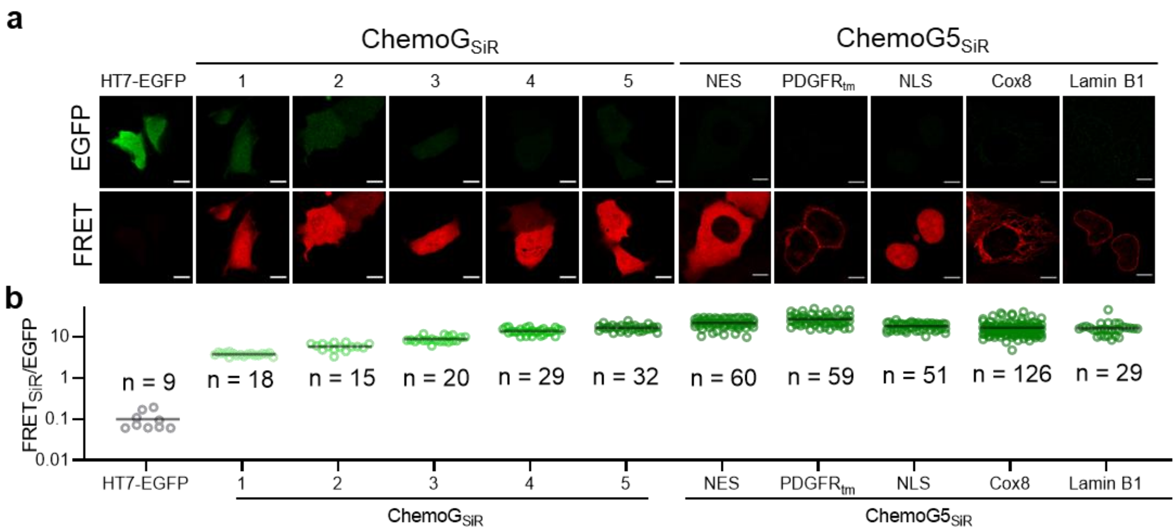

Supplementary Figure 3| ChemoG performance in fluorescence microscopy.
a. Confocal images of U-2 OS cells expressing untargeted HT7-EGFP, untargeted ChemoG1-5 or ChemoG5 targeted to different subcellular localizations. Cells were labeled with SiR. Shown are the EGFP and FRET channels. Scale bars = 10 µm. **b**. FRET/EGFP ratios of U-2 OS cells expressing different ChemoG constructs labeled with SiR as explained in **a**. Plotted for each construct are the FRET/EGFP ratios of individual cells (circles) and the mean (black line). The number of cells acquired for each construct are indicated and are derived from 2 independent experiments.

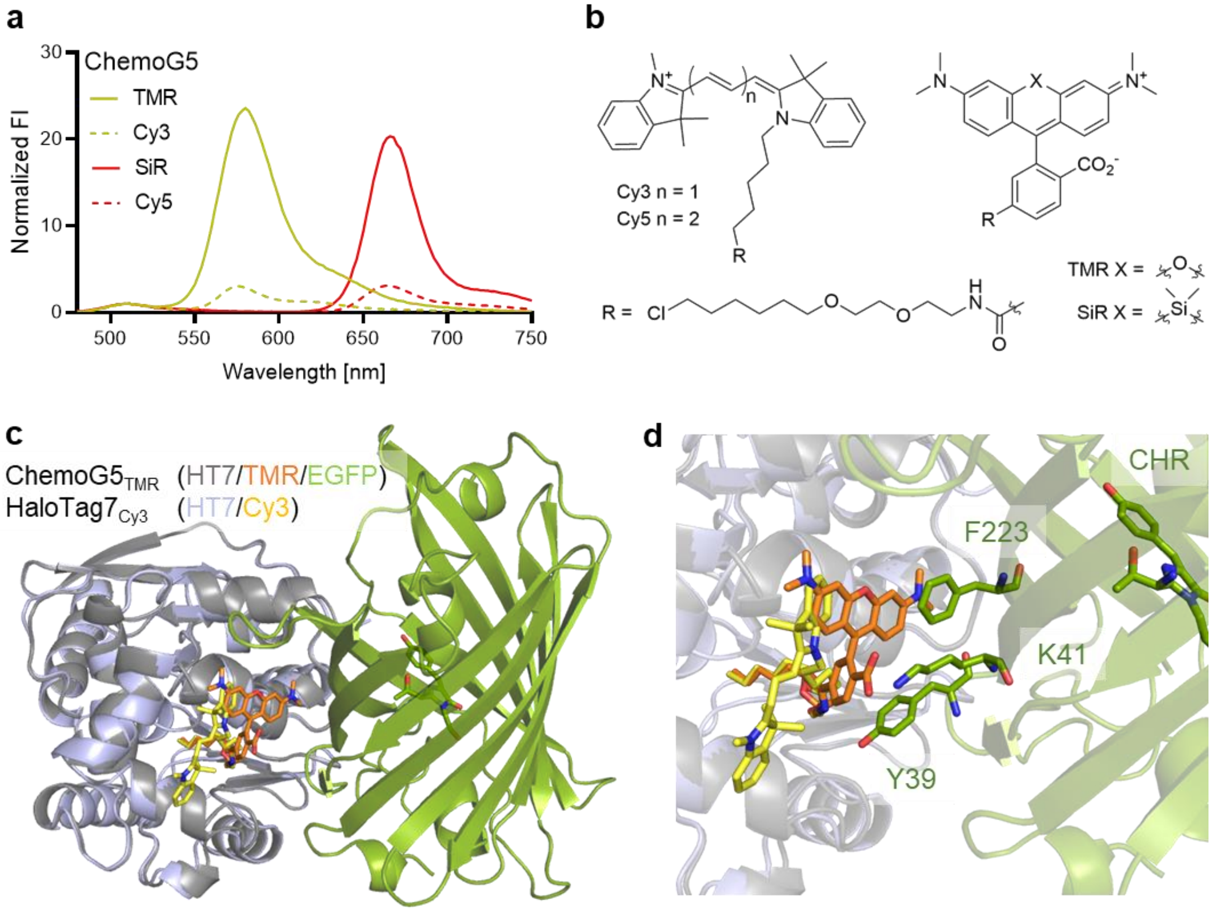

Supplementary Figure 4| Impact of the fluorophore structure on the FRET efficiency of ChemoG5.
a. Normalized fluorescence intensity (FI) emission spectra of ChemoG5 labeled with spectrally similar but structurally different fluorophores. Spectra were normalized to the maximum FI of EGFP. Shown are the means of 3 technical replicates. **b**. Chemical structures of cyanines (Cy3, Cy5) and rhodamines (TMR, SiR) coupled to the chloroalkane substrate (R) for HaloTag7. **c**. Structural comparison of the HaloTag7$_{Cy3}$ (PDB ID: 8B6R) and ChemoG5$_{TMR}$ (PDB ID: 8B6T) X-ray structures. HaloTag7$_{Cy3}$ was structurally aligned with the HaloTag7$_{TMR}$ component of ChemoG5$_{TMR}$. HaloTag7 (grey or light blue) and EGFP (green) are shown as cartoon. The EGFP chromophore (green), TMR (orange) and Cy3 (yellow) are shown as sticks. **d**. Zoom-on the interface HaloTag7/EGFP with representations as described in **c**. Residues Y39, K41 and F223 of EGFP involved in the direct interaction with TMR are annotated and shown as sticks.

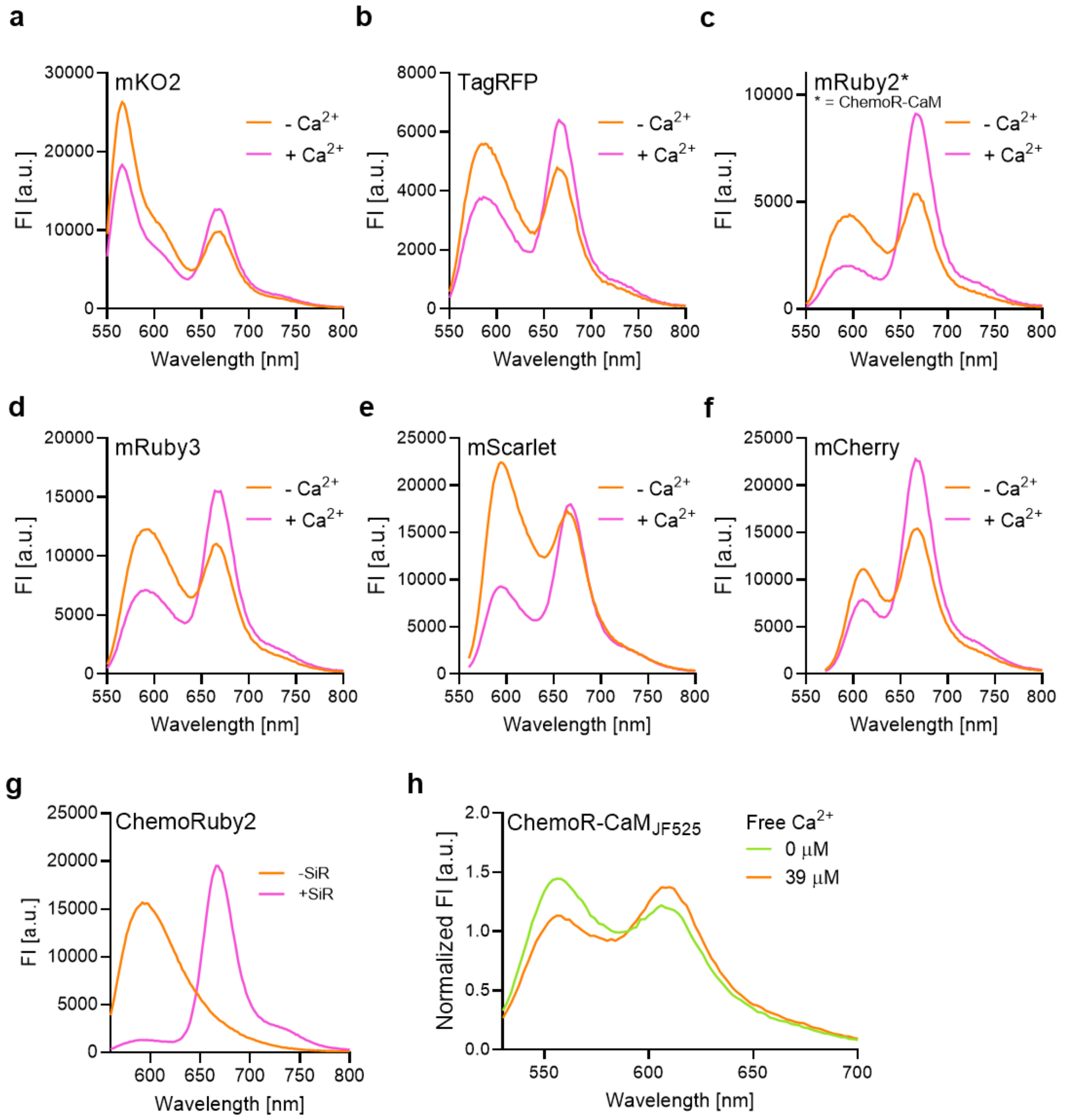

Supplementary Figure 5| Implementation of different RFPs into the calcium sensor design.
a-f. Fluorescence intensity (FI) emission spectra of RFP-CaM/M13-HT7 sensors labeled with SiR in abscence (2 mM EGTA, -Ca^{2+}) or presence (2 mM $CaCl_2$, +Ca^{2+}) of free Ca^{2+}. Different RFPs were used as FRET donor, indicated in the graph. Shown are the means of 3 technical replicates. *mRuby2 was chosen as the final ChemoR-CaM calcium sensor. **g**. FI emission spectra of static ChemoRuby2 labeled with SiR. The FRET efficiency and FRET ratio can be found in **Supplementary Table 4**. **h**. FI emission spectra of ChemoR-CaM labeled with JF_{525} in absence and presence of free Ca^{2+}. JF_{525} served as FRET donor and mRuby2 as FRET acceptor.

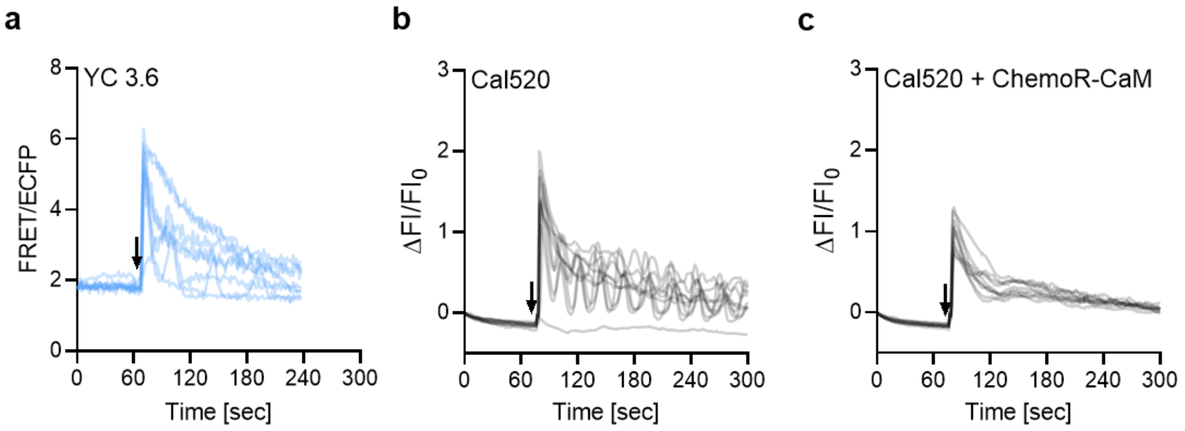

Supplementary Figure 6| Impact of calmodulin-based calcium sensors on intracellular calcium oscillations.
a. Time course measurement of free intracellular calcium fluctuations using yellow cameleon 3.6 (YC 3.6). Represented are the FRET/ECFP ratios for 8 representative single cell traces from 3 biological replicates. HeLa Kyoto cells were treated with 10 µM histamine at the time point indicated with an arrow. Most of the cells show no or reduced calcium oscillations as observed for ChemoX-CaM (Fig. 2f). **b**-**c**. Time course measurements of free intracellular calcium fluctuations using the synthetic calcium indicator Cal520. HeLa Kyoto cells were transiently transfected to express ChemoR-CaM. ChemoR-CaM has been chosen to not interfere spectrally with Cal520. Ten representative single cell traces from 3 biological replicates not expressing (**b**) and expressing (**c**) ChemoR-CaM were analyzed for calcium oscillations upon treatment with 10 µM histamine at the time point indicated with an arrow. Represented are the fluorescence intensity changes (ΔF/F0) of Cal520. Cells that did not express ChemoR-CaM mostly highlight calcium oscillations while the expression of ChemoR-CaM seem to repress this behavior as for YC 3.6, which is as well a calmodulin-based calcium sensor. This phenomenon was already reported in the literature and seems to occur through calcium buffering due to the sensor over-expression[21].

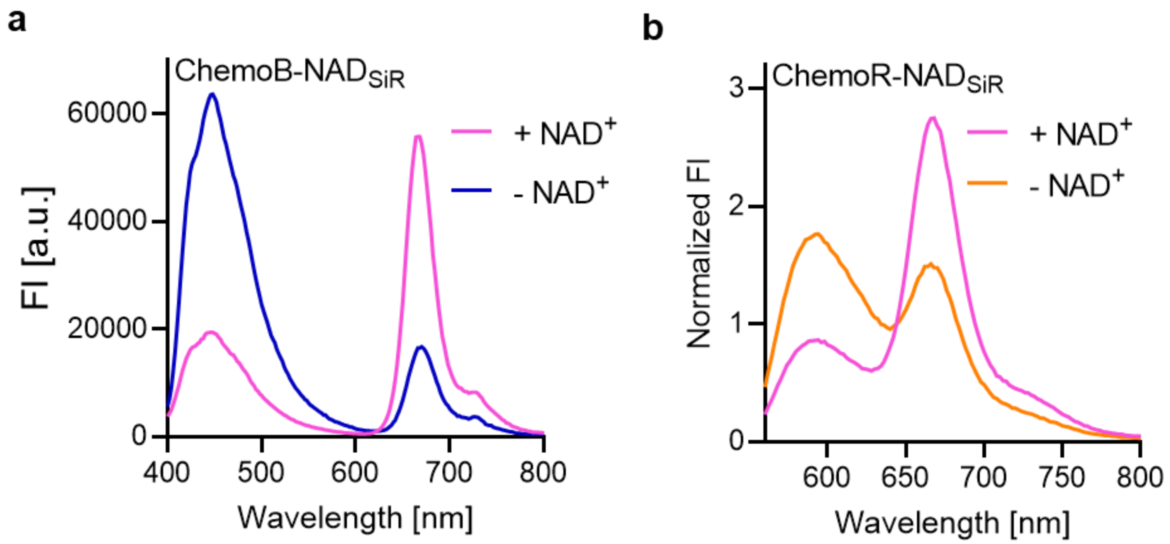

Supplementary Figure 7| Emission spectra of ChemoB-NAD and ChemoR-NAD.
a, **b**. Fluorescence intensity (FI) emission spectra of SiR-labeled ChemoB-NAD (**a**) and ChemoR-NAD (**b**) in presence or absence of 1 mM NAD^+. Shown are the means of 3 technical replicates.

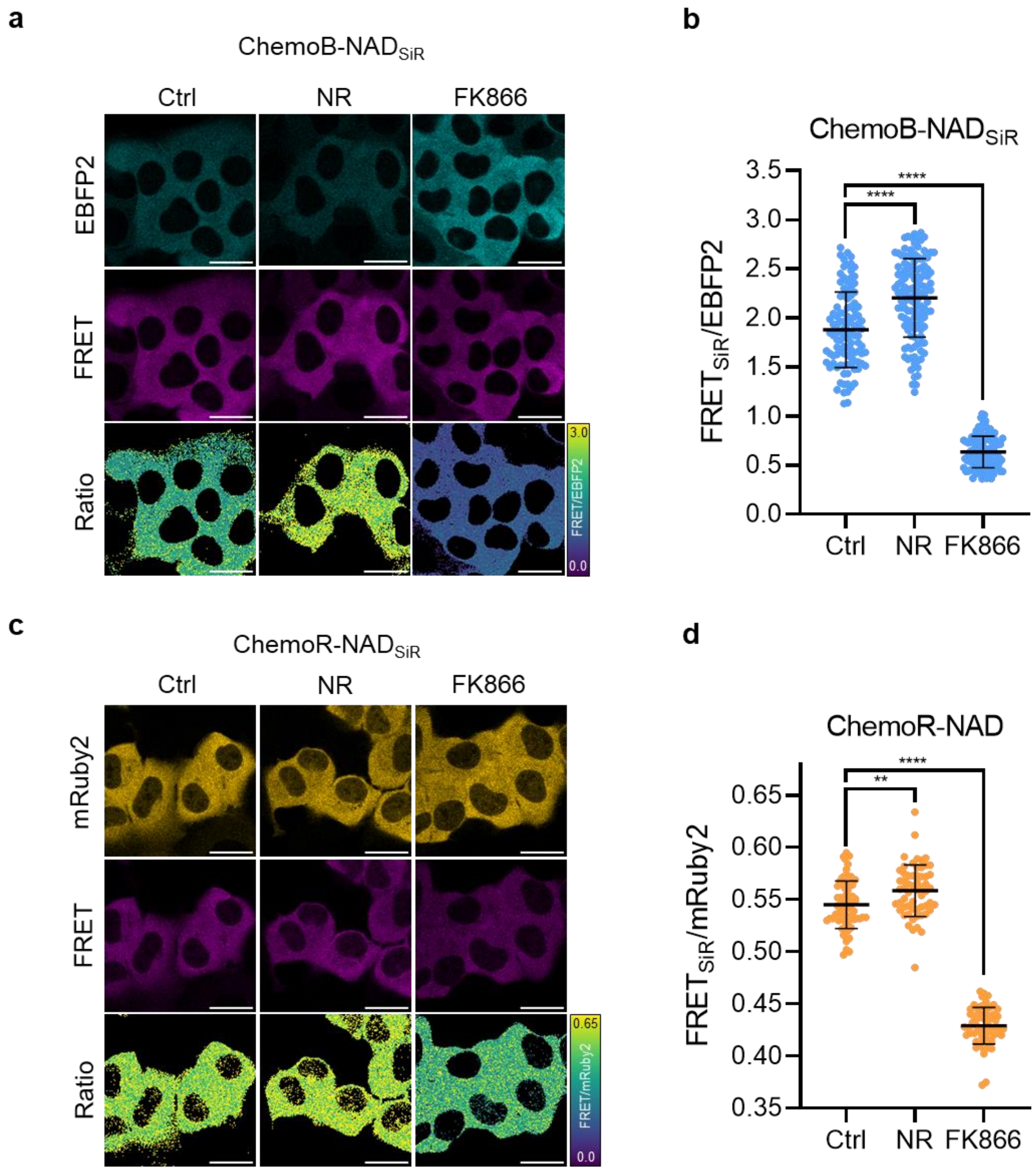

Supplementary Figure 8| Performance of ChemoB-NAD and ChemoR-NAD in U-2 OS cells.
a, **c**. Confocal images of U-2 OS cells expressing ChemoB-NAD (**a**) or ChemoR-NAD (**c**) in the cytosol labeled with SiR. Shown are the respective FP channel, FRET channel and ratio image (FRET/FP) in pseudocolor (LUT = mpl-viridis). Cells were treated for 24 h either with DMSO (Ctrl), 100 nM FK866 or 1 mM NR. All scale bars = 25 µm. **b**, **d**. FRET/FP ratios of U-2 OS cells corresponding to panels **a** and **c**, respectively. Shown are the FRET/FP values of single cells (circles) and the mean ±s.d. (black line) (ChemoB-NAD: n = 107 (ctrl), 127 (NR), 113 (FK866) cells; ChemoR-NAD, n = 66 (ctrl), 59 NR), 62 (FK866) cells; from 3 independent experiments). p-values are given based on unpaired two-tailed t-test with Welch's correction (**** p<0.0001, ** p = 0.0021).

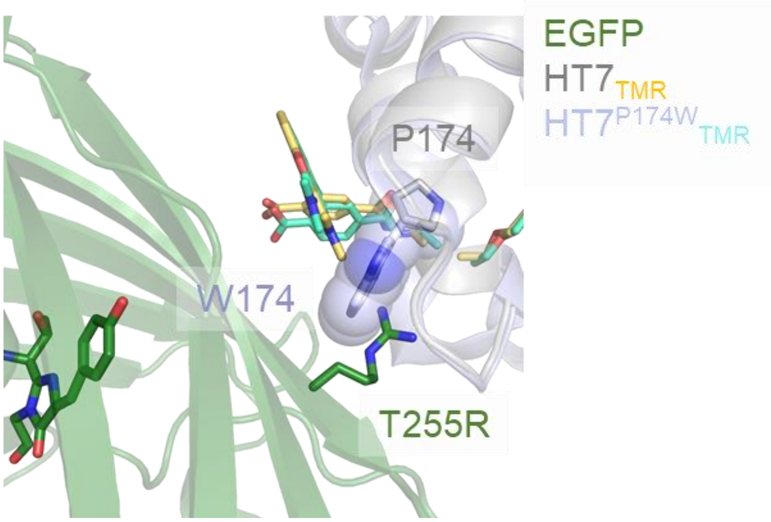

Supplementary Figure 9| Structural comparison of ChemoG and HaloTag7^{P174W}.

Zoom-in of the X-ray structure of ChemoG5$_{TMR}$ (PDB ID: 8B6T) overlayed with the X-ray structure of HaloTag7$^{P174W}_{TMR}$ (PDB ID: 6ZVV). The EGFP chromophore (green), EGFP surface residue T225R (green), HaloTag7 residues (grey or slate) and TMR (orange or cyan) are shown as sticks. The atoms of the surface residue W174 of HaloTag^{P174W} are additionally shown as spheres to visualize the steric clash with T225R of EGFP in the current conformation, suggesting the necessity of a conformational change in the closed form of the ChemoD-NAD sensor.

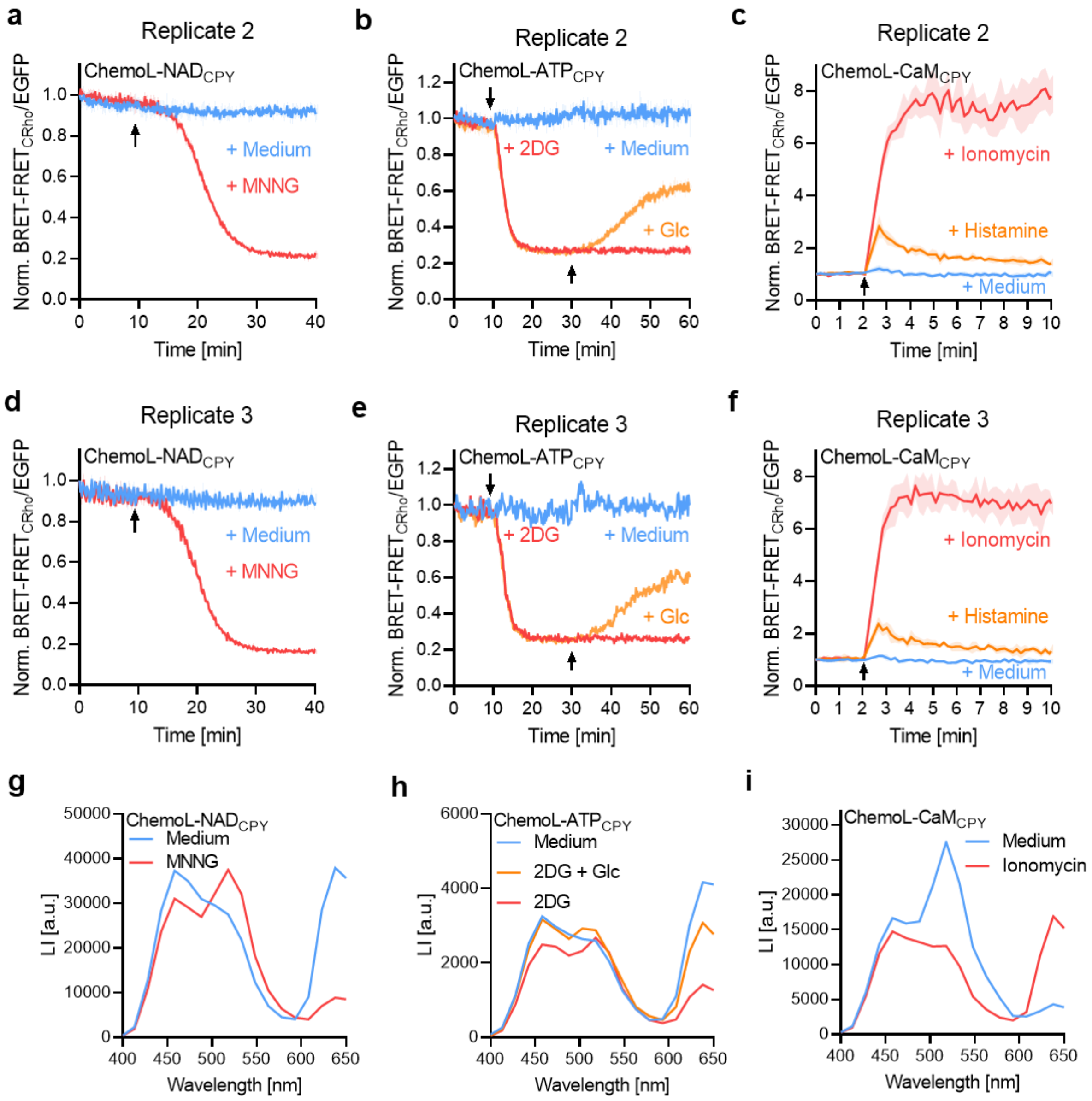

Supplementary Figure 10| ChemoL sensor performances in U-2 OS cells.

a-f. Time course measurements of ChemoL sensors expressed in U-2 OS cells (ChemoL-NAD, **a, d**) or HeLa Kyoto cells (ChemoL-ATP (**b, e**) and ChemoL-CaM (**c, f**)) upon drug treatments. Sensors were labeled with CPY. Represented are the BRET-FRET/EGFP ratios normalized to 1 at t = 0 min. Cells were untreated (+ medium) or treated with different reagents indicated with an arrow (n = 3 wells for each condition of each experiment). Represented are the mean (solid line) and the standard deviation (shade areas). The treatments are identical to time courses in **Fig. 6e-g**. **g-i**. Luminescent intensity (LI) spectra of ChemoL-NAD (**g**), ChemoL-ATP (**h**) or ChemoL-CaM (**i**) expressed in U-2 OS cells (ChemoL-NAD) or HeLa Kyoto cells (ChemoL-ATP and ChemoL-CaM). Sensors were labeled with CPY. The treatments are identical to time courses in **Fig. 6e-g**. Spectra were acquired immediately after the duration of the time courses (ChemoL-NAD = 40 min, ChemoL-ATP = 60 min, ChemoL-CaM = 10 min).

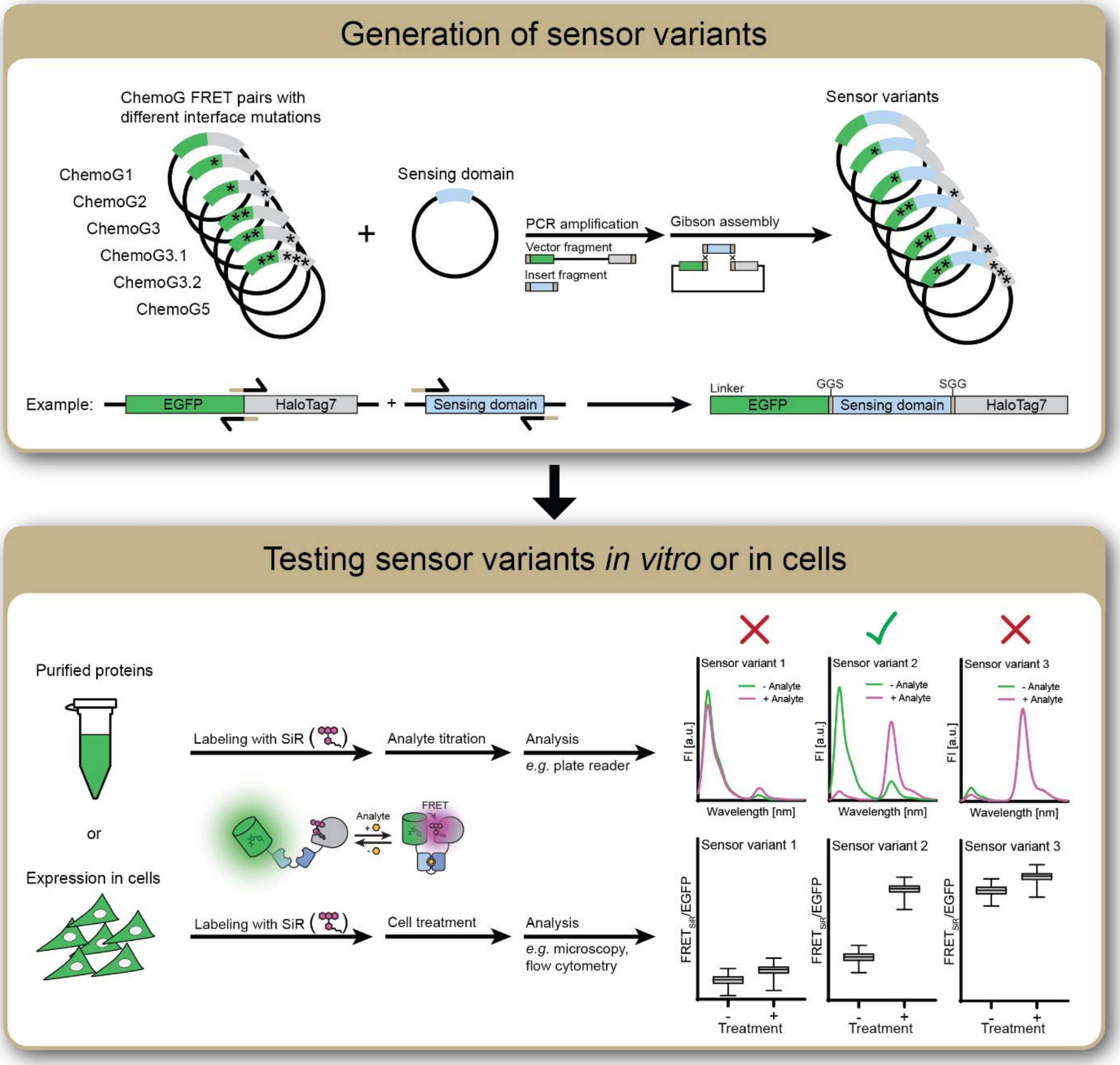

Supplementary Figure 11| Development of ChemoG biosensors.
See **Supplementary Note 1** for explanations.

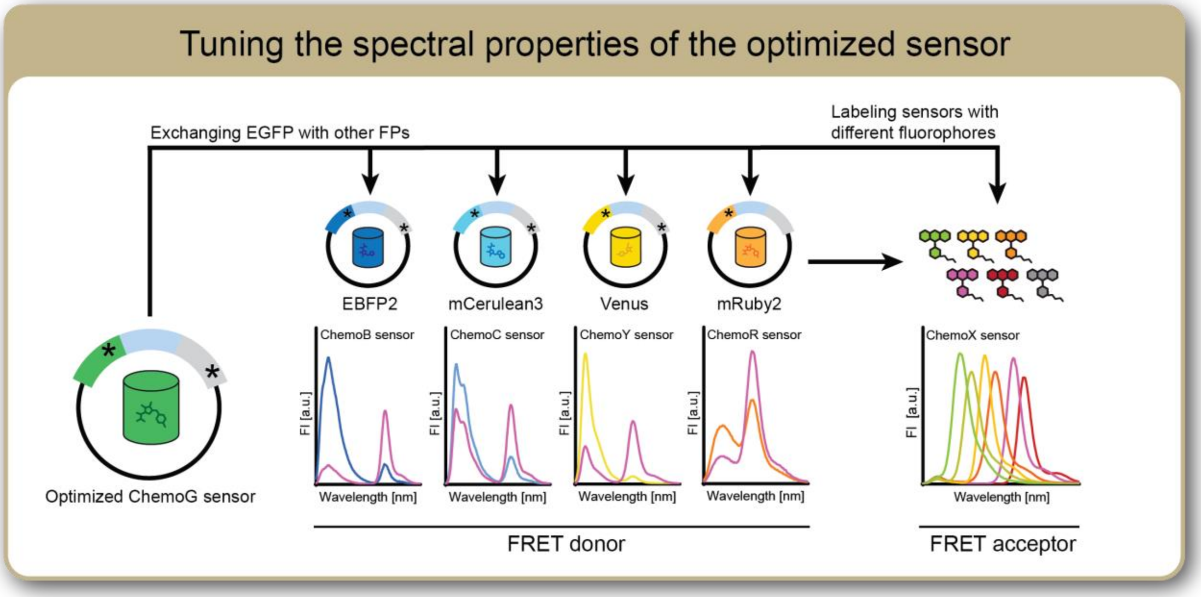

Supplementary Figure 12| Tuning the spectral properties of the optimized ChemoG sensor.
See **Supplementary Note 2** for explanations.

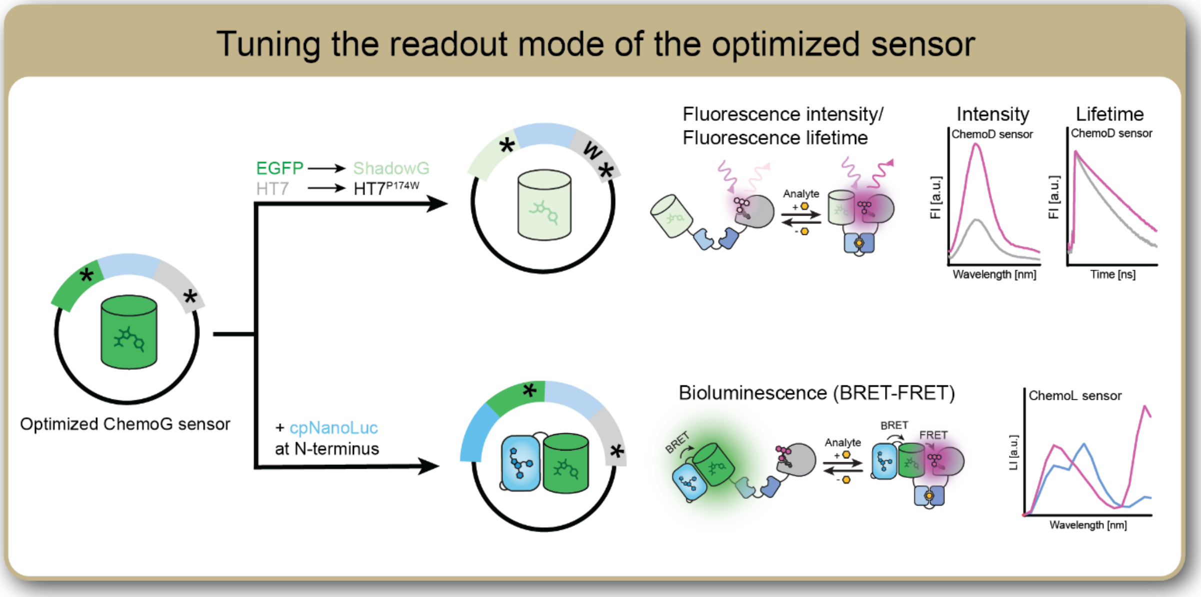

Supplementary Figure 13| Tuning the readout mode of the optimized ChemoG sensor.
See **Supplementary Note 3** for explanations.

Supplementary Tables

Supplementary Table 1| FRET efficiencies of the ChemoG interface variants.

Construct	Interface mutations		FRET ratio	FRET efficiency [%]
	EGFP	HaloTag7		
ChemoG1	-	-	2.2 ±0.1	74.8 ±0.4
ChemoG2	A206K	-	4.0 ±0.1	84.1 ±0.6
ChemoG3	A206K	L271E	8.9 ±0.1	90.9 ±0.1
ChemoG4	A206K	L271E-E143R-E147R	11.6 ±0.3	93.2 ±0.1
ChemoG5	A206K-T225R	L271E-E143R-E147R	20.3 ±0.8	95.8 ±0.1

FRET ratios (FRET/EGFP) and FRET efficiencies were determined for purified constructs labeled with SiR. Shown are the means ±s.d. (n = 3 technical replicates).

Supplementary Table 2| FRET ratios of ChemoX constructs expressed in U-2 OS cells.

Construct	Subcellular localization	Localization tag	FRET ratio	Number of cells
HT7-EGFP	-	-	0.1 ±0.05	9
ChemoG1	-	-	3.7 ±0.3	18
ChemoG2	-	-	5.7 ±1.1	15
ChemoG3	-	-	8.7 ±1.4	20
ChemoG4	-	-	13.6 ±2.2	29
ChemoG5	-	-	16.4 ±2.7	32
ChemoG5	Cytosol	NES	21.5 ±5.3	60
ChemoG5	Outer plasma membrane	$PDGFR_{tm}$	26.5 ±8.7	59
ChemoG5	Nucleus	NLS	17.8 ±3.4	51
ChemoG5	Mitochondria	Cox8	16.3 ±7.0	126
ChemoG5	Nuclear envelope	Lamin B1	15.9 ±6.5	29
ChemoB	-	-	14.6 ±3.0	20
ChemoC	-	-	14.5 ±2.6	18
ChemoY	-	-	17.5 ±5.6	24
ChemoR	-	-	14.2 ±2.5	27

FRET ratios (FRET/FP) were determined for each construct expressed in U-2 OS cells labeled with SiR. Shown are the means ±s.d.

Supplementary Table 3| FRET efficiencies of ChemoG5 labeled with different rhodamine fluorophores.

Construct	Fluorophore	Max emission [nm]	FRET ratio	FRET efficiency [%]
ChemoG5	JF_{525}	556 nm	18.0 ±1.4	94.9 ±0.3
ChemoG5	TMR	580 nm	23.6 ±2.7	96.6 ±0.3
ChemoG5	580CP	606 nm	23.8 ±2.6	96.1 ±0.5
ChemoG5	CPY	628 nm	15.8 ±1.3	94.9 ±0.4
ChemoG5	SiR	668 nm	20.2 ±0.8	95.6 ±0.1
ChemoG5	JF_{669}	686 nm	14.2 ±0.1	94.7 ±0.4

FRET/EGFP ratios and FRET efficiencies were determined for purified ChemoG5 labeled with different rhodamine fluorophores. Shown are the means ±s.d. (n = 3 technical replicates).

Supplementary Table 4| FRET efficiencies of ChemoX FRET pairs.

Construct	FP	Interface mutations		FRET ratio	FRET efficiency [%]
		XFP	HaloTag7		
ChemoB	EBFP2	N39Y-V206K-T225R	L271E-E143R-E147R	36.2 ±0.3	96.6 ±0.1
ChemoC*	mCerulean3	T225R	L271E-E143R-E147R	22.3 ±0.7	94.6 ±0.3
ChemoG5	EGFP	A206K-T225R	L271E-E143R-E147R	20.3 ±0.8	95.8 ±0.1
ChemoY	Venus	A206K-T225R	L271E-E143R-E147R	22.4 ±1.9	96.6 ±0.1
ChemoR	mScarlet	D201K	-	8.4 ±0.2	91.3 ±0.3
ChemoRuby2	mRuby2	-	-	15.0 ±0.1	91.7 ±0.2

FRET/FP ratios of purified ChemoX constructs were determined upon labeling with SiR. Shown are the means ±s.d. (n = 3 technical replicates). *mCerulean3 contains already K206, thus additional mutation at this position was not needed.

Supplementary Table 5| Summarizing characteristics of the calcium sensors.

Construct	FP	Interface mutations			C50	Max $\Delta R/R_0$	Hill slope
		# of mut.	XFP	HaloTag7			
1	EGFP	0	-	-	189 nM	22.8 ±0.3	2.2
2	EGFP	1	A206K	-	203 nM	33.3 ±0.8	1.8
3 (ChemoG-CaM)	EGFP	2	A206K	L271E	179 nM	36.1 ±1.0	2.2
4	EGFP	3	A206K	L271E-E143R-E147R	121 nM	5.2 ±0.2	1.5
5	EGFP	4	A206K-T225R	L271E-E143R-E147R	207 nM	0.8 ±0.1	1.1
ChemoB-CaM	EBFP2	2	N39Y-V206K	-	206 nM	12.7 ±0.2	1.8
ChemoC-CaM	mCerulean3	1	A206K	-	158 nM	2.3 ±0.1	3.2
ChemoY-CaM	Venus	1	A206K	-	226 nM	21.7 ±0.6	2.0
ChemoR-CaM0.1	mScarlet	1	-	-	n.d.	2.6 ±0.1	n.d
ChemoR-CaM	mRuby2	0	-	-	202 nM	3.4 ±0.1	2.7
ChemoR-CaM0.2	mRuby3	0	-	-	n.d.	2.5 ±0.1	n.d.
ChemoR-CaM0.3	mCherry	0	-	-	n.d.	2.1 ±0.1	n.d.
ChemoR-CaM0.4	mKO2	0	-	-	n.d.	1.9 ±0.1	n.d.
ChemoR-CaM0.4	TagRFP	0	-	-	n.d.	2.0 ±0.1	n.d.
YC 3.6	ECFP/Venus	-	-	-	243 nM	5.7 ±0.1	1.6

Maximum FRET/FP ratio changes (${}^{Max}\Delta R/R_0$), C50 and Hill slope were determined for purified constructs. ChemoX-based calcium sensors were labeled with SiR. Values are based on titrations performed at 37 °C. Shown are the means and for $\Delta R/R_0$ also the standard deviations (n = 3-4 technical replicates).

Supplementary Table 6| Summarizing characteristics of ChemoG-CaM labeled with different FRET acceptors.

Construct	Fluorophore	Max emission [nm]	C50	Max $\Delta R/R_0$	Hill slope
ChemoG-CaM	TMR	580 nm	66 nM	3.9 ±0.1	1.4
ChemoG-CaM	JF_{585}	610 nm	100 nM	10.5 ±0.4	1.5
ChemoG-CaM	CPY	628 nm	76 nM	8.6.0 ±0.1	2.2
ChemoG-CaM	JF_{635}	656 nm	114 nM	24.4 ±0.3	2.5
ChemoG-CaM	SiR	668 nm	179 nM	36.8 ±0.2	2.2

Maximum FRET/EGFP ratio changes ($^{Max}\Delta R/R_0$), C50 and Hill slope were determined for purified ChemoG-CaM labeled with different fluorophores. Values are based on titrations performed at 37 °C. Shown are the mean and for $\Delta R/R_0$ also the standard deviations (n = 3 technical replicates).

Supplementary Table 7| Summarizing characteristics of ATP sensors.

Construct	FP	Interface mutations		C50	Max $\Delta R/R_0$	Hill slope
		XFP	HaloTag7			
1	EGFP	A206K	-	N.D	9.9 ±0.1	N.D
2a (ChemoG-ATP)	EGFP	A206K	L271E	2.3 mM	12.1 ±0.4	1.4
2b	EGFP	A206K-T225R	-	N.D.	6.0 ±0.1	N.D
3	EGFP	A206K-T225R	L271E	N.D.	1.9 ±0.0	N.D
ChemoB-ATP	EBFP2	N39Y-V206K	L271E	2.8 mM	5.0 ±0.1	1.6
ChemoR-ATP	mRuby2	-	-	3.2 mM	0.8 ±0.1	2.0
ATeam 1.03	mseCFP/cpVenus	-	-	1.8 mM	1.4 ±0.1	1.8

Maximum FRET/FP ratio changes (${}^{Max}\Delta R/R_0$), C50 and Hill slope were determined for purified constructs. ChemoX-based ATP sensors were labeled with SiR. Values are based on titrations performed at 37 °C. Shown are the mean and for $\Delta R/R_0$ also the standard deviations (n = 3 technical replicates).

Supplementary Table 8| Summarizing characteristics of NAD$^+$ sensors.

Construct	FP	Affinity mutation *tt*LigA	Interface mutations		Fluo	C50	Max ΔR/R_0	Hill slope
			XFP	HaloTag7				
1	EGFP	-	A206K	-	TMR	38 μM	10.1 ±0.1	1.6
2	EGFP	V292A	A206K	-	TMR	75 μM	6.2 ±0.1	1.2
3	EGFP	Y226W	A206K	-	TMR	129 μM	6.3 ±0.1	1.2
4	EGFP	Y226W-V292A	A206K	-	SiR	205 μM	2.0 ±0.1	0.9
5	EGFP	Y226W-V292A	A206K-T225R	-	SiR	167 μM	18.1 ±0.3	1.0
6 (ChemoG-NAD)	EGFP	Y226W-V292A	A206K-T225R	L271E	SiR	200 μM	34.7 ±0.4	0.8
ChemoG-NAD	EGFP	Y226W-V292A	A206K-T225R	L271E	TMR	136 μM	7.5 ±0.1	1.0
ChemoG-NAD	EGFP	Y226W-V292A	A206K-T225R	L271E	JF_{585}	36 μM	18.5 ±0.1	0.9
ChemoG-NAD	EGFP	Y226W-V292A	A206K-T225R	L271E	CPY	117 μM	20.4 ±0.1	0.8
ChemoG-NAD	EGFP	Y226W-V292A	A206K-T225R	L271E	JF_{635}	52 μM	22.5 ±0.1	0.9
7	EGFP	Y226W-V292A	A206K-T225R	L271E- E143R-E147R	SiR	25 μM	32.5 ±0.3	0.8
ChemoB-NAD	EBFP2	Y226W-V292A	N39Y-A206K-T225R	L271E	SiR	103 μM	11.2 ±0.1	0.9
ChemoR-NAD	mRuby2	Y226W*	-	-	SiR	78 μM	3.0 ±0.1	1.0

Maximum FRET/FP ratio changes ($^{Max}ΔR/R_0$), C50 and Hill slope were determined for purified constructs labeled with indicated fluorophore substrates. Values are based on titrations performed at 37 °C. Shown are the mean and for $ΔR/R_0$ also the standard deviations (n = 3 technical replicates).

Supplementary Table 9| Summarizing characteristics of intensiometric NAD+ sensors.

Construct	Fluorophore	Max emission [nm]	C50	Max $\Delta FI/FI_0$	Hill slope
ChemoG-NAD	SiR	666 nm	21.0 µM	28.0 ±1.9 %	0.89
ChemoD-NAD	SiR	666 nm	32.7 µM	161.1 ±5.0 %	0.84
ChemoD-NAD	CPY	628 nm	36.8 µM	104.7 ±1.2 %	0.83
ChemoD-NAD	JF_{635}	662 nm	47.5 µM	226.6 ±4.3 %	0.59

Maximum fluorescence intensity changes ($^{Max}\Delta FI/FI_0$), C50 and Hill slopes were determined for purified constructs labeled with the indicated fluorophores. Values are based on titrations performed at 37 °C. Shown are the means and for $\Delta F/F_0$ also the standard deviations (n = 3 technical replicates).

Supplementary Table 10| Summarizing characteristics of fluorescence lifetime-based NAD+ sensors.

Construct	Fluorophore	Max emission [nm]	C50	MaxΔτ	Hill slope
ChemoG-NAD	SiR	666 nm	14.2 μM	0.53 ±0.03 ns	1.34
ChemoD-NAD	SiR	666 nm	22.4 μM	1.16 ±0.01 ns	0.99
ChemoD-NAD	CPY	628 nm	44.6 μM	1.18 ±0.01 ns	0.91
ChemoD-NAD	JF_{635}	662 nm	32.3 μM	0.77 ±0.01 ns	0.68

Maximum intensity-weighted average fluorescence lifetime changes (MaxΔτ), C50 and Hill slopes were determined for purified constructs labeled with the indicated fluorophores. Values are based on titrations performed at 37 °C. Shown are the means and for MaxΔτ also the standard deviations (n = 3 technical replicates).

Supplementary Table 11| ChemoG FRET pairs recommended for the development of ChemoG FRET biosensors.

Construct	Interface mutations		Addgene#
	EGFP	HaloTag7	
ChemoG1	-	-	193799
ChemoG2	A206K	-	193800
ChemoG3	A206K	L271E	193801
ChemoG3.1	A206K-T225R	-	193802
ChemoG3.2	A206K-T225R	L271E	193803
ChemoG5	A206K-T225R	L271E-E143R-E147R	193805

Supplementary Table 12| Chemicals and reagents used in this study.

Chemical/Reagent	Manufacturer	Catalogue number
KOD Hot Start Master Mix	Sigma-Aldrich	71842
Q5® Site-Directed Mutagenesis Kit	NEB	E0554S
QIAprep Spin Miniprep Kit	Qiagen	27106
GeneJET Endo-Free Plasmid-Maxiprep-Kit	ThermoFisher	K0861
Isopropyl-β-D-thiogalactopyranoside (IPTG)	Roth	CN084
Phenylmethylsulfonyl fluoride (PMSF)	ThermoScientific	36978
Lysozyme	ThermoScientific	89833
HisPur™ Ni-NTA Superflow Agarose	ThermoScientific	25217
4-20% Mini Protean TGX stain-free gel	Bio-Rad	568094
Amicon® Ultra 4 mL Centrifugal Filters	Merck	UFC803024 (30 kDa)<br>UFC805024 (50 kDa)
Glycerol	Merck	356350
Bovine serum albumin (BSA)	Roth	01634
4-(2-hydroxyethyl)-1-piperazineethanesulfonic acid (HEPES)	Sigma-Aldrich	H4034
Sodium chloride (NaCl)	Merck	106404
Dimethyl sulfoxide (DMSO)	Applichem	A36720100
Calcium chloride	Roth	A1191
ethylene glycol-bis(β-aminoethyl ether)-*N*,*N*,*N*′,*N*′-tetraacetic acid (EGTA)	Sigma-Aldrich	E4378
Calcium Calibration buffer Kit #1	Life technologies	C3008MP
SPG pH 4.0 - 1 M buffer	Jena Bioscience	CSS-389
SPG pH 10.0 - 1 M buffer	Jena Bioscience	CSS-390
Histamine	Sigma-Aldrich	H7250
Ionomycin	Sigma-Aldrich	I9657
cOmplete™ Protease inhibitor cocktail	Roche	11836153001
CelLytic™ M	Sigma-Aldrich	C2978
Cal520-AM	Abcam	ab171868
Digitonin 5%	ThermoScientific	BN2006
1x PBS pH 7.4	Gibco	10010015
1x HBSS with calcium and magnesium	Corning	21-023-CMR
TrypLE™ Express	Gibco	12604013
DMEM high glucose +GlutaMAX™	Gibco	31966021
DMEM high glucose, phenol red-free	Gibco	31053028
DMEM no glucose, phenol red-free	Gibco	A1443001
Sodium pyruvate (100X)	Gibco	11360070
GlutaMAX™ Supplement (100x)	Gibco	35050038
Fetal bovine serum (FBS, heat-inactivated)	Gibco	10500064
Opti-MEM™, reduced serum	Gibco	31985047
Lipofectamine 3000 Transfection Reagent	Invitrogen	L3000001
Adenosine-5′-triphosphate (ATP) magnesium salt	Sigma-Aldrich	A9187
Adenosine-5′-diphosphate (ADP) disodium salt	Sigma-Aldrich	1897
Adenosine-5′-monophosphate (AMP) sodium salt	Sigma-Aldrich	A1752
Guanosine-5′-triphosphate (GTP) sodium salt	Sigma-Aldrich	10106399001
2-Deoxy-D-glucose (2DG)	TCI Chemicals	D0051
D-glucose monohydrate	Roth	6780
Nicotinamide (NAM)	Sigma-Aldrich	72340
Nicotinamide riboside (NR)	Combi-Blocks	HB-5832
Nicotinamide mononucleotide (NMN)	Sigma-Aldrich	N3501
Nicotinamide adenine dinucleotide (NAD+)	Roche	10127965001
Nicotinamide adenine dinucleotide phosphate (NADP+)	Roth	AE13.3

Nicotinamide adenine nucleotide, reduced (NADH)	Roth	AE12.2
Nicotinic acid adenine dinucleotide (NAAD+)	Sigma-Aldrich	N4256
FK866	Selleckchem	S2799
N-methyl-*N*'-nitro-*N*-nitrosoguanidine (MNNG)	Biozol	N529925
MitoTracker™ Red FM	Invitrogen	M22425
Hoechst 33342	Invitrogen	H3570
Penicillin-Streptomycin (Pen/Strep)	Gibco	15140122
NanoBRET™ Nano-Glo Substrate	Promega	N157C
Extracellular NanoLuc® Inhibitor	Promega	N235A
Nano-Glo™ Substrate	Promega	N113B
DL-2-Amino-5-phosphonovaleric acid (APV)	SantaCruz	sc-201503
NBQX disodium salt	Sigma-Aldrich	N183
Black non-binding flat bottom 96 well plates	Perkin Elmer	6005720
Black low volume flat bottom 384 well plates	Corning	3820
White non-binding flat bottom 96 well plates	Perkin Elmer	6005290
White 96 well plate, cell culture treated	BrandTech	782090
White low volume flat bottom 384 well plates	Corning	3824
Black 96 well glass bottom imaging plate	IBL, Cellvis	P96-1.5H-N
Black 24 well glass bottom imaging plate	IBL, Cellvis	P24-1.5H-N

Supplementary Table 13| Fluorophores used in this study.

Number	Structure	Name	Ex$_{max}$/Em$_{max}$ [nm]	Source, reference
1	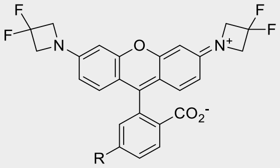	JF$_{525}$-CA	525/549	Gift from Dr. Luke Lavis, HHMI, Ashburn, VA, USA[1]
2	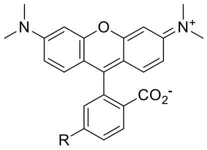	TMR-CA	548/572	Purchased from Promega, Madison, WI, USA[2]
3	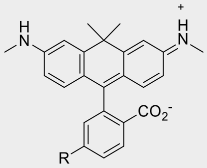	580CP-CA	582/607	Gift from Dr. Alexey N. Butkevich, MPI-MF, Heidelberg, Germany[3]
4	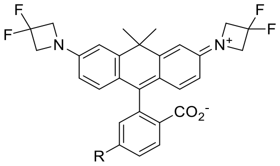	JF$_{585}$-CA	585/609	Gift from Dr. Luke Lavis, HHMI, Ashburn, VA, USA[1]
5	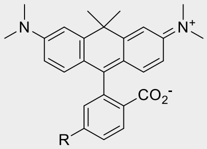	CPY-CA	606/626	Butkevich *et al*.[4]
6	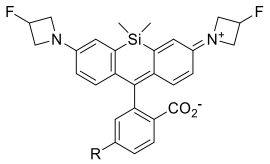	JF$_{635}$-CA	635/652	Gift from Dr. Luke Lavis, HHMI, Ashburn, VA, USA[1]
7	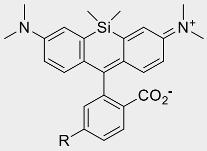	SiR-halo (=SiR-CA)	643/662	Lukinavicius *et al*.[5]
8	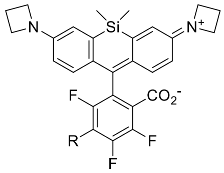	JF$_{669}$-CA	669/682	Gift from Dr. Luke Lavis, HHMI, Ashburn, VA, USA[1]
9	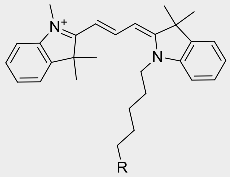	Cy3-CA	554/568	Wilhelm and Kuehn *et al*.[6]

10	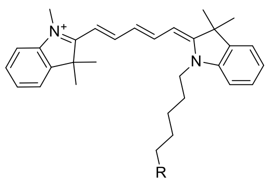	Cy5-CA	649/666	Wilhelm and Kuehn *et al*.[6]

R = 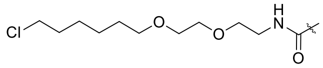 (Chloroalkane (CA))

JF = Janelia Fluor

Supplementary Table 14| Plasmids and stable cell lines used in this study.

Construct	Plasmid	Gene	Entry plasmids (Addgene#)	Addgene#	Stable cell line
pET-51b(+) HaloTag7-EGFP	pET-51b(+)	HaloTag7-EGFP	167266[6], 130706[7]	n.a.	n.a.
pET-51b(+) ChemoG1	pET-51b(+)	ChemoG1	167266[6], 130706[7]	193799	n.a.
pET-51b(+) ChemoG1^{Y39A}	pET-51b(+)	ChemoG1^{Y39A}	ChemoG1	n.a.	n.a.
pET-51b(+) ChemoG1^{K41A}	pET-51b(+)	ChemoG1^{K41A}	ChemoG1	n.a.	n.a.
pET-51b(+) ChemoG1^{F223R}	pET-51b(+)	ChemoG1^{F223R}	ChemoG1	n.a.	n.a.
pET-51b(+) ChemoG2	pET-51b(+)	ChemoG2	ChemoG1	193800	n.a.
pET-51b(+) ChemoG3	pET-51b(+)	ChemoG3	ChemoG2	193801	n.a.
pET-51b(+) ChemoG3.1	pET-51b(+)	ChemoG3	ChemoG2	193802	n.a.
pET-51b(+) ChemoG3.2	pET-51b(+)	ChemoG3	ChemoG2	193803	n.a.
pET-51b(+) ChemoG4	pET-51b(+)	ChemoG4	ChemoG3	193804	n.a.
pET-51b(+) ChemoG5	pET-51b(+)	ChemoG5	ChemoG4	193805	n.a.
pET-51b(+) ChemoB	pET-51b(+)	ChemoB	54572[8]	n.a.	n.a.
pET-51b(+) ChemoC	pET-51b(+)	ChemoC	48203[9]	n.a.	n.a.
pET-51b(+) ChemoY	pET-51b(+)	ChemoY	39813[10]	n.a.	n.a.
pET-51b(+) ChemoR	pET-51b(+)	ChemoR	85042[11]	n.a.	n.a.
pCDNA5/FRT-ChemoG1	pCDNA5/FRT	ChemoG1	167266[6], 130706[7]	193806	n.a.
pCDNA5/FRT-ChemoG2	pCDNA5/FRT	ChemoG2	ChemoG1	n.a.	n.a.
pCDNA5/FRT-ChemoG3	pCDNA5/FRT	ChemoG3	ChemoG2	n.a.	n.a.
pCDNA5/FRT-ChemoG4	pCDNA5/FRT	ChemoG4	ChemoG3	n.a.	n.a.
pCDNA5/FRT-ChemoG5	pCDNA5/FRT	ChemoG5	ChemoG4	193807	n.a.
pCDNA5/FRT-ChemoB	pCDNA5/FRT	ChemoB	54572[8]	193808	n.a.
pCDNA5/FRT-ChemoC	pCDNA5/FRT	ChemoC	48203[9]	193809	n.a.
pCDNA5/FRT-ChemoY	pCDNA5/FRT	ChemoY	39813[10]	193810	n.a.
pCDNA5/FRT-ChemoR	pCDNA5/FRT	ChemoR	85042[11]	193811	n.a.
pCDNA5/FRT-NES-ChemoG5	pCDNA5/FRT	NES-ChemoG5	ChemoG5	n.a.	n.a.
pCDNA5/FRT-ChemoG5-PDGFR$_{tm}$	pCDNA5/FRT	ChemoG5-PDGFR$_{tm}$	ChemoG5, in-house plasmid[12]	n.a.	n.a.
pCDNA5/FRT-ChemoG5-NLS3x	pCDNA5/FRT	ChemoG5-NLS3x	ChemoG5	n.a.	n.a.
pCDNA5/FRT-[2xCox8]-ChemoG5	pCDNA5/FRT	[2xCox8]-ChemoG5	ChemoG5, 113916[13]	n.a.	n.a.
pCDNA5/FRT-ChemoG5-LaminB1	pCDNA5/FRT	ChemoG5-LaminB1	55069	n.a.	n.a.
pET-51b(+) EGFP-CaM-P30-M13-HaloTag7	pET-51b(+)	EGFP-CaM-P30-M13-HaloTag7	40755[14]	n.a.	n.a.
pET-51b(+) EGFP^{A206K}-CaM-P30-M13-HaloTag7	pET-51b(+)	EGFP^{A206K}-CaM-P30-M13-HaloTag7	40755[14]	n.a.	n.a.
pET-51b(+) ChemoG-CaM	pET-51b(+)	ChemoG-CaM	40755[14]	n.a.	n.a.
pET-51b(+) EGFP^{A206K}-CaM-P30-M13-HaloTag7$^{E143R-E147R-L271E}$	pET-51b(+)	EGFP^{A206K}-CaM-P30-M13-HaloTag7$^{E143R-E147R-L271E}$	40755[14]	n.a.	n.a.
pET-51b(+) EGFP$^{A206K-T225R}$-CaM-P30-M13-HaloTag7$^{E143R-E147R-L271E}$	pET-51b(+)	EGFP$^{A206K-T225R}$-CaM-P30-M13-HaloTag7$^{E143R-E147R-L271E}$	40755[14]	n.a.	n.a.
pET-51b(+) ChemoB-CaM	pET-51b(+)	ChemoB-CaM	40755[14]	193812	n.a.
pET-51b(+) ChemoC-CaM	pET-51b(+)	ChemoC-CaM	40755[14]	193813	n.a.
pET-51b(+) ChemoY-CaM	pET-51b(+)	ChemoY-CaM	40755[14]	193814	n.a.

pET-51b(+) ChemoR-CaM	pET-51b(+)	ChemoR-CaM	40755[14]	193815	n.a.
pET-51b(+) YC 3.6	pET-51b(+)	YC 3.6	51966[15]	n.a.	n.a.
pCDNA5/FRT-ChemoG-CaM	pCDNA5/FRT	ChemoG-CaM	40755[14]	193816	n.a.
pGP-AAV2-hSyn1-NES-ChemoG-CaM	pGP-AAV2	NES-ChemoG-CaM	101061[16]	193817	n.a.
pET-51b(+) EGFP^{A206K}-F_OF_1-HT7	pET-51b(+)	EGFP^{A206K}-F_OF_1-HT7	51958[17]	n.a.	n.a.
pET-51b(+) ChemoG-ATP	pET-51b(+)	ChemoG-ATP	51958[17]	n.a.	U-2 OS Flp-In T-REx
pET-51b(+) EGFP$^{A206K-T225R}$-F_OF_1-HT7	pET-51b(+)	EGFP$^{A206K-T225R}$-F_OF_1-HT7	51958[17]	n.a.	n.a.
pET-51b(+) EGFP$^{A206K-T225R}$-F_OF_1-HT7^{L271E}	pET-51b(+)	EGFP$^{A206K-T225R}$-F_OF_1-HT7^{L271E}	51958[17]	n.a.	n.a.
pET-51b(+) ChemoB-ATP	pET-51b(+)	ChemoB-ATP	51958[17]	n.a.	n.a.
pET-51b(+) ChemoR-ATP	pET-51b(+)	ChemoR-ATP	51958[17]	n.a.	n.a.
pET-51b(+) ATeam 1.03	pET-51b(+)	ATeam 1.03	51958[17]	n.a.	n.a.
pCDNA5/FRT-ChemoG-ATP	pCDNA5/FRT	ChemoG-ATP	51958[17]	193818	n.a.
pCDNA5/FRT-ChemoB-ATP	pCDNA5/FRT	ChemoB-ATP	51958[17]	193819	n.a.
pCDNA5/FRT-ChemoR-ATP	pCDNA5/FRT	ChemoR-ATP	51958[17]	193820	n.a.
pCDNA5/FRT/TO-ATeam 1.03	pCDNA5/FRT	ATeam 1.03	51958[17]	n.a.	U-2 OS Flp-In T-REx
pET-51b(+) EGFP^{A206K}-ttLigA$^{K118L-D289N}$-HT7	pET-51b(+)	EGFP^{A206K}-ttLigA$^{K118L-D289N}$-HT7	-	n.a.	n.a.
pET-51b(+) EGFP^{A206K}-ttLigA$^{K118L-D289N-V292A}$-HT7	pET-51b(+)	EGFP^{A206K}-ttLigA$^{K118L-D289N-V292A}$-HT7	-	n.a.	n.a.
pET-51b(+) EGFP^{A206K}-ttLigA$^{K118L-Y226W-D289N-V292A}$-HT7	pET-51b(+)	EGFP^{A206K}-ttLigA$^{K118L-Y226W-D289N-V292A}$-HT7	-	n.a.	n.a.
pET-51b(+) EGFP$^{A206K-T225R}$-ttLigA$^{K118L-Y226W-D289N-V292A}$-HT7	pET-51b(+)	EGFP$^{A206K-T225R}$-ttLigA$^{K118L-Y226W-D289N-V292A}$-HT7	-	n.a.	n.a.
pET-51b(+) ChemoG-NAD	pET-51b(+)	ChemoG-NAD	-	n.a.	n.a.
pET-51b(+) EGFP$^{A206K-T225R}$-ttLigA$^{K118L-Y226W-D289N-V292A}$-HT7$^{E143R-E147R-L271E}$	pET-51b(+)	EGFP$^{A206K-T225R}$-ttLigA$^{K118L-Y226W-D289N-V292A}$-HT7$^{E143R-E147R-L271E}$	-	n.a.	n.a.
pET-51b(+) ChemoB-NAD	pET-51b(+)	ChemoB-NAD	-	n.a.	n.a.
pET-51b(+) ChemoR-NAD	pET-51b(+)	ChemoR-NAD	-	n.a.	n.a.
pCDNA5/FRT-ChemoG-NAD	pCDNA5/FRT	ChemoG-NAD	-	193821	U-2 OS Flp-In T-REx
pCDNA5/FRT-ChemoG-NAD-NLS3x	pCDNA5/FRT	ChemoG-NAD-NLS3x	-	193822	U-2 OS Flp-In T-REx
pCDNA5/FRT/TO-[4xCox8]-ChemoG-NAD	pCDNA5/FRT/TO	[4xCox8]-ChemoG-NAD	-	193823	U-2 OS Flp-In T-REx
pCDNA5/FRT-ChemoB-NAD	pCDNA5/FRT	ChemoB-NAD	-	193824	U-2 OS Flp-In T-REx
pCDNA5/FRT-ChemoB-NAD-NLS3x	pCDNA5/FRT	ChemoB-NAD-NLS3x	-	n.a.	n.a.
pCDNA5/FRT-ChemoR-NAD	pCDNA5/FRT	ChemoR-NAD	-	193825	U-2 OS Flp-In T-REx
pCDNA5/FRT/TO-ChemoB-NAD[hsOpti]-NLS3x-T2A-[4xCox8]-ChemoG-NAD[zfOpti]	pCDNA5/FRT/TO	ChemoB-NAD[hsOpti]-NLS3x-T2A-4xCox8a-ChemoG-NAD[zfOpti]	-	n.a.	n.a.
pET-51b(+) ChemoD-NAD	pET-51b(+)	ChemoD-NAD	104620[18]	n.a.	n.a.
pCDNA5/FRT-ChemoD-NAD	pCDNA5/FRT	ChemoD-NAD	104620[18]	193826	U-2 OS Flp-In T-REx
pET-51b(+) ChemoL-NAD	pET-51b(+)	ChemoL-NAD	117909[19]	n.a.	U-2 OS Flp-In T-REx
pET-51b(+) ChemoL-CaM	pET-51b(+)	ChemoL-CaM	117909[19]	n.a.	n.a.
pET-51b(+) ChemoL-ATP	pET-51b(+)	ChemoL-ATP	117909[19]	n.a.	n.a.
pCDNA5/FRT-ChemoL-NAD	pCDNA5/FRT	ChemoL-NAD	117909[19]	193827	n.a.
pCDNA5/FRT-ChemoL-NAD-NLS3x	pCDNA5/FRT	ChemoL-NAD-NLS3x	117909[19]	193828	n.a.
pCDNA5/FRT-[4xCox8]-ChemoL-NAD	pCDNA5/FRT	[4xCox8]-ChemoL-NAD	117909[19]	193829	n.a.
pCDNA5/FRT-ChemoL-CaM	pCDNA5/FRT	ChemoL-CaM	117909[19]	193830	n.a.
pCDNA5/FRT-ChemoL-ATP	pCDNA5/FRT	ChemoL-ATP	117909[19]	193831	n.a.
pET-51b(+) His-TEV-ChemoG1	pET-51b(+)	ChemoG1	167266[6]	n.a.	n.a.
pET-51b(+) His-TEV-ChemoG5	pET-51b(+)	ChemoG5	167266[6]	n.a.	n.a.
pET-51b(+) His-TEV-HaloTag7	pET-51b(+)	HT7	167266[6]		n.a.

n.a. = not available.

Supplementary Table 15| Data collection and refinement statistics.

	HaloTag7-Cy3 8B6R	ChemoG1-TMR 8B6S	ChemoG5-TMR 8B6T
**Data collection**			
Space group	$P4_22_12$	$P1$	$P12_11$
Unit-cell parameters			
a, b, c (Å)	112.56, 112.56, 44.33	46.19, 63.71, 89.42	46.60, 64.04, 172.95
α, β, γ (°)	90.00, 90.00, 90.00	93.56, 91.02, 90.85	90.00, 97.67, 90.00
Radiation source	PXII-X10SA, SLS	PXII-X10SA, SLS	PXII-X10SA, SLS
Wavelength (Å)	0.99988	0.99996	0.99992
Temperature (K)	100	100	100
Resolution range (Å)	50-1.50 (1.60-1.50)	50-1.80 (1.90-1.80)	50-2.00 (2.10-2.00)
No. of observed reflections	341056 (60343)	182229 (26711)	216345 (30251)
No. of unique reflections	46121 (7965)	89852 (13310)	66470 (9089)
Multiplicity	7.4 (7.6)	2.0 (2.0)	3.3 (3.3)
Completeness (%)	99.9 (99.9)	95.3 (94.3)	97.0 (97.8)
R_{merge} (%)	6.8 (65.7)	4.1 (40.0)	8.6 (41.0)
$\langle I/\sigma(I) \rangle$	18.2 (3.4)	12.0 (2.1)	8.5 (3.4)
$CC_{1/2}$ (%)[#]	99.9 (90.2)	99.8 (75.2)	99.5 (87.4)
**Refinement**			
Molecules per a.u.	1	2	2
No. of reflections	46120	89842	66470
No. of reflections in test set	2306	4492	3399
Resolution range (Å)	41.25-1.50	46.18-1.80	46.18-2.00
No. of non-hydrogen atoms			
Protein	2365	8273	8276
Ligand/ion	72	146	134
Water	297	460	308
Total	2734	8879	8718
R (%)	16.20	17.28	22.06
R_{free} (%)	19.19	20.08	24.51
RMS deviations from ideal			
bonds (Å)	0.013	0.007	0.002
angles (°)	1.229	1.094	0.779
B-factors (Å^2)			
Protein	14.80	26.03	20.62
Ligand/ion	23.87	22.14	17.73
Water	24.40	29.14	19.67
Average	16.08	26.13	20.54
Wilson B (Å^2)	14.42	24.95	22.88
Ramachandran statistics (%)			
favored regions	95.9	97.5	96.8
allowed regions	4.1	2.5	3.2
disallowed regions	0	0	0
Clashscore	1.04	1.33	3.03

[#]as implemented in XDS[20]. Values in parentheses are for the highest resolution shell.

Supplementary Table 16| Spectral settings for fluorescence spectroscopy measurements.

Chromophore/fluorophore	Max. emission wavelength	Excitation wavelength used	Emission wavelength range measured
EBFP2	446 nm	360 nm	400-800 nm
mCerulean3	474 nm	400 nm	440-800 nm
EGFP	510 nm	440 nm	480-800 nm
Venus	528 nm	460 nm	494-800 nm
mKO2	566 nm	510 nm	550-800 nm
TagRFP	584 nm	510 nm	550-800 nm
mRuby2	594 nm	510 nm	550-800 nm
mRuby3	594 nm	510 nm	550-800 nm
mScarlet	594 nm	520 nm	560-800 nm
mCherry	610 nm	530 nm	570-800 nm
JF_{525}	554 nm	-	-
TMR	576 nm	-	-
Cy3	576 nm	-	-
580CP	606 nm	-	-
JF_{585}	612 nm	-	-
CPY	628 nm	580 nm	610-750 nm
JF_{635}	662 nm	610 nm	640-750 nm
Cy5	664 nm	-	-
SiR	666 nm	610 nm	640-750 nm
JF_{669}	688 nm	-	-
YC 3.6	474/528 nm	400 nm	440-650 nm
ATeam 1.03	474/528 nm	400 nm	440-650 nm

Supplementary Table 17| Analyte concentration ranges used for sensor titrations.

Experiment	Analyte	10x concentration (range)	Final 1x concentration (range)
ChemoX-CaM titration with free Ca^{2+} (Fig. 2c, d, ED3d, g, j)	Free Ca^{2+}	-	10 nM – 39 μM*
ChemoX-CaM response to free Ca^{2+} at different pH (ED3k)	$CaCl_2$	20 mM	2 mM
	EGTA	20 mM	2 mM
RFP-based calcium sensor responses to free Ca^{2+} (Fig. S5)	$CaCl_2$	20 mM	2 mM
	EGTA	20 mM	2 mM
ChemoX-ATP titration with ATP or structurally similar analytes (Fig. 3c, ED5c)	ATP	0.1 - 100 mM	0.01 - 10 mM
	ADP	0.1 - 100 mM	0.01 - 10 mM
	AMP	0.1 - 100 mM	0.01 - 10 mM
	GTP	0.1 - 100 mM	0.01 - 10 mM
ChemoX-NAD titration with NAD^+ or structurally similar analytes (Fig. 4c, d, ED6e)	NAD^+	10 nM – 100 mM	1 nM – 10 mM
	NAM	10 nM – 100 mM	1 nM – 10 mM
	NR	10 nM – 100 mM	1 nM – 10 mM
	NMN	10 nM – 100 mM	1 nM – 10 mM
	NADH	10 nM – 100 mM	1 nM – 10 mM
	$NADP^+$	10 nM – 100 mM	1 nM – 10 mM
	$NAAD^+$	10 nM – 100 mM	1 nM – 10 mM
	ATP	10 nM – 100 mM	1 nM – 10 mM
	ADP	10 nM – 100 mM	1 nM – 10 mM
ChemoX-NAD titration with NAD^+ in presence of structurally similar analytes (Fig. ED6f, g)	NAD^+ (titration)	10 nM – 100 mM	1 nM – 10 mM
	NAM (constant)	10 mM	1 mM
	NR (constant)	1 mM	0.1 mM
	NMN (constant)	1 mM	0.1 mM
	NADH (constant)	1 mM	0.1 mM
	$NAAD^+$ (constant)	1 mM	0.1 mM
	$NADP^+$ (constant)	1 mM	0.1 mM
	ATP (constant)	10 mM	1 mM
	ADP (constant)	10 mM	1 mM
	AMP (constant)	10 mM	1 mM
ChemoD-NAD titration with NAD^+ (intensiometric, Fig. 5c, ED9b, d, e)	NAD^+	100 nM – 100 mM	10 nM – 10 mM
ChemoD-NAD titration with NAD^+ (fluorescence lifetime, Fig. 5f, ED9f-i)	NAD^+	1 μM – 100 mM	100 nM – 10 mM
ChemoL-NAD titration with NAD^+ (Fig. 6c)	NAD^+	100 nM – 100 mM	10 nM – 10 mM
ChemoL-CaM titration with free Ca^{2+} (ED10d)	Free Ca^{2+}	-	50 nM – 39 μM*
ChemoL-ATP titration with ATP (ED10f)	NAD^+	100 nM – 100 mM	10 nM – 10 mM

*For titrations of calcium sensors, special calcium buffers with defined concentrations of free Ca^{2+} were prepared (see Analyte titrations of biosensors below for details).

Supplementary Table 18| Settings for confocal and widefield fluorescence microscopy.

Figure	Construct	Label	Microscope	Objective	Excitation [nm]	Emission [nm]	Pixel dwell time [µs]	Size [pixels]	Z size [µm]
1f	ChemoG5-NLS	-	Confocal	40x/1.10 water	480	490-540	3.16	512x512	5
		TMR			480	490-540/550-600	3.16	512x512	5
		CPY			480	490-540/620-670	3.16	512x512	5
		SiR			480	490-540/650-700	3.16	512x512	5
1h	ChemoB	SiR	Confocal	40x/1.10 water	405	420-470/650-700	3.16	512x512	5
	ChemoC	SiR			405	460-500/650-700	3.16	512x512	5
	ChemoG5	SiR			480	490-540/650-700	3.16	512x512	5
	ChemoY	SiR			505	515-565/650-700	3.16	512x512	5
	ChemoR	SiR			550	570-620/650-700	3.16	512x512	5
2e	ChemoG-CaM	SiR	Widefield	20x/0.80 dry	470*	525/50, 700/75**	n.d.	512x512	2
2g	ChemoG-CaM	SiR	Widefield	20x/0.80 dry	470*	525/50, 700/75**	n.d.	256x256	0
3d	ChemoG-ATP	SiR	Confocal	40x/1.10 water	480	490-540/650-700	3.16	512x512	5
3f	ChemoB-ATP	SiR	Confocal	40x/1.10 water	405	420-470/650-700	3.16	512x512	5
	ChemoG-ATP	SiR			480	490-540/650-700	3.16	512x512	5
	ChemoR-ATP	SiR			550	570-620/650-700	3.16	512x512	5
	ATeam 1.03	-			405	460-500/520-560	3.16	512x512	5
4e	ChemoG-NAD	SiR	Confocal	40x/1.10 water	480	490-540/650-700	3.16	512x512	0
4g	ChemoB-NAD-cyto	SiR	Confocal	40x/1.10 water	405	420-460/650-700	3.84	2048x2048	0
	ChemoG-NAD-mito	SiR			480	490-540/650-700	3.84	2048x2048	0
4h	ChemoB-NAD-cyto	SiR	Confocal	40x/1.10 water	405	420-460/650-700	3.16	512x512	4
	ChemoG-NAD-mito	SiR			480	490-540/650-700	3.16	512x512	4
5f	ChemoD-NAD	CPY	Confocal	40x/1.10 water	610	620-670	1.75	512x512	0
		JF_{635}			640	650-700	1.75	512x512	0
		SiR			625	635-685	1.75	512x512	0
5g/h	ChemoD-NAD	CPY	Confocal	40x/1.10 water	610	620-670	7.69	512x512	0
S4a	HT-EGFP	SiR	Confocal	40x/1.10 water	480	490-540/650-700	3.16	512.x512	5
	ChemoG1-5	SiR			480	490-540/650-700	3.16	512.x512	5
	ChemoG5-NES	SiR			480	490-540/650-700	3.16	512.x512	5
	$ChemoG5\text{-}PDGFR_{tm}$	SiR			480	490-540/650-700	3.16	512.x512	0
	ChemoG5-NLS	SiR			480	490-540/650-700	3.16	512.x512	5
	ChemoG5-Cox8	SiR			480	490-540/650-700	3.16	512.x512	0
	ChemoG5-LaminB1	SiR			480	490-540/650-700	3.16	512.x512	0
ED7a	ChemoG-NAD-NLS	SiR	Confocal	40x/1.10 water	480	490/540/650-700	3.16	512x512	0
ED7d	ChemoG-NAD-mito	SiR	Confocal	40x/1.10 water	480	490/540/650-700	3.16	512x512	0
S8a	ChemoB-NAD	SiR	Confocal	40x/1.10 water	405	420-470/650/700	3.16	512x512	0

S8c	ChemoR-NAD	SiR	Confocal	40x/1.10 water	550	570-620/650-700	3.16	512x512	0
ED8a	ChemoB-NAD-cyto	SiR	Confocal	40x/1.10 water	405	420-460/650-700	3.16	512x512	4
	ChemoG-NAD-mito	SiR			480	490-540/650-700	3.16	512x512	4
ED8b	ChemoB-NAD-NLS	SiR	Confocal	40x/1.10 water	405	420-460/650-700	3.16	512x512	0
	ChemoG-NAD-mito	SiR			480	490-540/650-700	3.16	512x512	0
ED8c	ChemoB-NAD-NLS	SiR	Confocal	40x/1.10 water	405	420-460/650-700	3.16	512x512	4
	ChemoG-NAD-mito	SiR			480	490-540/650-700	3.16	512x512	4
ED8d	ChemoB-NAD-NLS	SiR	Confocal	40x/1.10 water	405	420-460/650-700	3.16	512x512	4
	ChemoG-NAD-mito	SiR			480	490-540/650-700	3.16	512x512	4
ED9f/g	ChemoG-NAD	SiR	Confocal	40x/1.10 water	640	650-700	1.75	512x512	0
	ChemoD-NAD	SiR			640	650-700	1.75	512x512	0
ED9h	ChemoD-NAD	CPY	Confocal	40x/1.10 water	610	620-670	1.75	512x512	0
ED9i	ChemoD-NAD	JF_{635}	Confocal	40x/1.10 water	620	635-685	1.75	512x512	0
ED9j/k	ChemoD-NAD	SiR	Confocal	40x/1.10 water	640	650-700	7.69	512x512	0
ED9l/m	ChemoD-NAD	JF_{635}	Confocal	40x/1.10 water	620	635-685	7.69	512x512	0

*470 nm LED was used together with a 474/24 nm bandpass filter. **525/50 nm and 700/75 nm bandpass filters were used for acquisition of EGFP and FRET(SiR) fluorescence, respectively

Supplementary Notes

Supplementary Note 1 – Development of ChemoG biosensors.

***Generation of sensor variants*.** Certain ChemoG interface mutations increase FRET to a larger extent than others. For example, the interface mutation $T225R^{EGFP}$ usually leads to a stronger FRET increase than the interface mutation $L271E^{HT7}$. This feature revealed useful to fine-tune the dynamic range of ChemoG-based sensors. For the generation of new sensors (**Fig. S21**), we recommend to try a palette of ChemoG FRET pairs with different interface mutations (**Supplementary Table 11**, available on Addgene). The sensing domain can be derived from an existing biosensor as *e.g.* ChemoG-CaM that was derived from YC 3.6[22] or a new sensing domain, preferentially exhibiting a large conformational change. To create ChemoG sensor variants, the sensing domain should be cloned between the EGFP and HaloTag7 variants (*i.e.* ChemoG FRET pairs). Using ChemoG-encoding plasmids and DNA encoding the sensing domain of interest, 6 plasmids encoding sensor variants can simply be obtained through PCR and molecular cloning (*e.g.* by Gibson assembly[23]). We recommend to use single GGS linkers connecting the ChemoG FRET pairs with the sensing domain but these can also be further engineered in a second step if necessary. The linkers can be created during the design of the primers used for the PCR amplification of the fragments. We deposited plasmids encoding ChemoG variants for protein production in *E. coli*. In case the sensor variants should be tested in mammalian cells, the vector backbone should first be exchanged.

***Testing sensor variants in vitro or in cells*.** Two options are available:

- produce the sensor variants in *E. coli*, purify them and test them *in vitro*, or
- express and test the sensor variants in mammalian cells (require extra sub-cloning, see above).

For the first option, the purified sensor variants should be labeled with an orange/red fluorophore substrate. We recommend SiR-halo or a rhodamine substrate with similar spectral properties to minimize direct excitation of the synthetic fluorophore. The labeled sensors should then be titrated with different concentrations of an analyte of interest (AOI). The sensor variant with the largest dynamic range can be identified from fluorescence emission spectra. An ideal sensor exhibits low FRET in absence and high FRET in presence of the AOI (or *vice versa*), showing a large peak inversion in each emission channel. Some noticeable fluorescence should remain in both channels for precise measurements.

For the second option, mammalian cells should be transfected with plasmids encoding the sensor variants. The transfected cells should be labeled with cell-permeable fluorophore substrates. As previously, we recommend SiR-halo. Labeled cells can subsequently be treated with reagents known to act on the biological activity of interest (*e.g.* AOI concentration change). Via fluorescence microscopy or flow cytometry, the fluorescence profile of treated and untreated cells can be compared to identify sensor variants with the largest dynamic range. Sensors presenting noticeable fluorescence signal in both channels in presence and absence of treatment should be chosen in order to ensure precise measurement. Technical details on how to conduct the different experiments can be found in the method section of the manuscript.

Supplementary Note 2 – Tuning the spectral properties of the optimized ChemoG sensor.

The spectral properties of the optimized ChemoG sensor can be tuned by exchanging the FRET donor EGFP with other fluorescent proteins and/or by using different fluorophore substrates as FRET acceptor (**Supplementary Fig. 12**). For exchanging the FRET donor, EGFP is substituted with an alternative fluorescent protein (*e.g.* EBFP2 = ChemoB) with the same interface mutations (*e.g.* $EGFP^{T225R}$ → $EBFP2^{T225R}$) via molecular cloning. As FRET donor, we recommend EGFP-derived fluorescent proteins such as EBFP2, mCerulean3 or Venus to ensure a good transferability of the interface mutations. We recommend to use FP constructs we deposited on Addgene to ensure that the adequate FP mutations are used. For red fluorescent proteins, we recommend using mRuby2 without additional mutations for biosensor design. The FRET acceptor can be readily chosen by simply labeling the ChemoX sensors with different rhodamine-based HaloTag substrates (*e.g.* JF_{525}, CPY or JF_{669}). The ChemoX sensors performance can be evaluated as explained in **Supplementary Note 1** and in the methods.

Supplementary Note 3 – Tuning the readout mode of the optimized ChemoG sensor.

The readout of ChemoG FRET sensors can be tuned by small modifications (**Supplementary Fig. 13**). Single channel fluorescence intensity and fluorescence lifetime-based ChemoD sensors are obtained by substituting EGFP with its non-fluorescent variant ShadowG[18] carrying the same interface mutation(s). Additionally, the fluorescence quenching mutation P174W should be introduced into HaloTag7. For intensiometric sensors, we recommend labeling with JF_{635} while for fluorescence lifetime imaging, CPY worked best in our hands so far. The performance of the sensors can be evaluated analogously as explained in **Supplementary Note 1** and in the methods.

To convert ChemoG FRET sensors into a bioluminescent ChemoL sensor, a circularly permuted variant of NanoLuc is fused to the N-terminus of EGFP. We recommend labeling the sensor with rhodamine fluorophore substrates whose spectral properties are compatible with the available equipment. In our case, CPY was the most red-shifted fluorophore compatible with our plate reader but we foresee no conceptual hurdle in using any rhodamine fluorophore substrate proven functional for FRET biosensing. The sensors performance can be evaluated analogously as explained in **Supplementary Note 1** and in the methods. It should be noted, that the expression of ChemoL sensors in mammalian cells, assessed by direct excitation of EGFP, was found to be substantially lower than the corresponding ChemoG FRET sensors. Since bioluminescent signals can be detected with high sensitivity, *i.e.* also for sensors with low expression levels, it is possible to acquire robust emission spectra for ChemoL biosensors. Due to the dim EGFP signal, however, it is not advisable to use ChemoL sensors for FRET applications even if this is conceptually possible.

Supplementary Note 4 – Protein sequences

Static FRET constructs

>ChemoG5
MVSKGEELFTGVVPILVELDGDVNGHKFSVSGEGEGDATYGKLTLKFICTTGKLPVPWPTLVTTLTYGVQCFSRYPD
HMKQHDFFKSAMPEGYVQERTIFFKDDGNYKTRAEVKFEGDTLVNRIELKGIDFKEDGNILGHKLEYNYNSHNVYIM
ADKQKNGIKVNFKIRHNIEDGSVQLADHYQQNTPIGDGPVLLPDNHYLSTQSKLSKDPNEKRDHMVLLEFVRAAGIT
LGMDELYKIGTGFPFDPHYVEVLGERMHYVDVGPRDGTPVLFLHGNPTSSYVWRNIIPHVAPTHRCIAPDLIGMGKS
DKPDLGYFFDDHVRFMDAFIEALGLEEVVLVIHDWGSALGFHWAKRNPERVKGIAFMEFIRPIPTWDEWPRFARRTF
QAFRTTDVGRKLIIDQNVFIEGTLPMGVVRPLTEVEMDHYREPFLNPVDREPLWRFPNELPIAGEPANIVALVEEYM
DWLHQSPVPKLLFWGTPGVLIPPAEAARLAKSLPNCKAVDIGPGENLLQEDNPDLIGSEIARWLSTLEISG

>ChemoB
MVSKGEELFTGVVPILVELDGDVNGHKFSVRGEGEGDATYGKLTLKFICTTGKLPVPWPTLVTTLSHGVQCFARYPD
HMKQHDFFKSAMPEGYVQERTIFFKDDGTYKTRAEVKFEGDTLVNRIELKGVDFKEDGNILGHKLEYNFNSHNIYIM
AVKQKNGIKVNFKIRHNVEDGSVQLADHYQQNTPIGDGPVLLPDSHYLSTQSKLSKDPNEKRDHMVLLEFRRAAGIT
LGMDELYKIGTGFPFDPHYVEVLGERMHYVDVGPRDGTPVLFLHGNPTSSYVWRNIIPHVAPTHRCIAPDLIGMGKS
DKPDLGYFFDDHVRFMDAFIEALGLEEVVLVIHDWGSALGFHWAKRNPERVKGIAFMEFIRPIPTWDEWPRFARRTF
QAFRTTDVGRKLIIDQNVFIEGTLPMGVVRPLTEVEMDHYREPFLNPVDREPLWRFPNELPIAGEPANIVALVEEYM
DWLHQSPVPKLLFWGTPGVLIPPAEAARLAKSLPNCKAVDIGPGENLLQEDNPDLIGSEIARWLSTLEISG

>ChemoC
MVSKGEELFTGVVPILVELDGDVNGHKFSVSGEGEGDATYGKLTLKFICTTGKLPVPWPTLVTTLSWGVQCFARYPD
HMKQHDFFKSAMPEGYVQERTIFFKDDGNYKTRAEVKFEGDTLVNRIELKGIDFKEDGNILGHKLEYNAIHGNVYIT
ADKQKNGIKANFGLNCNIEDGSVQLADHYQQNTPIGDGPVLLPDNHYLSTQSKLSKDPNEKRDHMVLLEFVRAAGIT
LGMDELYKIGTGFPFDPHYVEVLGERMHYVDVGPRDGTPVLFLHGNPTSSYVWRNIIPHVAPTHRCIAPDLIGMGKS
DKPDLGYFFDDHVRFMDAFIEALGLEEVVLVIHDWGSALGFHWAKRNPERVKGIAFMEFIRPIPTWDEWPRFARRTF
QAFRTTDVGRKLIIDQNVFIEGTLPMGVVRPLTEVEMDHYREPFLNPVDREPLWRFPNELPIAGEPANIVALVEEYM
DWLHQSPVPKLLFWGTPGVLIPPAEAARLAKSLPNCKAVDIGPGENLLQEDNPDLIGSEIARWLSTLEISG

>ChemoY
MVSKGEELFTGVVPILVELDGDVNGHKFSVSGEGEGDATYGKLTLKLICTTGKLPVPWPTLVTTLGYGLQCFARYPD
HMKQHDFFKSAMPEGYVQERTIFFKDDGNYKTRAEVKFEGDTLVNRIELKGIDFKEDGNILGHKLEYNYNSHNVYIT
ADKQKNGIKANFKIRHNIEDGGVQLADHYQQNTPIGDGPVLLPDNHYLSYQSKLSKDPNEKRDHMVLLEFVRAAGIT
LGMDELYKIGTGFPFDPHYVEVLGERMHYVDVGPRDGTPVLFLHGNPTSSYVWRNIIPHVAPTHRCIAPDLIGMGKS
DKPDLGYFFDDHVRFMDAFIEALGLEEVVLVIHDWGSALGFHWAKRNPERVKGIAFMEFIRPIPTWDEWPRFARRTF
QAFRTTDVGRKLIIDQNVFIEGTLPMGVVRPLTEVEMDHYREPFLNPVDREPLWRFPNELPIAGEPANIVALVEEYM
DWLHQSPVPKLLFWGTPGVLIPPAEAARLAKSLPNCKAVDIGPGENLLQEDNPDLIGSEIARWLSTLEISG

>ChemoR
MVSKGEAVIKEFMRFKVHMEGSMNGHEFEIEGEGEGRPYEGTQTAKLKVTKGGPLPFSWDILSPQFMYGSRAFTKHP
ADIPDYYKQSFPEGFKWERVMNFEDGGAVTVTQDTSLEDGTLIYKVKLRGTNFPPDGPVMQKKTMGWEASTERLYPE
DGVLKGDIKMALRLKDGGRYLADFKTTYKAKKPVQMPGAYNVDRKLKITSHNEDYTVVEQYERSEGRHSTGGMDELY
KIGTGFPFDPHYVEVLGERMHYVDVGPRDGTPVLFLHGNPTSSYVWRNIIPHVAPTHRCIAPDLIGMGKSDKPDLGY
FFDDHVRFMDAFIEALGLEEVVLVIHDWGSALGFHWAKRNPERVKGIAFMEFIRPIPTWDEWPEFARETFQAFRTTD
VGRKLIIDQNVFIEGTLPMGVVRPLTEVEMDHYREPFLNPVDREPLWRFPNELPIAGEPANIVALVEEYMDWLHQSP
VPKLLFWGTPGVLIPPAEAARLAKSLPNCKAVDIGPGLNLLQEDNPDLIGSEIARWLSTLEISG

EGFP, EBFP2, mCerulean3, Venus, mScarlet
HaloTag7
Interface mutations (XFP^{A206K}, XFP^{T225R}, HT7^{E143R}, HT7^{E147R}, HT7^{L271E}, mScarlet^{D201K}, EBFP2^{N39Y})

Calcium sensors

>ChemoG-CaM
MVSKGEELFTGVVPILVELDGDVNGHKFSVSGEGEGDATYGKLTLKFICTTGKLPVPWPTLVTTLTYGVQCFSRYPD
HMKQHDFFKSAMPEGYVQERTIFFKDDGNYKTRAEVKFEGDTLVNRIELKGIDFKEDGNILGHKLEYNYNSHNVYIM
ADKQKNGIKVNFKIRHNIEDGSVQLADHYQQNTPIGDGPVLLPDNHYLSTQSKLSKDPNEKRDHMVLLEFVTAAGIT
GGTLPDQLTEEQIAEFKEAFSLFDKDGDGTITTKELGTVMRSLGQNPTEAELQDMINEVDADGDGTIDFPEFLTMMA
RKMKDTDSEEEIREAFRVFDKDGNGYISAAELRHVMTNLGEKLTDEEVDEMIREADIDGDGQVNYEEFVVMMTAKEF
PPPPPPPPPPPPPPPPPPPPPPPPPPPPPPGGSMVDSSRRKFNKTGKALRAIGRLSSLESGGIGTGFPFDPHYVEVL
GERMHYVDVGPRDGTPVLFLHGNPTSSYVWRNIIPHVAPTHRCIAPDLIGMGKSDKPDLGYFFDDHVRFMDAFIEAL
GLEEVVLVIHDWGSALGFHWAKRNPERVKGIAFMEFIRPIPTWDEWPEFARETFQAFRTTDVGRKLIIDQNVFIEGT
LPMGVVRPLTEVEMDHYREPFLNPVDREPLWRFPNELPIAGEPANIVALVEEYMDWLHQSPVPKLLFWGTPGVLIPP
AEAARLAKSLPNCKAVDIGPGENLLQEDNPDLIGSEIARWLSTLEISG

>ChemoB-CaM
MVSKGEELFTGVVPILVELDGDVNGHKFSVRGEGEGDATYGKLTLKFICTTGKLPVPWPTLVTTLSHGVQCFARYPD
HMKQHDFFKSAMPEGYVQERTIFFKDDGTYKTRAEVKFEGDTLVNRIELKGVDFKEDGNILGHKLEYNFNSHNIYIM
AVKQKNGIKVNFKIRHNVEDGSVQLADHYQQNTPIGDGPVLLPDSHYLSTQSKLSKDPNEKRDHMVLLEFRTAAGIT
GGTLPDQLTEEQIAEFKEAFSLFDKDGDGTITTKELGTVMRSLGQNPTEAELQDMINEVDADGDGTIDFPEFLTMMA
RKMKDTDSEEEIREAFRVFDKDGNGYISAAELRHVMTNLGEKLTDEEVDEMIREADIDGDGQVNYEEFVVMMTAKEF
PPPPPPPPPPPPPPPPPPPPPPPPPPPPPPGGSMVDSSRRKFNKTGKALRAIGRLSSLESGGIGTGFPFDPHYVEVL
GERMHYVDVGPRDGTPVLFLHGNPTSSYVWRNIIPHVAPTHRCIAPDLIGMGKSDKPDLGYFFDDHVRFMDAFIEAL
GLEEVVLVIHDWGSALGFHWAKRNPERVKGIAFMEFIRPIPTWDEWPEFARETFQAFRTTDVGRKLIIDQNVFIEGT
LPMGVVRPLTEVEMDHYREPFLNPVDREPLWRFPNELPIAGEPANIVALVEEYMDWLHQSPVPKLLFWGTPGVLIPP
AEAARLAKSLPNCKAVDIGPGENLLQEDNPDLIGSEIARWLSTLEISG

>ChemoC-CaM
MVSKGEELFTGVVPILVELDGDVNGHKFSVSGEGEGDATYGKLTLKFICTTGKLPVPWPTLVTTLSWGVQCFARYPD
HMKQHDFFKSAMPEGYVQERTIFFKDDGNYKTRAEVKFEGDTLVNRIELKGIDFKEDGNILGHKLEYNAIHGNVYIT
ADKQKNGIKANFGLNCNIEDGSVQLADHYQQNTPIGDGPVLLPDNHYLSTQSKLSKDPNEKRDHMVLLEFVTAAGIT
GGTLPDQLTEEQIAEFKEAFSLFDKDGDGTITTKELGTVMRSLGQNPTEAELQDMINEVDADGDGTIDFPEFLTMMA
RKMKDTDSEEEIREAFRVFDKDGNGYISAAELRHVMTNLGEKLTDEEVDEMIREADIDGDGQVNYEEFVVMMTAKEF
PPPPPPPPPPPPPPPPPPPPPPPPPPPPPPGGSMVDSSRRKFNKTGKALRAIGRLSSLESGGIGTGFPFDPHYVEVL
GERMHYVDVGPRDGTPVLFLHGNPTSSYVWRNIIPHVAPTHRCIAPDLIGMGKSDKPDLGYFFDDHVRFMDAFIEAL
GLEEVVLVIHDWGSALGFHWAKRNPERVKGIAFMEFIRPIPTWDEWPEFARETFQAFRTTDVGRKLIIDQNVFIEGT
LPMGVVRPLTEVEMDHYREPFLNPVDREPLWRFPNELPIAGEPANIVALVEEYMDWLHQSPVPKLLFWGTPGVLIPP
AEAARLAKSLPNCKAVDIGPGENLLQEDNPDLIGSEIARWLSTLEISG

>ChemoY-CaM
MVSKGEELFTGVVPILVELDGDVNGHKFSVSGEGEGDATYGKLTLKLICTTGKLPVPWPTLVTTLGYGLQCFARYPD
HMKQHDFFKSAMPEGYVQERTIFFKDDGNYKTRAEVKFEGDTLVNRIELKGIDFKEDGNILGHKLEYNYNSHNVYIT
ADKQKNGIKANFKIRHNIEDGGVQLADHYQQNTPIGDGPVLLPDNHYLSYQSKLSKDPNEKRDHMVLLEFVTAAGIT
GGTLPDQLTEEQIAEFKEAFSLFDKDGDGTITTKELGTVMRSLGQNPTEAELQDMINEVDADGDGTIDFPEFLTMMA
RKMKDTDSEEEIREAFRVFDKDGNGYISAAELRHVMTNLGEKLTDEEVDEMIREADIDGDGQVNYEEFVVMMTAKEF
PPPPPPPPPPPPPPPPPPPPPPPPPPPPPPGGSMVDSSRRKFNKTGKALRAIGRLSSLESGGIGTGFPFDPHYVEVL
GERMHYVDVGPRDGTPVLFLHGNPTSSYVWRNIIPHVAPTHRCIAPDLIGMGKSDKPDLGYFFDDHVRFMDAFIEAL
GLEEVVLVIHDWGSALGFHWAKRNPERVKGIAFMEFIRPIPTWDEWPEFARETFQAFRTTDVGRKLIIDQNVFIEGT
LPMGVVRPLTEVEMDHYREPFLNPVDREPLWRFPNELPIAGEPANIVALVEEYMDWLHQSPVPKLLFWGTPGVLIPP
AEAARLAKSLPNCKAVDIGPGENLLQEDNPDLIGSEIARWLSTLEISG

>ChemoR-CaM
MVSKGEELIKENMRMKVVMEGSVNGHQFKCTGEGEGNPYMGTQTMRIKVIEGGPLPFAFDILATSFMYGSRTFIKYP
KGIPDFFKQSFPEGFTWERVTRYEDGGVVTVMQDTSLEDGCLVYHVQVRGVNFPSNGPVMQKKTKGWEPNTEMMYPA
DGGLRGYTHMALKVDGGGHLSCSFVTTYRSKKTVGNIKMPGIHAVDHRLERLEESDNEMFVVQREHAVAKFAGLGGG

MDELYKGGTLPDQLTEEQIAEFKEAFSLFDKDGDGTITTKELGTVMRSLGQNPTEAELQDMINEVDADGDGTIDFPE
FLTMMARKMKDTDSEEEIREAFRVFDKDGNGYISAAELRHVMTNLGEKLTDEEVDEMIREADIDGDGQVNYEEFVVM
MTAKEFPPPPPPPPPPPPPPPPPPPPPPPPPPPPPPGGSMVDSSRRKFNKTGKALRAIGRLSSLESGGIGTGFPFDP
HYVEVLGERMHYVDVGPRDGTPVLFLHGNPTSSYVWRNIIPHVAPTHRCIAPDLIGMGKSDKPDLGYFFDDHVRFMD
AFIEALGLEEVVLVIHDWGSALGFHWAKRNPERVKGIAFMEFIRPIPTWDEWPEFARETFQAFRTTDVGRKLIIDQN
VFIEGTLPMGVVRPLTEVEMDHYREPFLNPVDREPLWRFPNELPIAGEPANIVALVEEYMDWLHQSPVPKLLFWGTP
GVLIPPAEAARLAKSLPNCKAVDIGPGENLLQEDNPDLIGSEIARWLSTLEISG

>ChemoL-CaM

MGLSGDQMGQIEKIFKVVYPVDDHHFKVILHYGTLVIDGVTPNMIDYFGRPYEGIAVFDGKKITVTGTLWNGNKIID
ERLINPDGSLLFRVTINGVTGWRLCERILAGGTGGSGGTGGSMVFTLEDFVGDWRQTAGYNLDQVLEQGGVSSLFQN
LGVSVTPIQRIVLSGENGLKIDIHVIIPYEVSKGEELFTGVVPILVELDGDVNGHKFSVSGEGEGDATYGKLTLKFI
CTTGKLPVPWPTLVTTLTYGVQCFSRYPDHMKQHDFFKSAMPEGYVQERTIFFKDDGNYKTRAEVKFEGDTLVNRIE
LKGIDFKEDGNILGHKLEYNYNSHNVYIMADKQKNGIKVNFKIRHNIEDGSVQLADHYQQNTPIGDGPVLLPDNHYL
STQSKLSKDPNEKRDHMVLLEFVTAAGITGGTLPDQLTEEQIAEFKEAFSLFDKDGDGTITTKELGTVMRSLGQNPT
EAELQDMINEVDADGDGTIDFPEFLTMMARKMKDTDSEEEIREAFRVFDKDGNGYISAAELRHVMTNLGEKLTDEEV
DEMIREADIDGDGQVNYEEFVVMMTAKEFPPPPPPPPPPPPPPPPPPPPPPPPPPPPPPGGSMVDSSRRKFNKTGKA
LRAIGRLSSLESGGIGTGFPFDPHYVEVLGERMHYVDVGPRDGTPVLFLHGNPTSSYVWRNIIPHVAPTHRCIAPDL
IGMGKSDKPDLGYFFDDHVRFMDAFIEALGLEEVVLVIHDWGSALGFHWAKRNPERVKGIAFMEFIRPIPTWDEWPE
FARETFQAFRTTDVGRKLIIDQNVFIEGTLPMGVVRPLTEVEMDHYREPFLNPVDREPLWRFPNELPIAGEPANIVA
LVEEYMDWLHQSPVPKLLFWGTPGVLIPPAEAARLAKSLPNCKAVDIGPGENLLQEDNPDLIGSEIARWLSTLEISG

EGFP, EBFP2, mCerulean3, Venus, mRuby2, cpNanoLuc
HaloTag7
Calmodulin
M13 peptide
Linker
Interface mutations (XFP^{A206K}, $HT7^{L271E}$, $EBFP2^{N39Y}$)

ATP sensors

>ChemoG-ATP
MVSKGEELFTGVVPILVELDGDVNGHKFSVSGEGEGDATYGKLTLKFICTTGKLPVPWPTLVTTLTYGVQCFSRYPD
HMKQHDFFKSAMPEGYVQERTIFFKDDGNYKTRAEVKFEGDTLVNRIELKGIDFKEDGNILGHKLEYNYNSHNVYIM
ADKQKNGIKVNFKIRHNIEDGSVQLADHYQQNTPIGDGPVLLPDNHYLSTQSKLSKDPNEKRDHMVLLEFVTAAGIT
GGGMKTVKVNITTPDGPVYDADIEMVSVRAESGDLGILPGHIPTKAPLKIGAVRLKKDGQTEMVAVSGGTVEVRPDH
VTINAQAAETAEGIDKERAEAARQRAQERLNSQSDDTDIRRAELALQRALNRLDVAGKANEFGGGIGTGFPFDPHYV
EVLGERMHYVDVGPRDGTPVLFLHGNPTSSYVWRNIIPHVAPTHRCIAPDLIGMGKSDKPDLGYFFDDHVRFMDAFI
EALGLEEVVLVIHDWGSALGFHWAKRNPERVKGIAFMEFIRPIPTWDEWPEFARETFQAFRTTDVGRKLIIDQNVFI
EGTLPMGVVRPLTEVEMDHYREPFLNPVDREPLWRFPNELPIAGEPANIVALVEEYMDWLHQSPVPKLLFWGTPGVL
IPPAEAARLAKSLPNCKAVDIGPGENLLQEDNPDLIGSEIARWLSTLEISG

>ChemoB-ATP
MVSKGEELFTGVVPILVELDGDVNGHKFSVRGEGEGDATYGKLTLKFICTTGKLPVPWPTLVTTLSHGVQCFARYPD
HMKQHDFFKSAMPEGYVQERTIFFKDDGTYKTRAEVKFEGDTLVNRIELKGVDFKEDGNILGHKLEYNFNSHNIYIM
AVKQKNGIKVNFKIRHNVEDGSVQLADHYQQNTPIGDGPVLLPDSHYLSTQSKLSKDPNEKRDHMVLLEFRTAAGIT
GGGMKTVKVNITTPDGPVYDADIEMVSVRAESGDLGILPGHIPTKAPLKIGAVRLKKDGQTEMVAVSGGTVEVRPDH
VTINAQAAETAEGIDKERAEAARQRAQERLNSQSDDTDIRRAELALQRALNRLDVAGKANEFGGGIGTGFPFDPHYV
EVLGERMHYVDVGPRDGTPVLFLHGNPTSSYVWRNIIPHVAPTHRCIAPDLIGMGKSDKPDLGYFFDDHVRFMDAFI
EALGLEEVVLVIHDWGSALGFHWAKRNPERVKGIAFMEFIRPIPTWDEWPEFARETFQAFRTTDVGRKLIIDQNVFI
EGTLPMGVVRPLTEVEMDHYREPFLNPVDREPLWRFPNELPIAGEPANIVALVEEYMDWLHQSPVPKLLFWGTPGVL
IPPAEAARLAKSLPNCKAVDIGPGENLLQEDNPDLIGSEIARWLSTLEISG

>ChemoR-ATP
MVSKGEELIKENMRMKVVMEGSVNGHQFKCTGEGEGNPYMGTQTMRIKVIEGGPLPFAFDILATSFMYGSRTFIKYP
KGIPDFFKQSFPEGFTWERVTRYEDGGVVTVMQDTSLEDGCLVYHVQVRGVNFPSNGPVMQKKTKGWEPNTEMMYPA
DGGLRGYTHMALKVDGGGHLSCSFVTTYRSKKTVGNIKMPGIHAVDHRLERLEESDNEMFVVQREHAVAKFAGLGGG
MDELYKGGGMKTVKVNITTPDGPVYDADIEMVSVRAESGDLGILPGHIPTKAPLKIGAVRLKKDGQTEMVAVSGGTV
EVRPDHVTINAQAAETAEGIDKERAEAARQRAQERLNSQSDDTDIRRAELALQRALNRLDVAGKANEFGGGIGTGFP
FDPHYVEVLGERMHYVDVGPRDGTPVLFLHGNPTSSYVWRNIIPHVAPTHRCIAPDLIGMGKSDKPDLGYFFDDHVR
FMDAFIEALGLEEVVLVIHDWGSALGFHWAKRNPERVKGIAFMEFIRPIPTWDEWPEFARETFQAFRTTDVGRKLII
DQNVFIEGTLPMGVVRPLTEVEMDHYREPFLNPVDREPLWRFPNELPIAGEPANIVALVEEYMDWLHQSPVPKLLFW
GTPGVLIPPAEAARLAKSLPNCKAVDIGPGENLLQEDNPDLIGSEIARWLSTLEISG

>ChemoL-ATP
MGLSGDQMGQIEKIFKVVYPVDDHHFKVILHYGTLVIDGVTPNMIDYFGRPYEGIAVFDGKKITVTGTLWNGNKIID
ERLINPDGSLLFRVTINGVTGWRLCERILAGGTGGSGGTGGSMVFTLEDFVGDWRQTAGYNLDQVLEQGGVSSLFQN
LGVSVTPIQRIVLSGENGLKIDIHVIIPYEVSKGEELFTGVVPILVELDGDVNGHKFSVSGEGEGDATYGKLTLKFI
CTTGKLPVPWPTLVTTLTYGVQCFSRYPDHMKQHDFFKSAMPEGYVQERTIFFKDDGNYKTRAEVKFEGDTLVNRIE
LKGIDFKEDGNILGHKLEYNYNSHNVYIMADKQKNGIKVNFKIRHNIEDGSVQLADHYQQNTPIGDGPVLLPDNHYL
STQSKLSKDPNEKRDHMVLLEFVTAAGITGGGMKTVKVNITTPDGPVYDADIEMVSVRAESGDLGILPGHIPTKAPL
KIGAVRLKKDGQTEMVAVSGGTVEVRPDHVTINAQAAETAEGIDKERAEAARQRAQERLNSQSDDTDIRRAELALQR
ALNRLDVAGKANEFGGGIGTGFPFDPHYVEVLGERMHYVDVGPRDGTPVLFLHGNPTSSYVWRNIIPHVAPTHRCIA
PDLIGMGKSDKPDLGYFFDDHVRFMDAFIEALGLEEVVLVIHDWGSALGFHWAKRNPERVKGIAFMEFIRPIPTWDE
WPEFARETFQAFRTTDVGRKLIIDQNVFIEGTLPMGVVRPLTEVEMDHYREPFLNPVDREPLWRFPNELPIAGEPAN
IVALVEEYMDWLHQSPVPKLLFWGTPGVLIPPAEAARLAKSLPNCKAVDIGPGENLLQEDNPDLIGSEIARWLSTLE
ISG

EGFP, EBFP2, mRuby2, cpNanoLuc
HaloTag7
F_O-F_1 Ɛ subunit
Linker
Interface mutation (EGFP^{A206K}, HT7^{L271E}, EBFP2^{N39Y})

NAD$^+$ sensors

>ChemoG-NAD

MVSKGEELFTGVVPILVELDGDVNGHKFSVSGEGEGDATYGKLTLKFICTTGKLPVPWPTLVTTLTYGVQCFSRYPD
HMKQHDFFKSAMPEGYVQERTIFFKDDGNYKTRAEVKFEGDTLVNRIELKGIDFKEDGNILGHKLEYNYNSHNVYIM
ADKQKNGIKVNFKIRHNIEDGSVQLADHYQQNTPIGDGPVLLPDNHYLSTQSKLSKDPNEKRDHMVLLEFVRAAGIT
GGTMTLEEARKRVNELRDLIRYHNYRYYVLADPEISDAEYDRLLRELKELEERFPELKSPDSPTLQVGARPLEATFR
PVRHPTRMYSLDNAFNLDELKAFEERIERALGRKGPFAYTVEHLVDGLSVNLYYEEGVLVYGATRGDGEVGEEVTQN
LLTIPTIPRRLKGVPERLEVRGEVYMPIEAFLRLNEELEERGERIFKNPRNAAAGSLRQKDPRITAKRGLRATFWAL
GLGLEEVEREGVATQFALLHWLKEKGFPVEHGYARAVGAEGVEAVYQDWLKKRRALPFEANGVAVKLDELALWRELG
YTARAPRFAIAYKFPSGGIGTGFPFDPHYVEVLGERMHYVDVGPRDGTPVLFLHGNPTSSYVWRNIIPHVAPTHRCI
APDLIGMGKSDKPDLGYFFDDHVRFMDAFIEALGLEEVVLVIHDWGSALGFHWAKRNPERVKGIAFMEFIRPIPTWD
EWPEFARETFQAFRTTDVGRKLIIDQNVFIEGTLPMGVVRPLTEVEMDHYREPFLNPVDREPLWRFPNELPIAGEPA
NIVALVEEYMDWLHQSPVPKLLFWGTPGVLIPPAEAARLAKSLPNCKAVDIGPGENLLQEDNPDLIGSEIARWLSTL
EISG

>ChemoB-NAD

MVSKGEELFTGVVPILVELDGDVNGHKFSVRGEGEGDATYGKLTLKFICTTGKLPVPWPTLVTTLSHGVQCFARYPD
HMKQHDFFKSAMPEGYVQERTIFFKDDGTYKTRAEVKFEGDTLVNRIELKGVDFKEDGNILGHKLEYNFNSHNIYIM
AVKQKNGIKVNFKIRHNVEDGSVQLADHYQQNTPIGDGPVLLPDSHYLSTQSKLSKDPNEKRDHMVLLEFRRAAGIT
GGTMTLEEARKRVNELRDLIRYHNYRYYVLADPEISDAEYDRLLRELKELEERFPELKSPDSPTLQVGARPLEATFR
PVRHPTRMYSLDNAFNLDELKAFEERIERALGRKGPFAYTVEHLVDGLSVNLYYEEGVLVYGATRGDGEVGEEVTQN
LLTIPTIPRRLKGVPERLEVRGEVYMPIEAFLRLNEELEERGERIFKNPRNAAAGSLRQKDPRITAKRGLRATFWAL
GLGLEEVEREGVATQFALLHWLKEKGFPVEHGYARAVGAEGVEAVYQDWLKKRRALPFEANGVAVKLDELALWRELG
YTARAPRFAIAYKFPSGGIGTGFPFDPHYVEVLGERMHYVDVGPRDGTPVLFLHGNPTSSYVWRNIIPHVAPTHRCI
APDLIGMGKSDKPDLGYFFDDHVRFMDAFIEALGLEEVVLVIHDWGSALGFHWAKRNPERVKGIAFMEFIRPIPTWD
EWPEFARETFQAFRTTDVGRKLIIDQNVFIEGTLPMGVVRPLTEVEMDHYREPFLNPVDREPLWRFPNELPIAGEPA
NIVALVEEYMDWLHQSPVPKLLFWGTPGVLIPPAEAARLAKSLPNCKAVDIGPGENLLQEDNPDLIGSEIARWLSTL
EISG

>ChemoR-NAD

MVSKGEELIKENMRMKVVMEGSVNGHQFKCTGEGEGNPYMGTQTMRIKVIEGGPLPFAFDILATSFMYGSRTFIKYP
KGIPDFFKQSFPEGFTWERVTRYEDGGVVTVMQDTSLEDGCLVYHVQVRGVNFPSNGPVMQKKTKGWEPNTEMMYPA
DGGLRGYTHMALKVDGGGHLSCSFVTTYRSKKTVGNIKMPGIHAVDHRLERLEESDNEMFVVQREHAVAKFAGLGGG
MDELYKGGTMTLEEARKRVNELRDLIRYHNYRYYVLADPEISDAEYDRLLRELKELEERFPELKSPDSPTLQVGARP
LEATFRPVRHPTRMYSLDNAFNLDELKAFEERIERALGRKGPFAYTVEHLVDGLSVNLYYEEGVLVYGATRGDGEVG
EEVTQNLLTIPTIPRRLKGVPERLEVRGEVYMPIEAFLRLNEELEERGERIFKNPRNAAAGSLRQKDPRITAKRGLR
ATFWALGLGLEEVEREGVATQFALLHWLKEKGFPVEHGYARAVGAEGVEAVYQDWLKKRRALPFEANGVAVKLDELA
LWRELGYTARAPRFAIAYKFPSGGIGTGFPFDPHYVEVLGERMHYVDVGPRDGTPVLFLHGNPTSSYVWRNIIPHVA
PTHRCIAPDLIGMGKSDKPDLGYFFDDHVRFMDAFIEALGLEEVVLVIHDWGSALGFHWAKRNPERVKGIAFMEFIR
PIPTWDEWPEFARETFQAFRTTDVGRKLIIDQNVFIEGTLPMGVVRPLTEVEMDHYREPFLNPVDREPLWRFPNELP
IAGEPANIVALVEEYMDWLHQSPVPKLLFWGTPGVLIPPAEAARLAKSLPNCKAVDIGPGENLLQEDNPDLIGSEIA
RWLSTLEISG

>ChemoD-NAD

MVSKGEELFTGVVPILVELDGDVNGHKFSVSGEGEGDATYGKLTLKLICTTGKLPVPWPTLVTTFGYGLMCFARYPD
HMKQHDFFKSAMPEGYVQERTIFFKDDGNYKTRAEVKFEGDTLVNRIELKGIDFKEDGNILGHKLEYNWNSHNVYIM
ADKQKNGIKVNFKIRHNIEDGSVQLADHYQQNTPIGDGPVLLPDNHYLSTQSKLSKDPNEKRDHMVLLEFVRAAGIT
GGTMTLEEARKRVNELRDLIRYHNYRYYVLADPEISDAEYDRLLRELKELEERFPELKSPDSPTLQVGARPLEATFR
PVRHPTRMYSLDNAFNLDELKAFEERIERALGRKGPFAYTVEHLVDGLSVNLYYEEGVLVYGATRGDGEVGEEVTQN
LLTIPTIPRRLKGVPERLEVRGEVYMPIEAFLRLNEELEERGERIFKNPRNAAAGSLRQKDPRITAKRGLRATFWAL
GLGLEEVEREGVATQFALLHWLKEKGFPVEHGYARAVGAEGVEAVYQDWLKKRRALPFEANGVAVKLDELALWRELG
YTARAPRFAIAYKFPSGGIGTGFPFDPHYVEVLGERMHYVDVGPRDGTPVLFLHGNPTSSYVWRNIIPHVAPTHRCI
APDLIGMGKSDKPDLGYFFDDHVRFMDAFIEALGLEEVVLVIHDWGSALGFHWAKRNPERVKGIAFMEFIRPIPTWD

EWPEFARETFQAFRTTDVGRKLIIDQNVFIEGTLWMGVVRPLTEVEMDHYREPFLNPVDREPLWRFPNELPIAGEPA
NIVALVEEYMDWLHQSPVPKLLFWGTPGVLIPPAEAARLAKSLPNCKAVDIGPGENLLQEDNPDLIGSEIARWLSTL
EISG

>ChemoL-NAD
MGLSGDQMGQIEKIFKVVYPVDDHHFKVILHYGTLVIDGVTPNMIDYFGRPYEGIAVFDGKKITVTGTLWNGNKIID
ERLINPDGSLLFRVTINGVTGWRLCERILAGGTGGSGGTGGSMVFTLEDFVGDWRQTAGYNLDQVLEQGGVSSLFQN
LGVSVTPIQRIVLSGENGLKIDIHVIIPYEVSKGEELFTGVVPILVELDGDVNGHKFSVSGEGEGDATYGKLTLKFI
CTTGKLPVPWPTLVTTLTYGVQCFSRYPDHMKQHDFFKSAMPEGYVQERTIFFKDDGNYKTRAEVKFEGDTLVNRIE
LKGIDFKEDGNILGHKLEYNYNSHNVYIMADKQKNGIKVNFKIRHNIEDGSVQLADHYQQNTPIGDGPVLLPDNHYL
STQSKLSKDPNEKRDHMVLLEFVRAAGITGGTMTLEEARKRVNELRDLIRYHNYRYYVLADPEISDAEYDRLLRELK
ELEERFPELKSPDSPTLQVGARPLEATFRPVRHPTRMYSLDNAFNLDELKAFEERIERALGRKGPFAYTVEHLVDGL
SVNLYYEEGVLVYGATRGDGEVGEEVTQNLLTIPTIPRRLKGVPERLEVRGEVYMPIEAFLRLNEELEERGERIFKN
PRNAAAGSLRQKDPRITAKRGLRATFWALGLGLEEVEREGVATQFALLHWLKEKGFPVEHGYARAVGAEGVEAVYQD
WLKKRRALPFEANGVAVKLDELALWRELGYTARAPRFAIAYKFPSGGIGTGFPFDPHYVEVLGERMHYVDVGPRDGT
PVLFLHGNPTSSYVWRNIIPHVAPTHRCIAPDLIGMGKSDKPDLGYFFDDHVRFMDAFIEALGLEEVVLVIHDWGSA
LGFHWAKRNPERVKGIAFMEFIRPIPTWDEWPEFARETFQAFRTTDVGRKLIIDQNVFIEGTLPMGVVRPLTEVEMD
HYREPFLNPVDREPLWRFPNELPIAGEPANIVALVEEYMDWLHQSPVPKLLFWGTPGVLIPPAEAARLAKSLPNCKA
VDIGPGENLLQEDNPDLIGSEIARWLSTLEISG

EGFP, EBFP2, mRuby2, ShadowG, cpNanoLuc
*tt*LigA
HaloTag7
Linker
Interface mutations (EGFP^{A206K}, EGFP^{T225R}, HT7^{L271E})
Catalytic mutations *tt*LigA (K117L, D289N)
Affinity mutations *tt*LigA (Y226W, V292A)

HT7^{P174W}

Supplementary Note 5 – Purification sequences

>Strep-tag®II + enterokinase cleavage sequence (N-terminal)

`WSHPQFEKGADDDDKVPH[…]` (pET-51b(+) plasmids)

>Poly-histidine tag sequence (C-terminal)

`[…]APGFSSISAHHHHHHHHHH`

>Poly-histidine tag + TEV cleavage sequence (N-terminal)

`HHHHHHHHHHENLYFQGGG[…]` (pET-51b(+) plasmids for crystallography)

Supplementary Note 6 – Localization sequences

>Nuclear exit signal (NES) (N-terminal or C-terminal)

```
[…]LPPLERLTL                  (pCDNA5 plasmids)
LQNELALKLAGLDINKTGGS[…]       (pAAV plasmids)
```

>Nuclear localization sequence (NLS) (C-terminal, 3 copies)

```
[…]KSGLRSRADPKKKRKVDPKKKRKVDPKKKRKVGSTGSR
```

>Exterior plasma membrane localization sequence (IgKchL[…]PDGFR$_{tm}$) (N-terminal and C-terminal)

```
METDTLLLWVLLLWVPGSTGDYPYDVPDYA[…]EQKLISEEDLNAVGQDTQEVIVVPHSLPFKVVVISAILALVVLT
IISLIILIMLWQKKPR
```

>Nuclear envelope (LaminB1) localization sequence (C-terminal)

```
[…]MATATPVPPRMGSRAGGPTTPLSPTRLSRLQEKEELRELNDRLAVYIDKVRSLETENSALQLQVTEREEVRGRE
LTGLKALYETELADARRALDDTARERAKLQIELGKCKAEHDQLLLNYAKKESDLNGAQIKLREYEAALNSKDAALAT
ALGDKKSLEGDLEDLKDQIAQLEASLAAAKKQLADETLLKVDLENRCQSLTEDLEFRKSMYEEEINETRRKHETRLV
EVDSGRQIEYEYKLAQALHEMREQHDAQVRLYKEELEQTYHAKLENARLSSEMNTSTVNSAREELMESRMRIESLSS
QLSNLQKESRACLERIQELEDLLAKEKDNSRRMLTDKEREMAEIRDQMQQQLNDYEQLLDVKLALDMEISAYRKLLE
GEEERLKLSPSPSSRVTVSRASSSRSVRTTRGKRKRVDVEESEASSSVSISHSASATGNVCIEEIDVDGKFIRLKNT
SEQDQPMGGWEMIRKIGDTSVSYKYTSRYVLKAGQTVTIWAANAGVTASPPTDLIWKNQNSWGTGEDVKVILKNSQG
EEVAQRSTVFKTTIPEEEEEEEEEAAGVVVEEELFHQQGTPRASNRSCAIM
```

>Mitochondrial localization sequence (Cox8) (N-terminal, 4 copies)

```
4x[MSVLTPLLLRGLTGSARRLPVPRAKIHSLSVLTPLLLRGLTGSARRLPVPRAKIHSL][…]
```

References

1. Grimm, J.B. et al. A general method to optimize and functionalize red-shifted rhodamine dyes. *Nat Methods* **17**, 815-821 (2020).
2. Grimm, J.B. et al. A general method to improve fluorophores for live-cell and single-molecule microscopy. *Nat Methods* **12**, 244-250, 243 p following 250 (2015).
3. Lukinavicius, G. et al. Fluorescent dyes and probes for super-resolution microscopy of microtubules and tracheoles in living cells and tissues. *Chem Sci* **9**, 3324-3334 (2018).
4. Butkevich, A.N. et al. Fluorescent Rhodamines and Fluorogenic Carbopyronines for Super-Resolution STED Microscopy in Living Cells. *Angew Chem Int Ed Engl* **55**, 3290-3294 (2016).
5. Lukinavicius, G. et al. A near-infrared fluorophore for live-cell super-resolution microscopy of cellular proteins. *Nat Chem* **5**, 132-139 (2013).
6. Wilhelm, J. et al. Kinetic and Structural Characterization of the Self-Labeling Protein Tags HaloTag7, SNAP-tag, and CLIP-tag. *Biochemistry* **60**, 2560-2575 (2021).
7. Farrants, H. et al. Chemogenetic Control of Nanobodies. *Nat Methods* **17**, 279-282 (2020).
8. Subach, O.M., Cranfill, P.J., Davidson, M.W. & Verkhusha, V.V. An enhanced monomeric blue fluorescent protein with the high chemical stability of the chromophore. *PLoS One* **6**, e28674 (2011).
9. Thestrup, T. et al. Optimized ratiometric calcium sensors for functional in vivo imaging of neurons and T lymphocytes. *Nat Methods* **11**, 175-182 (2014).
10. Fritz, R.D. et al. A versatile toolkit to produce sensitive FRET biosensors to visualize signaling in time and space. *Sci Signal* **6**, rs12 (2013).
11. Bindels, D.S. et al. mScarlet: a bright monomeric red fluorescent protein for cellular imaging. *Nat Methods* **14**, 53-56 (2017).
12. Brun, M.A. et al. A semisynthetic fluorescent sensor protein for glutamate. *Journal of the American Chemical Society* **134**, 7676-7678 (2012).
13. Sallin, O. et al. Semisynthetic biosensors for mapping cellular concentrations of nicotinamide adenine dinucleotides. *Elife* **7** (2018).
14. Chen, T.W. et al. Ultrasensitive fluorescent proteins for imaging neuronal activity. *Nature* **499**, 295-300 (2013).
15. Horikawa, K. et al. Spontaneous network activity visualized by ultrasensitive Ca(2+) indicators, yellow Cameleon-Nano. *Nat Methods* **7**, 729-732 (2010).
16. Moeyaert, B. et al. Improved methods for marking active neuron populations. *Nature communications* **9**, 4440 (2018).
17. Kotera, I., Iwasaki, T., Imamura, H., Noji, H. & Nagai, T. Reversible dimerization of Aequorea victoria fluorescent proteins increases the dynamic range of FRET-based indicators. *ACS chemical biology* **5**, 215-222 (2010).
18. Murakoshi, H., Shibata, A.C.E., Nakahata, Y. & Nabekura, J. A dark green fluorescent protein as an acceptor for measurement of Forster resonance energy transfer. *Sci Rep* **5**, 15334 (2015).
19. Yu, Q. et al. Semisynthetic sensor proteins enable metabolic assays at the point of care. *Science* **361**, 1122-1126 (2018).
20. Karplus, P.A. & Diederichs, K. Linking crystallographic model and data quality. *Science* **336**, 1030-1033 (2012).
21. McMahon, S.M. & Jackson, M.B. An Inconvenient Truth: Calcium Sensors Are Calcium Buffers. *Trends Neurosci* **41**, 880-884 (2018).
22. Nagai, T., Yamada, S., Tominaga, T., Ichikawa, M. & Miyawaki, A. Expanded dynamic range of fluorescent indicators for Ca(2+) by circularly permuted yellow fluorescent proteins. *Proc Natl Acad Sci U S A* **101**, 10554-10559 (2004).

23. Gibson, D.G. et al. Enzymatic assembly of DNA molecules up to several hundred kilobases. *Nat Methods* **6**, 343-345 (2009).
