# Supplementary material for: A general method for the development of multicolor biosensors with large dynamic ranges
Source: Nat Chem Biol. 2023 Jun 8;19(9):1147–57. doi: 10.1038/s41589-023-01350-1 (PMC10449634; doi:10.1038/s41589-023-01350-1)
Supplement: Supplementary file 2 — Reporting Summary [file 41589_2023_1350_MOESM2_ESM.pdf]

nature portfolio

Corresponding author(s): Dr. Julien Hiblot

Last updated by author(s): Apr 14, 2023

Reporting Summary

Nature Portfolio wishes to improve the reproducibility of the work that we publish. This form provides structure for consistency and transparency in reporting. For further information on Nature Portfolio policies, see our Editorial Policies and the Editorial Policy Checklist.

Statistics

For all statistical analyses, confirm that the following items are present in the figure legend, table legend, main text, or Methods section.

n/a	Confirmed	
☐	☒	The exact sample size (n) for each experimental group/condition, given as a discrete number and unit of measurement
☐	☒	A statement on whether measurements were taken from distinct samples or whether the same sample was measured repeatedly
☐	☒	The statistical test(s) used AND whether they are one- or two-sided<br>*Only common tests should be described solely by name; describe more complex techniques in the Methods section.*
☒	☐	A description of all covariates tested
☐	☒	A description of any assumptions or corrections, such as tests of normality and adjustment for multiple comparisons
☐	☒	A full description of the statistical parameters including central tendency (e.g. means) or other basic estimates (e.g. regression coefficient) AND variation (e.g. standard deviation) or associated estimates of uncertainty (e.g. confidence intervals)
☐	☒	For null hypothesis testing, the test statistic (e.g. F, t, r) with confidence intervals, effect sizes, degrees of freedom and P value noted<br>*Give P values as exact values whenever suitable.*
☒	☐	For Bayesian analysis, information on the choice of priors and Markov chain Monte Carlo settings
☒	☐	For hierarchical and complex designs, identification of the appropriate level for tests and full reporting of outcomes
☒	☐	Estimates of effect sizes (e.g. Cohen's d, Pearson's r), indicating how they were calculated

Our web collection on statistics for biologists contains articles on many of the points above.

Software and code

Policy information about availability of computer code

Data collection	Plate reader: Tecan Sparkcontrol Method Editor Version 2.2<br>Microscopy: Leica LAS X Version 3.5.7.23225 (Confocal) and LASX FLIM/FCS Version 3.5.6 (FLIM), Leica LAS X Version 3.7.1.21655 (Widefield)
Data analysis	General data analysis: GraphPad Prism (version 8.1.0), Microsoft Excel Professional Plus 2019 (16.0.10390.20024)<br>Molecular Biology: NEBaseChanger (version 1.3.3, nebasechanger.neb.com), Tm Calculator (version 1.15.0, tmcalculator.neb.com), Geneious (version 11.1.5).<br>Image analysis: ImageJ 1.53p, Leica LAS X 3.5.7.23225 (Confocal) and LASX FLIM/FCS 3.5.6 (FLIM), Leica LAS X Version 3.7.1.21655 (Widefield)<br>X-ray crystallography: XDS (VERSION Mar 15, 2019 BUILT=20190315 and VERSION Jan 31, 2020 BUILT=20200131), Refmac5 (versions 5.8.0258), Phaser (version 2.8.2 and 2.8.3), Coot (version 0.8.9.2), PHENIX (versions 1.15.2-3472 and 1.17.1-3660) and the MolProbity implemented therein (version 4.4), PyMOL version 2.1.1.

For manuscripts utilizing custom algorithms or software that are central to the research but not yet described in published literature, software must be made available to editors and reviewers. We strongly encourage code deposition in a community repository (e.g. GitHub). See the Nature Portfolio guidelines for submitting code & software for further information.

Data

Policy information about availability of data

All manuscripts must include a data availability statement. This statement should provide the following information, where applicable:

- Accession codes, unique identifiers, or web links for publicly available datasets
- A description of any restrictions on data availability
- For clinical datasets or third party data, please ensure that the statement adheres to our policy

Plasmids encoding certain ChemoX constructs have been deposited on Addgene. Accession codes can be found in Supplementary Table S14 . The X-ray crystal structures of ChemoG1-TMR, ChemoG5-TMR and HaloTag7-Cy3 have been deposited to the PDB with deposition codes 8B6S, 8B6T and 8B6R, respectively. Correspondence and requests for materials should be addressed to J.H. The data supporting the findings of this study are available within the article and its Supplementary Information. Additional data are available from the corresponding author upon reasonable request.
The crystal structure of HaloTag-TMR (PDB ID: 6Y7A), GFP (PDB ID: 1GFL), HaloTag-P174W-TMR (PDB ID: 6ZVV) and of the LigA from Enterococcus faecalis (efLigA) bound to NAD+ (PDB ID: 1TAE) were available from the pdb.

Human research participants

Policy information about studies involving human research participants and Sex and Gender in Research.

Reporting on sex and gender	Sex and gender was not necessary for the presented experiments, as the data is not relevant to a clinical trial.
Population characteristics	Population characteristics was not necessary for the presented experiments, as the data is not relevant to a clinical trial.
Recruitment	Recruitment was not necessary for the presented experiments, as the data is not relevant to a clinical trial.
Ethics oversight	Ethics oversight was not necessary for the presented experiments, as the data is not relevant to a clinical trial.

Note that full information on the approval of the study protocol must also be provided in the manuscript.

Field-specific reporting

Please select the one below that is the best fit for your research. If you are not sure, read the appropriate sections before making your selection.

☒ Life sciences ☐ Behavioural & social sciences ☐ Ecological, evolutionary & environmental sciences

For a reference copy of the document with all sections, see nature.com/documents/nr-reporting-summary-flat.pdf

Life sciences study design

All studies must disclose on these points even when the disclosure is negative.

Sample size	Sample sizes were based on experiences in prior studies or samples were acquired until a clear trend was evident.<br>Treatments performed in the study were previously characterized in the literature.<br>For Calcium sensing, see 10.1073/pnas.0400417101 [histamine] or 10.1038/s41467-021-27249-w [ionomycin].<br>For ATP sensing, see 10.1021/cb900263z or 10.1021/acssensors.9b02475 [2-DG].<br>For NAD+ sensing, see 10.7554/eLife.32638, 10.1126/science.aad5168 [FK866] or 10.1523/JNEUROSCI.5552-09.2010 [MNNG].
Data exclusions	No data was excluded.
Replication	In vitro measurements were performed in technical triplicates or as indicated. Microscopy experiments were performed on three independent sample preparations and different field of views unless stated otherwise. All replicates were successful.
Randomization	Randomization is not relevant to the study, as all experiments were performed with cell lines. No experiments involved allocation of different samples, organism, or participants into experimental groups.
Blinding	Blinding is not relevant to the study because no experiments involved allocation of different samples, organisms, or participants into experimental groups.

Reporting for specific materials, systems and methods

We require information from authors about some types of materials, experimental systems and methods used in many studies. Here, indicate whether each material, system or method listed is relevant to your study. If you are not sure if a list item applies to your research, read the appropriate section before selecting a response.

Materials & experimental systems

n/a	Involved in the study
☒	☐ Antibodies
☐	☒ Eukaryotic cell lines
☒	☐ Palaeontology and archaeology
☐	☒ Animals and other organisms
☒	☐ Clinical data
☒	☐ Dual use research of concern

Methods

n/a	Involved in the study
☒	☐ ChIP-seq
☒	☐ Flow cytometry
☒	☐ MRI-based neuroimaging

Eukaryotic cell lines

Policy information about cell lines and Sex and Gender in Research

Cell line source(s)	U-2 OS Flp-In T-REx cell line (Molecular and Cellular Biology 2006, 26 (12), 4642-4651) - from Blacklow lab. Department of Pathology, Brigham and Women's Hospital and Harvard Medical School, Boston, MA 02115, USA.<br>HeLa Kyoto cells (RRID:CVCL_192219, Cell 2009, 126 (3), 473-484) - from Ellenberg lab. Cell Biology and Biophysics Unit, European Molecular Biology Laboratory, Heidelberg, Germany.<br>HEK293 from Leibniz Institute DSMZ German Collection of Microorganisms and Cell Cultures, Reference ACC-305.
Authentication	Cell lines were not further authenticated.
Mycoplasma contamination	Cell lines have been tested by PCR and were negative.
Commonly misidentified lines (See ICLAC register)	Not applicable as no commonly misidentified cell lines were used.

Animals and other research organisms

Policy information about studies involving animals; ARRIVE guidelines recommended for reporting animal research, and Sex and Gender in Research

Laboratory animals	Postnatal day 0-1 Wistar rats of either sex.
Wild animals	The study did not involve wild animals
Reporting on sex	Animal sex did not matter for study design as only isolated neurons were used.
Field-collected samples	The study did not involve samples collected from the field.
Ethics oversight	Procedures were performed in accordance with the Animal Welfare Act of the Federal Republic of Germany (Tierschutzgesetz der Bundesrepublik Deutschland, TierSchG) and the Animal Welfare Laboratory Animal Regulations (Tierschutzversuchsverordnung). According to the TierSchG and the Tierschutzversuchsverordnung no ethical approval from the ethics committee is required for the procedure of sacrificing rodents for subsequent extraction of tissues, as performed in this study. The procedure for sacrificing rats performed in this study was supervised by animal welfare officers of the Max Planck Institute for Medical Research (MPImF) and conducted and documented according to the guidelines of the TierSchG (permit number assigned by the MPImF: MPI/T-35/18).

Note that full information on the approval of the study protocol must also be provided in the manuscript.
